# Halide Exchange Reactions in Large Halide Perovskite Nanocrystals

**DOI:** 10.1021/acsnano.6c10843

**Published:** 2026-10-29

**Authors:** Zhanzhao Li, Yurii P. Ivanov, Nikolaos Livakas, Lorenzo Tallarini, Jordi Llusar, Aswin Asaithambi, Andrea Toma, Dmitry Baranov, Giorgio Divitini, Liberato Manna

**Affiliations:** † Nanochemistry, Istituto Italiano di Tecnologia, Via Morego 30, 16163 Genova, Italy; ‡ State Key Laboratory of Coordination Chemistry, School of Chemistry, Nanjing University, Nanjing 210023, China; § Electron Spectroscopy and Nanoscopy, Istituto Italiano di Tecnologia, Via Morego 30, 16163 Genova, Italy; ∥ Division of Chemical Physics and NanoLund, Department of Chemistry, 5193 Lund University, P.O. Box 124, Lund SE-22100, Sweden; ⊥ Clean Room Facility, Istituto Italiano di Tecnologia, Via Morego 30, 16163 Genova, Italy

**Keywords:** Nanocrystals, halide perovskites, hollow nanocubes, anion exchange, core−shell, heterostructures

## Abstract

We have studied halide exchange reactions in large colloidal nanocrystals of lead halide perovskites, with sizes (∼40–60 nm) that are intermediate between those of typical nanocrystals synthesized and studied by the community (5–10 nm) and those of microcrystals or thin films. This size regime still allows an investigation of the nanocrystals with techniques used for colloidal nanocrystals while enabling the observation of phenomena such as phase segregation, generation of planar defects, strain accumulation and relaxation, which are harder to identify in smaller nanocrystals, also given their high instability under the electron beam. We compare nanocubes with hollow nanocubes, and highlight how the presence of the hollow region can influence structural and morphological changes during the reactions. Notably, we report core–shell epitaxial CsPbI_3_/CsPbCl_3_ heterostructures by partial I^–^ to Cl^–^ exchange on CsPbI_3_ nanocrystals. Transient absorption spectroscopy provides evidence consistent with electronic coupling and exciton transfer between the Cl-rich shell and I-rich core in these heterostructures, evidenced by the accelerated bleach decay of the CsPbCl_3_ component and the delayed population of the CsPbI_3_ band edge.

## Graphical abstract

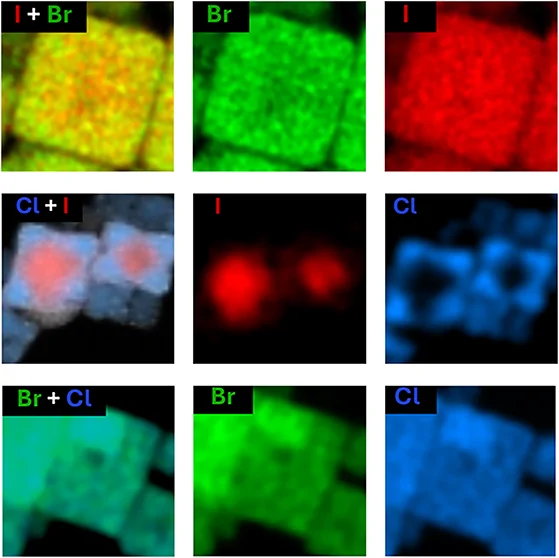

## Introduction

In colloidal nanocrystals, building epitaxial heterostructures such as core–shells provides a means to study and tune exciton dynamics, which might unlock superior optoelectronic performance.^1–5^ This procedure is well established for nanocrystals of II–VI, III–V and IV–VI semiconductors^6^ but is more challenging for halide perovskites, where the high ionic mobility tends to produce alloyed phases.^7–10^ For example, in halide exchange reactions in lead halide perovskite nanocrystals the high mobility of the halide ions (Cl^–^, Br^–^, I^–^) typically results in the rapid formation of alloys, as generally observed in CsPb­(Br:I)_3_ and CsPb­(Br:Cl)_3_ nanocrystals.^7,8^ Also, the exchange mechanism is highly direction-dependent, adding further complexity to controlling composition and structure.^9^ To date, the fabrication of well-defined, phase-separated interfaces in halide perovskites has been largely confined to the microscopic regime (microwires, microplates),^11–14^ where the longer diffusion paths help to keep the two phases separated. Recent works have studied the Cl^–^ → I^–^ exchange reaction in CsPbCl_3_ nanocrystals,^15–17^ where a substantial ionic size mismatch between Cl^–^ and I^–^ ions (∼19.5%) leads to a “miscibility gap” in the CsPbCl_3_/CsPbI_3_ system that, under specific conditions (for example a pre-existing epitaxial interface with another material^16^), can favor the formation of segregated CsPbCl_3_ and CsPbI_3_ domains within a single nanocrystal.

The small size (5–10 nm) of the nanocrystals typically studied in halide exchange reactions and their relative instability under the electron beam make the observation of intermediate structures difficult. In this work, we employ large perovskite nanocrystals (∼40–60 nm in edge size) in both “regular nanocube” and “hollow nanocube” geometries. These samples have unique shapes (with a cube systematically compared to a hollow nanocube) and sizes, being intermediate between those of thin films/microcrystals and those of the small nanocrystals.^8,18^ Yet, such large nanocrystals can still be studied both as colloidal suspensions and under the electron microscope with no special sample preparation. Their relatively large volumes can be harnessed to capture intermediate samples with different compositions in more detail and to better evaluate how strain relaxation can lead to the formation of defects and/or overall shape evolution. Such insights are fundamental for tuning the composition of large perovskite nanocrystals, as they are attracting increasing attention as light sources for quantum technology.^19–22^

In this work we have carried out a systematic investigation of halide exchange reactions on such large nanocrystals and compared the structural, morphological and optical features of the starting, intermediate and final nanocrystals. We show that the Br^–^ → Cl^–^ exchange follows the predictable path that goes through intermediate alloy phases, due to the minimal difference in ionic radii between Br^–^ and Cl^–^, while the I^–^ → Br^–^ exchange results in the initial formation of partially alloyed heterostructures, followed by homogenization on a time scale of hours. Also, the intermediate and complete exchange samples preserve their initial shapes in both regular and hollow geometries. More interesting dynamics are observed in the I^–^ → Cl^–^ exchange, which starts at the corners of the cubes for both hollow and regular nanocubes and leads to core–shell CsPbI_3_/CsPbCl_3_ epitaxial heterostructures that are stable enough (for a few days) to enable their structural and spectroscopic analysis. Both hollow and regular nanocubes at intermediate stages of exchange contain planar defects consisting of CsI bilayers connected to Pb–Cl planes of the perovskite, which can be considered part of the unit cell plane-stacking sequence of the Cs_2_PbI_2_Cl_2_ Ruddlesden–Popper (RP) phase. We also observe channels on the surface of the nanocrystals in both samples. Upon complete I^–^ → Cl^–^ exchange, the main feature of the hollow nanocubes is the presence of channels connecting the inner surface to the outer surface, while the initially regular nanocubes tend to develop inner hollow regions, planar defects, and channels. Finally, the reverse Cl^–^ → I^–^ exchange starting from CsPbCl_3_ is initiated by the pervasive formation of CsI bilayers on the surface of the regular nanocubes, while in hollow nanocubes these layers reach deep into the inner surface as well, followed by the formation of CsPbI_3_ domains at the corners in the partial exchange case. Upon complete exchange to CsPbI_3_ the final overall shapes are significantly different from those of the starting nanocrystals.

All these transformations are interpreted as occurring due to the various possible pathways of strain relaxation in the two types of geometry (regular nanocube versus hollow nanocube), differences in the elastic properties of the perovskite lattices, and variations in halide ion diffusivities. They highlight the richness of structures, compositions, and transformation pathways that can be seen in large nanocrystals and provide additional valuable insights into halide exchange reactions in halide perovskites.

## Results and Discussion

In all the experiments presented and discussed in this section, we always started with two types of perovskite nanocube samples, one presenting a hollow region at its center, and one having a standard “non-hollow”, hence “regular”, cubic shape. Their synthesis is described in detail in the Experimental Section, where a scheme of the sequences of preparation of the initial samples is also presented.

### Br^–^ → Cl^–^ Exchange

Representative low magnification transmission electron microscopy (TEM) images of the starting hollow CsPbBr_3_ nanocubes and regular CsPbBr_3_ nanocubes are reported in Figure 1a,e. On both samples, the Br^–^ → Cl^–^ exchange was conducted by gradually adding controlled amounts of PbCl_2_ dissolved in a mixture of oleic acid and oleylamine, corresponding to overall Cl/Br feed ratios of 0.2, 0.4, 0.6, 0.8, and finally an excess amount of Cl^–^ to achieve complete exchange (see details in the Experimental Section). For both samples, the photoluminescence (PL) spectra, recorded soon after each addition step of the PbCl_2_ solution, evidenced a single emission peak that gradually shifted from 2.37 eV (starting sample) up to 2.99 eV (final sample with excess Cl^–^ added, Cl/Br = 1.2) (Figure 1b,f). This is in line with previous reports on Br^–^ → Cl^–^ exchange in smaller nanocrystals, highlighting that this reaction is generally fast at room temperature and quickly leads to alloy compositions with no miscibility gaps, due to the small difference in ionic radii between Br^–^ and Cl^–^ ions.^23^ In the hollow nanocubes, the full width at half-maximum (FWHM) of the PL peaks for the intermediate exchange samples was always larger than for the regular nanocubes (Table S1). This might be due to inhomogeneous alloying driven by the presence of the inner surface: the process can lead to regions with slightly different halide compositions inside the hollow nanocubes. Also, given the broad distribution of sizes within this sample (Figure S1 of the Supporting Information, SI) the broader PL may be due to a size-dependent rate of exchange, which should be faster in the smaller nanocrystals. In any case, if the hollow nanocubes at an intermediate stage of exchange were left to age as colloidal suspensions, their PL peak narrowed within hours (Figure S2).

**Figure 1:**
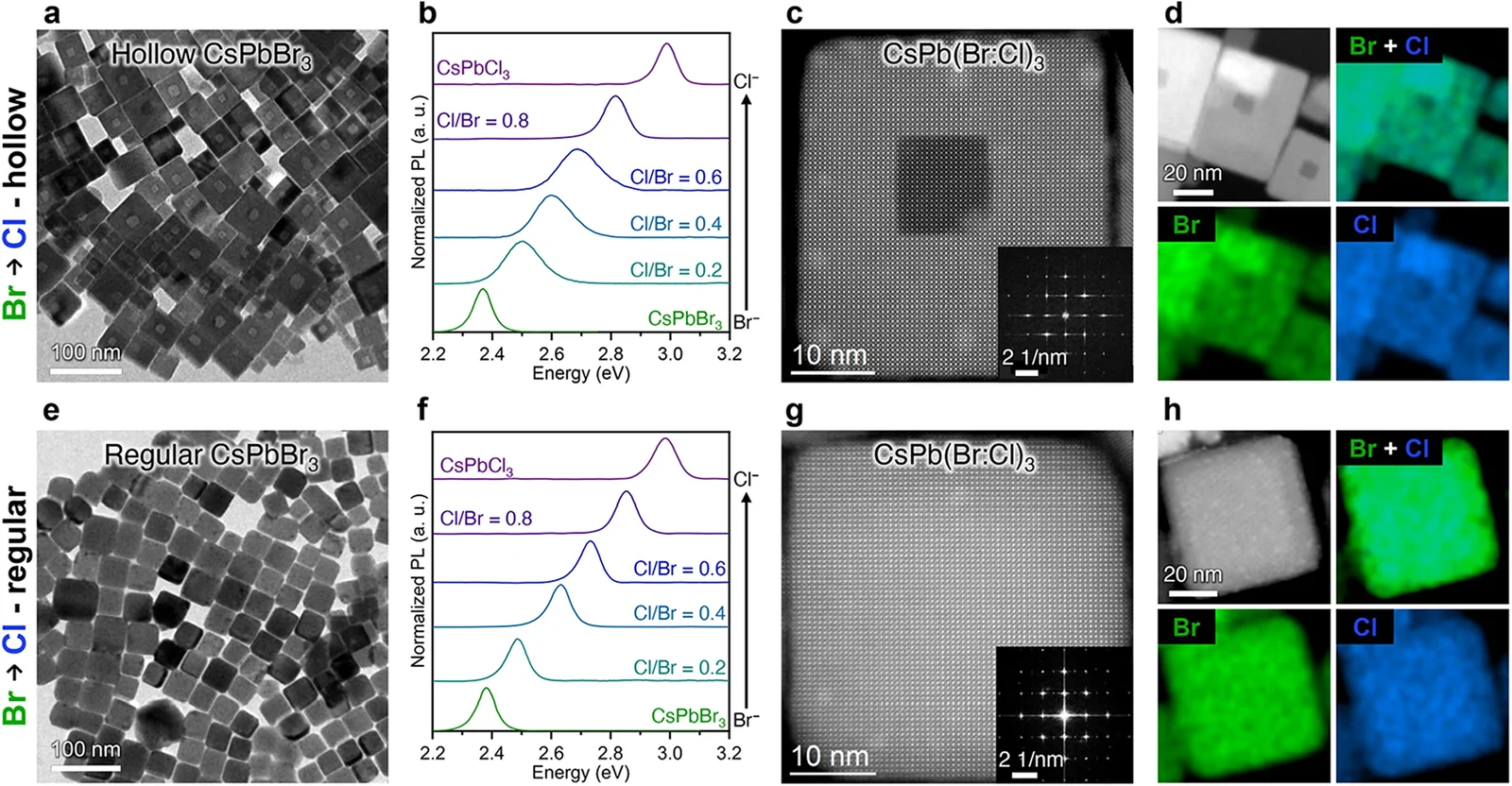
(a) TEM image of the starting hollow CsPbBr_3_ nanocubes. (b) PL spectra of hollow CsPbBr_3_, CsPb­(Br:Cl)_3_ (Cl/Br = 0.2, 0.4, 0.6, 0.8) and hollow CsPbCl_3_ nanocubes following the Br^–^ → Cl^–^ exchange. (c) HAADF-STEM image (inset in c: corresponding FFT pattern) and (d) STEM-EDS elemental maps of hollow CsPb­(Br:Cl)_3_ nanocubes (Cl/Br = 0.4). (e) TEM image of the starting regular CsPbBr_3_ nanocubes. (f) PL spectra of the regular CsPbBr_3_, CsPb­(Br:Cl)_3_ (Cl/Br = 0.2, 0.4, 0.6, 0.8) and regular CsPbCl_3_ nanocubes. (g) HAADF-STEM image (inset in g: corresponding FFT pattern) and (h) STEM-EDS elemental maps of the regular CsPb­(Br:Cl)_3_ nanocubes (Cl/Br = 0.4).

High-angle annular dark-field high-resolution scanning TEM (HAADF-STEM) analysis of representative nanocubes at an intermediate state of exchange (Cl/Br = 0.4, Figure 1c,g) revealed that the nanocubes remained highly crystalline (see the Fast Fourier Transform (FFT) of the image in the inset) and preserved their initial shape at the intermediate stages (see also Figures S3 and S4). Elemental maps by energy dispersive X-ray spectroscopy (STEM-EDS) (Figure 1d,h) are indicative of a relatively homogeneous halide distribution across the nanocrystals for both samples. X-ray diffraction (XRD) patterns of the dried powders for both samples indicate the presence of a single alloyed phase, with reflections monotonically following the conversion between the CsPbBr_3_ and the CsPbCl_3_ phase, featuring broadened peaks (Figure S5). Both samples preserved their initial morphology up to the complete Br^–^ → Cl^–^ exchange, as seen from TEM images (Figures S3c and S4c).

We measured the PL quantum yield (PLQY, Φ_PL_) and amplitude-weighted average PL decay time (τ_avg_) of colloidal suspensions of all these samples at the various stages (initial, intermediate, final, see Table 1 and Figure S6). The fitting parameters of the PL decay spectra are reported in Table S3. Since the decays are multiexponential, with amplitudes and individual components that vary considerably from sample to sample, τ_avg_ is used here only as an empirical ensemble descriptor for comparing samples, and not as a radiative or excited-state lifetime. The hollow nanocubes generally exhibited a longer τ_avg_ than the regular nanocubes, despite having lower PLQY values, throughout the exchange. Specifically, the initial CsPbBr_3_ nanocrystals had τ_avg_ values of 80 ns (hollow nanocubes) and 55 ns (regular nanocubes) and PLQYs of 35% and 44%, respectively. The Cl^–^ exchange led to a progressive decrease of both τ_avg_ and PLQY, reaching the lowest values in the fully exchanged CsPbCl_3_ nanocrystals (τ_avg_ = 9–14 ns and PLQY ≈ 1%) in both hollow and regular nanocubes. The very low PLQY suggests that nonradiative losses dominate at the ensemble level upon chloride incorporation. The long τ_avg_ values of both hollow and regular nanocubes, compared to those of the more conventional, small perovskite nanocubes (5–10 nm in size), are qualitatively in line with the slower PL decays reported for large nanocrystals, which have been attributed to a size-dependent contribution from symmetry-forbidden s-p transitions.^24–26^

**Table 1:** PLQY and τ_avg_ of Starting, Intermediate, and Final Samples in the Br^–^ → Cl^–^ Exchange

**Sample**	**PLQY (%)**	**τ**_**avg**_ **(ns)**
**Hollow nanocubes**		
CsPbBr_3_	35 ± 5	80
CsPb(Br:Cl)_3_(Cl/Br = 0.4)	20 ± 3	32
CsPb(Br:Cl)_3_(Cl/Br = 0.6)	8 ± 2	25
CsPbCl_3_	∼1	14
**Regular nanocubes**		
CsPbBr_3_	44 ± 5	55
CsPb(Br:Cl)_3_(Cl/Br = 0.4)	22 ± 3	18
CsPb(Br:Cl)_3_(Cl/Br = 0.6)	13 ± 2	12
CsPbCl_3_	∼1	9

### I^–^ → Br^–^ Exchange

For these experiments we started from a sample of hollow CsPbI_3_ nanocubes and a sample of regular CsPbI_3_ nanocubes (again, see the Experimental Section for details on their syntheses). Representative low magnification TEM images of these samples are reported in Figure 2a,f respectively. On both samples, the I^–^ → Br^–^ exchange was conducted by gradually adding controlled amounts of PbBr_2_ dissolved in a mixture of oleic acid and oleylamine, corresponding to overall Br/I feed ratios of 0.2, 0.4, 0.6, 0.8, and finally an excess amount of Br^–^ to achieve complete exchange. In both cases, the PL spectra recorded soon after the addition of the PbBr_2_ solution presented two separate peaks (Figure 2b,g): a first peak roughly matching the CsPbI_3_ emission, albeit blue-shifted and broadened, and a second peak quite close to the pure CsPbBr_3_ phase in case of hollow nanocubes, whereas in the regular nanocubes case this second peak matched an alloy composition. In all cases studied, if these samples were left to age as colloidal suspensions, the double peak features merged into a single peak. Tracking of the PL spectra over time revealed homogenization into an alloyed phase, with the two peaks shifting and merging within 1–2 h for hollow nanocubes and within 1 h for regular nanocubes (Figure S7). This points to slower I^–^ → Br^–^ exchange kinetics compared to the Br^–^ → Cl^–^ cases discussed earlier (Figure 2c,h). There are two possible, nonmutually exclusive explanations for the double peak features: (i) nanocrystals of different sizes within the same sample have different exchange rates; (ii) domains of different halide compositions are initially formed within individual nanocrystals. These two hypotheses could not be easily disentangled by XRD analysis, which simply evidenced diffraction peaks at positions between CsPbI_3_ and CsPbBr_3_ in intermediate exchange samples, for both hollow and regular cubes (Figure S8). Unfortunately, we were not able to isolate nanocrystals of different sizes by size-selective precipitation in order to check their optical properties separately, since all the nanocrystals precipitated out of the solution phase even at moderate centrifugation speeds.

**Figure 2:**
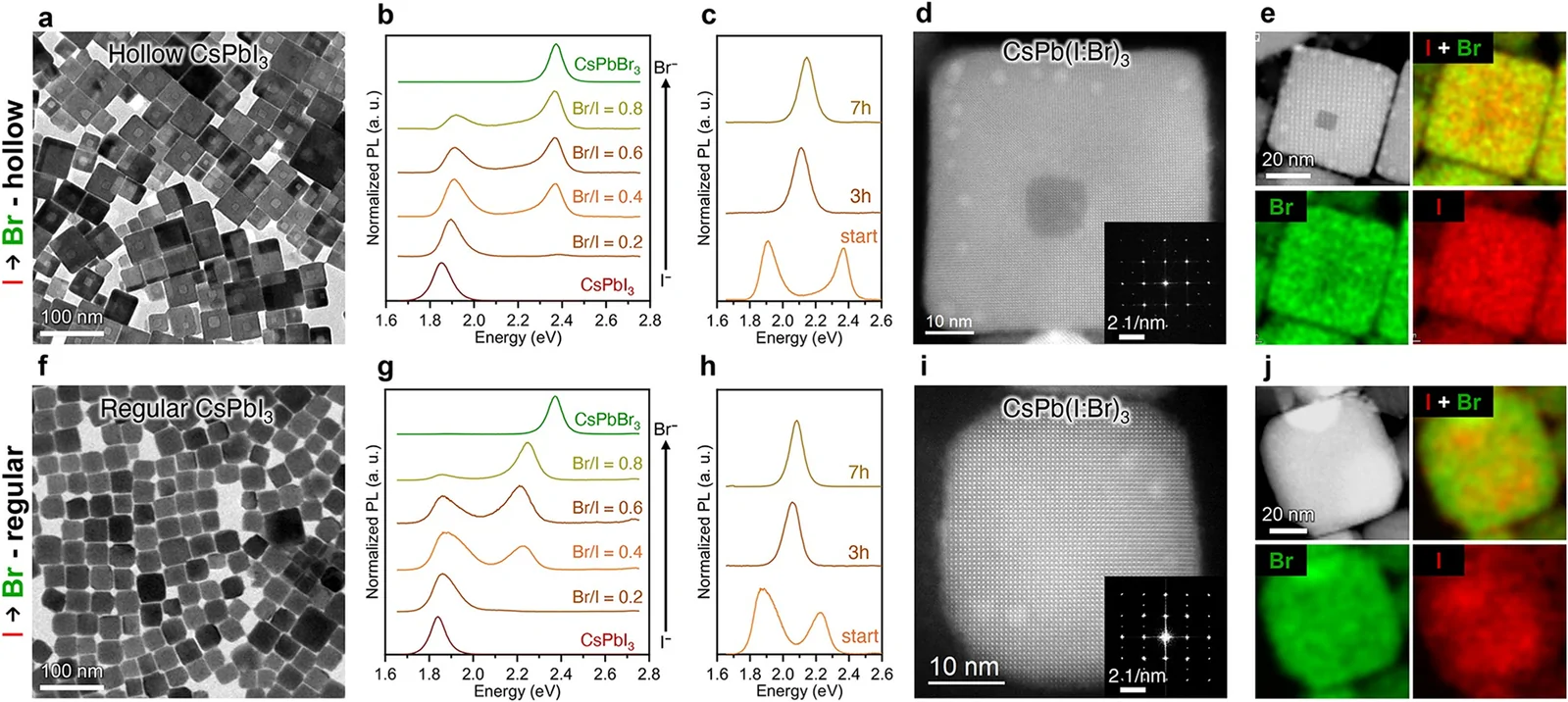
(a) TEM image of the hollow CsPbI_3_ nanocubes. (b) PL spectra of the hollow CsPbI_3_, CsPb­(I:Br)_3_ (Br/I = 0.2, 0.4, 0.6, 0.8) and hollow CsPbBr_3_ nanocubes following I^–^ → Br^–^ exchange. (c) Time evolution of the PL spectra of the intermediate CsPb­(Br:I)_3_ (Br/I = 0.4) hollow nanocube sample. (d) HAADF-STEM image (inset in d: corresponding FFT pattern) and (e) EDS elemental maps of CsPb­(Br:I)_3_ (Br/I = 0.4) hollow nanocubes. (f) TEM image of the regular CsPbI_3_ nanocubes. (g) PL spectra of the regular CsPbI_3_, CsPb­(I:Br)_3_ (Br/I = 0.2, 0.4, 0.6, 0.8) and regular CsPbBr_3_ nanocubes following I^–^ → Br^–^ exchange. (h) Time evolution of the PL spectra of the intermediate regular CsPb­(Br:I)_3_ (Br/I = 0.4) nanocube sample. (i) HAADF-STEM image (inset in i: corresponding FFT pattern) and (j) EDS elemental maps of the regular CsPb­(Br:I)_3_ (Br/I = 0.4) nanocubes.

HAADF-STEM analyses of representative nanocrystals from both samples at intermediate exchange (Br/I = 0.4), along with the corresponding FFT analyses (inset of Figure 2d,i), confirmed high crystallinity and preservation of the pristine shapes. EDS elemental maps evidenced partial halide segregation in both samples, corresponding to inhomogeneous alloying (Figure 2e,j). In agreement with the optical behavior, EDS analysis confirmed that over time a homogeneous halide composition was reached in both samples (Figure S9). We interpret both the slower exchange kinetics and the presence of segregated halide regions in the I^–^ → Br^–^ exchange compared to the Br^–^ → Cl^–^ case as possibly due to a larger volume difference between the CsPbBr_3_ and CsPbI_3_ phases (16%) compared to that between the CsPbBr_3_ and CsPbCl_3_ phases (12%), see Table S2, which affects the strain relaxation during the exchange reaction. Finally, as in the previous Br^–^ → Cl^–^ exchange experiments, both samples tended to preserve their initial shapes up to the complete I^–^ → Br^–^ exchange (Figures S10 and S11).

We measured the PLQY and τ_avg_ of colloidal suspensions of the samples at all stages (initial, intermediate, final, see Table 2 and Figure S12). The fitting parameters of the PL decay spectra are reported in Table S4. As in the Br^–^ → Cl^–^ series above (Table 1), the hollow nanocubes exhibited consistently longer τ_avg_ than the regular ones at every stage, despite a comparable or lower PLQY (Table 2). Like the Br^–^ → Cl^–^ exchange, the I^–^ → Br^–^ exchange proceeds via the formation of an alloy (Figure 2e,j), thus, limited strain accumulation and few additional defects are expected during this process. Both PLQY and τ_avg_ decrease from their initial CsPbI_3_ values through the alloyed intermediates. In the final CsPbBr_3_ composition, the PLQY partially recovers–nearly completely for the regular nanocubes (44 ± 5% vs the initial 45 ± 6%) but only partially for the hollow ones (35 ± 5% vs the initial 42 ± 5%)–whereas τ_avg_ continues to decrease, reaching its lowest values of the whole series in both geometries. We report this trend descriptively: given the multiexponential decays and the substantial evolution of their components along the series (Table S4), τ_avg_ cannot be assigned to a specific radiative or nonradiative pathway without a dedicated kinetic model. The PLQY drop in the intermediate mixed-halide stages, on the other hand, points to stronger nonradiative losses at the ensemble level. Mixed-halide compositions of this kind have been reported to be susceptible to the formation of halide vacancies, which can act as deep trap states–a loss mechanism that would no longer apply once the sample reaches the pure CsPbBr_3_ phase.^28^ Finally, the incomplete PLQY recovery in the hollow nanocubes could relate to their larger surface area (both outer and inner) compared to the regular nanocubes, which may support a higher density of dangling bonds and residual nonradiative surface vacancies after the exchange.^28,30^

**Table 2:** PLQY and τ_avg_ of Starting, Intermediate, and Final Samples in the I^–^ → Br^–^ Exchange Reaction

**Sample**	**PLQY (%)**	**τ**_**avg**_ **(ns)**
**Hollow nanocubes**		
CsPbI_3_	42 ± 5	442
CsPb(I:Br)_3_(Br/I = 0.2)	27 ± 3	337
CsPb(I:Br)_3_(Br/I = 0.4)	25 ± 3	251
CsPbBr_3_	35 ± 5	80
**Regular nanocubes**		
CsPbI_3_	45 ± 6	293
CsPb(I:Br)_3_(Br/I = 0.2)	31 ± 4	178
CsPb(I:Br)_3_(Br/I = 0.4)	30 ± 4	132
CsPbBr_3_	44 ± 5	55

### I^–^ → Cl^–^ Exchange

In a third series of experiments, we investigated the direct I^–^ → Cl^–^ exchange. This was conducted by gradually adding controlled amounts of chloride precursor (PbCl_2_ dissolved in a mixture of oleic acid and oleylamine solution; see details in the Experimental Section), corresponding to Cl/I feed ratios of 0.2, 0.4, 0.6, and 0.8, or as an excess amount (Cl/I = 1.2) to achieve complete Cl^–^ exchange. As these experiments led to the most interesting results of this work, we discuss first in detail the exchange on the hollow CsPbI_3_ nanocubes and then we continue with the exchange on the regular CsPbI_3_ nanocubes. During the exchange on the hollow CsPbI_3_ nanocubes, the evolution of the optical absorption spectra (Figure 3a) indicated the copresence of CsPbCl_3_ and CsPbI_3_ phases. In the spectra, the features in the red region, ascribed to CsPbI_3_, progressively decreased in intensity, while those in the violet region, ascribed to CsPbCl_3_, progressively increased in intensity. The absorption for CsPbCl_3_ was red-shifted through the process from partial to full Cl^–^ exchange, which might be due to I^–^ alloying or to the extensive presence of structural defects and spurious phases. In the emission spectra (Figure 3b), the peak from the CsPbI_3_ phase was broadened and slightly blue-shifted compared to the pristine CsPbI_3_ sample (possibly due to very limited Cl^–^ alloying or strain). Only at the end of the exchange was it possible to observe some emission from CsPbCl_3_ and the disappearance of the CsPbI_3_ emission. In the intermediate exchange samples, the emission from CsPbCl_3_ was likely quenched due to efficient exciton transfer from the CsPbCl_3_ regions to the CsPbI_3_ regions, as the two materials form a type-I band alignment.^16^

**Figure 3:**
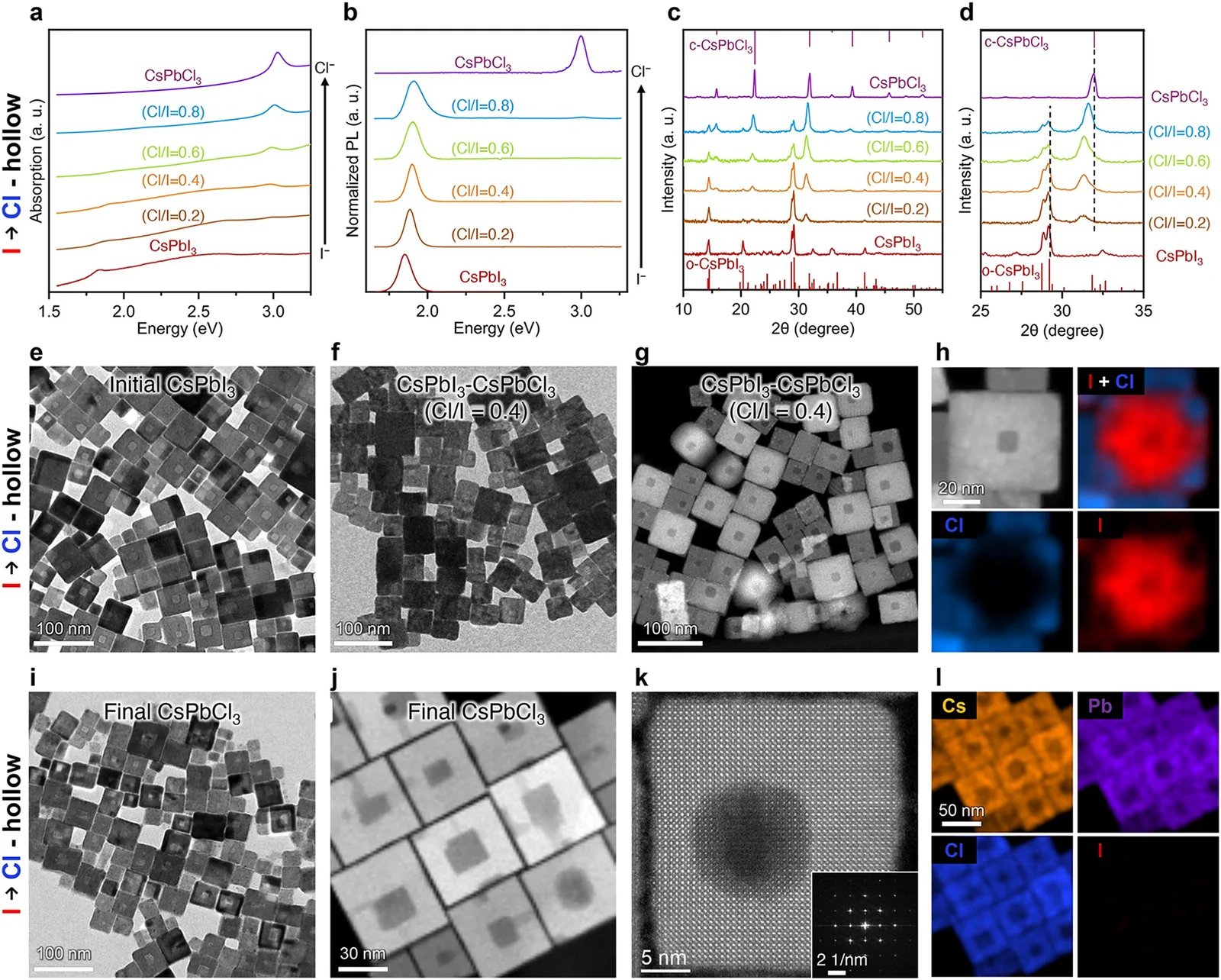
(a) Optical absorption spectra, (b) PL spectra and (c, d) XRD patterns and detail of the 2Θ = 25°–35° range for hollow CsPbI_3_, CsPbI_3_/CsPbCl_3_ (Cl/I = 0.2, 0.4, 0.6, 0.8) and hollow CsPbCl_3_ nanocubes. In (c, d) the red reference marks are for orthorhombic CsPbI_3_ (ICSD number 69423) and the purple ones are for cubic CsPbCl_3_ (ICSD number 29072). (e, f) Low magnification TEM images of (e) starting CsPbI_3_ nanocubes and (f) intermediate exchange (Cl/I = 0.4) hollow nanocubes. (g) HAADF-STEM image and (h) EDS elemental maps of intermediate exchange (Cl/I = 0.4) hollow nanocubes. (i) Low magnification TEM, (j, k) HAADF-STEM images and (l) EDS elemental maps of the fully Cl^–^ exchanged hollow nanocubes. The inset in (k) is the corresponding FFT pattern.

The coexistence of the two phases in the intermediate exchange samples was corroborated by XRD analysis (Figure 3c): the earlier the stage of exchange, the more the reflections from CsPbCl_3_ were shifted to lower angles and broadened. This is especially evident for the peak ascribed to the (112) planes of CsPbCl_3_, which in CsPbCl_3_ is positioned at 31.9° while in the earliest exchange sample is at 31.3°. Higher interplanar distances are indicative of either partial I^–^ alloying in the CsPbCl_3_ domains or high tensile strain (or both). This peak gradually moved to 31.9° in later exchange samples (Figure 3d). Figure 3e,f present low magnification TEM images of the starting samples and of a sample at an intermediate stage of exchange (Cl/I = 0.4). Despite the large difference in volume between the CsPbCl_3_ and CsPbI_3_ phases, the exchange, at least at this stage, did not seem to lead to dramatic shape changes in the nanocrystals. This was also confirmed by HAADF-STEM analysis of the intermediate sample (Figure 3g). HAADF and EDS compositional maps of a representative nanocrystal from this sample (Figure 3h) evidence that the two phases, CsPbCl_3_ and CsPbI_3_, coexist in the same nanocrystal, with CsPbCl_3_ mainly located at the corners, providing evidence of what is essentially a CsPbI_3_/CsPbCl_3_ core–shell nanocrystal heterostructure.

Large-area EDS maps (>100 particles) confirm a core–shell compositional distribution across the entire ensemble (Figure S13). Specifically, Cl^–^ maps reveal that the chloride-rich shell forms a continuous domain spanning both the corners and edges rather than forming patchy regions. The line-profile analysis reveals an anisotropic exchange, so that the halide substitution proceeds more rapidly at the corners than along the flat edges (Figure S14). Thus, halide exchange is expected to initiate at the corners of the cubes as at these sites the atoms have lower coordination number than those on the facets, hence they present a lower activation barrier for ion exchange.^27^ The structural and morphological stability of the intermediate CsPbI_3_/CsPbCl_3_ hollow nanocubes was monitored over time by TEM, XRD, optical absorption and PL (Figure S15). The nanocrystals underwent a phase degradation after 1–3 days, along with the formation of large nanowires of the nonemissive δ-CsPbI_3_ phase. We also assessed the thermal stability of these CsPbI_3_/CsPbCl_3_ heterostructures by heating a colloidal dispersion of them at 80 °C for 1 h (Figure S16). Also in this case the nanocrystals in the sample were mainly transformed into nanowires of the nonemissive δ-CsPbI_3_ phase.

While more analyses of this intermediate sample will be presented later, for now we discuss the fully Cl^–^ exchanged sample. Low magnification TEM analysis (Figure 3i) of this sample again did not indicate drastic shape changes, and EDS compositional maps of a group of nanocrystals (Figure 3l) confirmed a full I^–^ → Cl^–^ exchange. HAADF-STEM analysis (Figure 3j) evidenced that channels had developed in several nanocubes. In most cases the channels extended from the outer surface to the central void region. In Figure 3k the channel is aligned with the direction of the electron beam. The presence of these channels, which is more frequent in the final sample but can also be seen in some of the early exchange samples (Figure S17), is most likely related to local dismantling of the particles as a consequence of the large volume shrinkage (26%) in going from the CsPbI_3_ to the CsPbCl_3_ phase (Table S2). In principle, channels might also be the result of local particle degradation during the exchange reaction, due to etching caused by the chemical environment.^28^ A likely culprit could be oleylamine, which is needed, in addition to oleic acid, to solubilize the PbCl_2_ salt used for the halide exchange (Figure S18). However, we can safely rule out any role of the oleylamine in the formation of channels in the I^–^ to Cl^–^ exchange reaction, since it was also used in the I^–^ to Br^–^ exchange, and no internal channels or hollow morphologies were observed, neither in the intermediate nor in the fully Br^–^ exchanged samples (Figure 2).

We performed additional analyses on the intermediate exchange samples. An atomic resolution HAADF-STEM image of a hollow nanocube from a Cl/I = 0.4 exchange sample is shown in Figure 4a: it is evident that a typical nanocube contains planar defects (white arrows) that were not observed in the original sample. Additional STEM images highlighting these structural defects across different particles are provided in the SI (Figure S19). The particles are monocrystalline both before and after the reactions. Moreover, the heterostructures are epitaxial, following the same mechanism of transformation during the exchange reaction. These defects consist of CsI stacks of the all-inorganic Ruddlesden–Popper (RP) Cs_2_PbI_2_Cl_2_ or RP-like phases^29^ (see also Figure S20) and most likely represent a way for the nanocrystals to release strain locally. A high resolution HAADF-STEM image of another nanocube from the same sample, this time overlaid on a unit cell volume map extracted from geometric phase analysis (GPA, see details in the Experimental Section) is shown in Figure 4b. Here, the red overlay identifies regions with larger unit cell volume, which are expected to correlate with CsPbI_3_ or, more in general, with I-rich regions. This agrees with the results from EDS analysis of Figure 3h, which predominantly locates the CsPbCl_3_ phase at the corners. The copresence of the two phases in the nanocrystal is also clear from the associated FFT patterns (Figure 4c).

**Figure 4:**
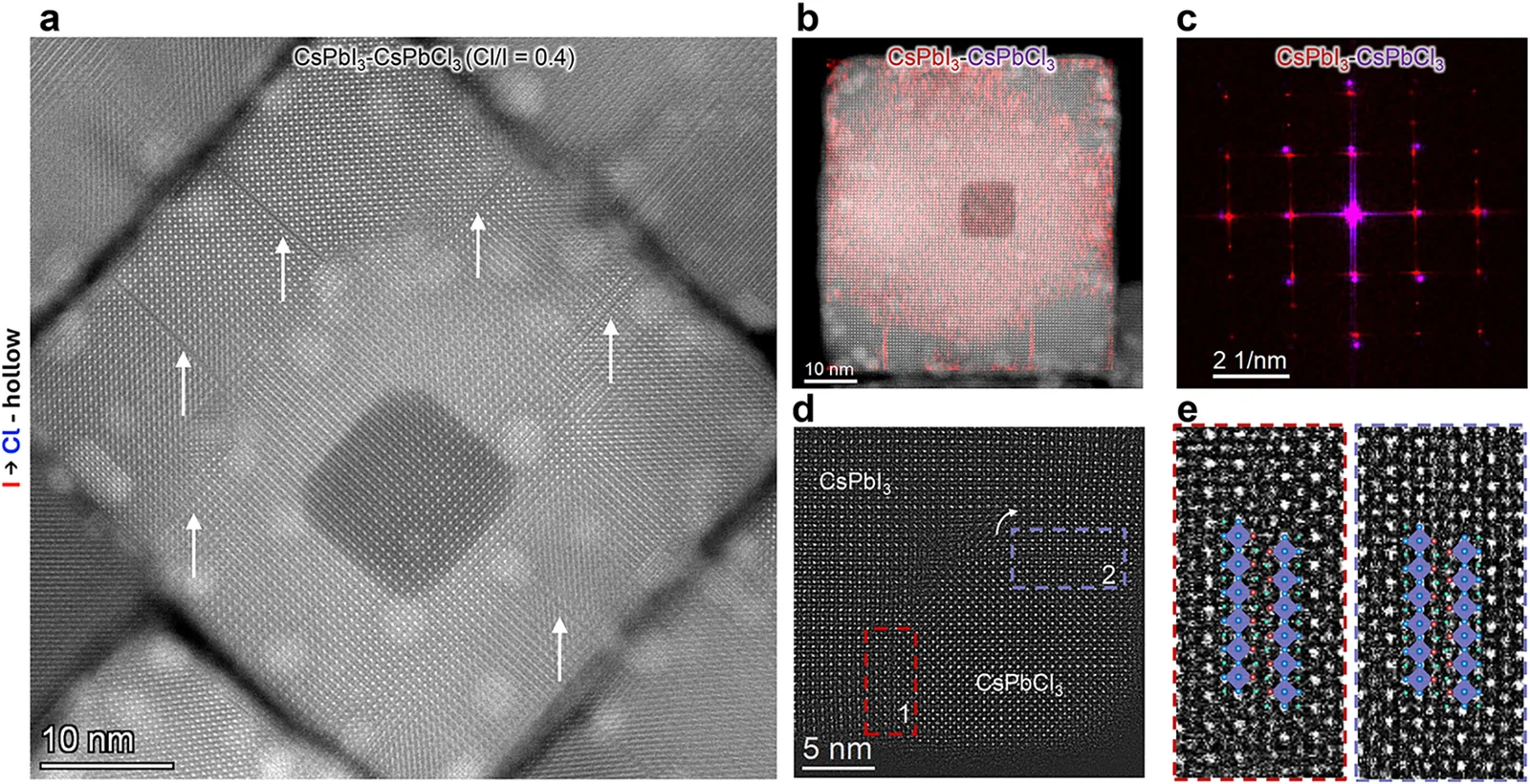
(a) Atomic resolution HAADF-STEM image of a hollow nanocube at an intermediate stage of exchange (Cl/I = 0.4). Ruddlesden–Popper (RP) type planar defects are indicated with white arrows. (b) An atomic resolution HAADF-STEM image of another hollow nanocube from the same sample, this time overlaid with a unit cell volume map extracted from GPA analysis (the CsPbI_3_ regions are marked in red) and (c) the corresponding FFT patterns extracted from the image. (d) HAADF-STEM image of a corner of another hollow nanocube from the same sample. The red and violet dashed regions marked as “1” and “2” highlight two orthogonal CsI epitaxial double layers. (e) Expanded views of regions 1 and 2 from panel (d) are shown here in (left) red dashed rectangle and (right) blue dashed rectangle (Cs^+^ = cyan, I^–^ = red, Cl^–^ = blue, [PbCl_6_]^4–^ octahedra = dark blue).

An atomic resolution HAADF-STEM image of a corner of a hollow nanocube from the same sample is reported in Figure 4d. The cubic CsPbCl_3_ domain in the [001] projection is easily identified due to the lower contrast of Cl^–^ atomic columns (caused by the low Z atomic number of Cl), which are almost invisible in the HAADF image. The regions dominated by orthorhombic CsPbI_3_ in the remaining parts of the image (especially the upper left region) display clearly visible I^–^ atomic columns, as I has a much higher atomic number than Cl. In regions marked as “1” and “2”, it is possible to identify double Cs–I atomic planes embedded in the CsPbCl_3_ domains near the interface with the CsPbI_3_ region. These CsI bilayers connected to the Pb–Cl planes of the perovskite are part of the unit cell plane-stacking sequence of the Cs_2_PbI_2_Cl_2_ RP phase, as discussed earlier. At the early stages of exchange, the location of these CsI bilayers appears to be near the boundaries of the exchanged regions, that is, at the edges/corners of the cubes, although their identification at these stages is more challenging, see for example Figure S17 taken from a Cl/I = 0.2 exchange sample. At later stages, the halide exchange front has overcome many of these Cs–I planes, so that the planes now cut across CsPbCl_3_ regions (Figure 4b). These planes might represent the preferred paths through which iodide ions diffuse from the inner regions (still iodide rich) to the outer surface of the nanocrystals, given their low diffusivity in CsPbCl_3_. As the exchange progresses, the double Cs–I atomic planes should be converted into Cs–Cl ones. Eventually, many of these planar defects are removed from the nanocrystals, as their presence is infrequent in the fully exchanged nanocrystals (Figure S21). We can speculate that the channels seen in the final exchange sample might have developed along some of these planes, although we have no direct evidence of it.

We also performed direct strain analysis by four-dimensional STEM (4D STEM) on a CsPbI_3_/CsPbCl_3_ hollow nanocube from the same sample (Cl/I = 0.4). Figure S22a presents a virtual bright field image and Figure S22b shows the representative local diffraction patterns acquired from the CsPbCl_3_ (green) and CsPbI_3_ (red) domains. The diffraction patterns confirmed that the CsPbCl_3_ domains adopt a cubic phase with the *Pm̅*3*m* space group, whereas the CsPbI_3_ domains adopt an orthorhombic phase with the *Pnma* space group (Table S2), indicating a phase transition from orthorhombic to cubic during I^–^ to Cl^–^ exchange. Consistent with the GPA results, analysis of change in lattice vectors based on 4D STEM (Figure S22c,d) reveals that the corner CsPbCl_3_ domains have a smaller lattice parameter than that of the inner CsPbI_3_ region, reflecting the lattice mismatch between CsPbI_3_ and CsPbCl_3_.

We then conducted Cl^–^ exchange on the regular CsPbI_3_ nanocubes under the same reaction conditions as in the previous experiments. Similarly to the hollow nanocubes, the absorption spectra evidenced a progressive Cl^–^ incorporation with increasing amounts of chloride ions added (Figure 5a). The trend in the PL spectra was similar to the hollow case, with the persistence of the CsPbI_3_ emission peak in the CsPbI_3_/CsPbCl_3_ intermediate exchange samples (Figure 5b). Here too, the distinct presence of optical features from both phases is indicative of a type-I band alignment.^16^ In the XRD patterns of the partially exchanged samples, peaks corresponding to both CsPbCl_3_ and CsPbI_3_ phases were identified, with the main peaks from CsPbCl_3_ again shifted to lower angles and broadened, especially at the early stages of exchange (Figure 5c,d). Low magnification TEM images of regular cubes at an intermediate CsPbI_3_/CsPbCl_3_ (Cl/I = 0.4) exchange stage (Figure 5e) sometimes revealed a roughening of the shape of the crystals. Similar to the hollow cubes, these nanocrystals also presented Cs–I planes and several channels on the surface (Figure 5f). Here too, halide exchange occurs initially at the corners of the cube, as supported by EDS analysis (Figure 5i) and leads to intermediate samples in which CsPbI_3_ and CsPbCl_3_ domains coexist.

**Figure 5:**
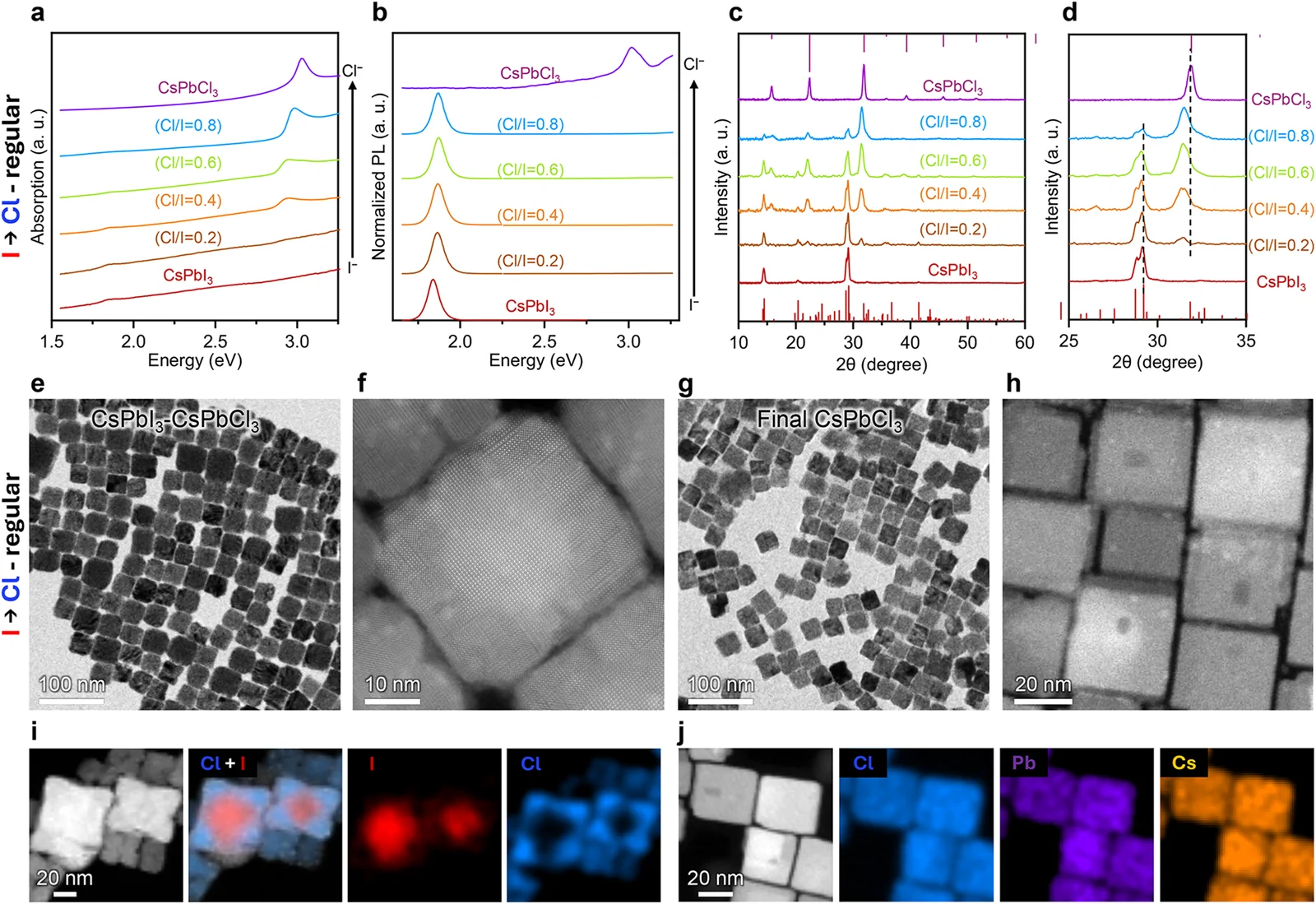
(a) Optical absorption spectra, (b) PL spectra and (c, d) XRD patterns and details of the 2Θ = 25°–35° range for the starting CsPbI_3_, intermediate CsPbI_3_/CsPbCl_3_ (Cl/I = 0.2, 0.4, 0.6, 0.8) and final CsPbCl_3_ regular nanocubes. (e) TEM image, (f) HAADF-STEM image and (i) STEM-EDS elemental maps of the CsPbI_3_/CsPbCl_3_ (Cl/I = 0.4) nanocubes. (g) TEM image, (h) HAADF-STEM image and (j) STEM-EDS elemental maps of the fully exchanged CsPbCl_3_ nanocubes.

Consistent core–shell architectures can be clearly discerned in the large-area EDS maps and line-profile analysis (Figures S23, S24) of several CsPbI_3_/CsPbCl_3_ regular cubes. They appear to exhibit size-dependent exchange kinetics, wherein smaller particles appear to be at a more advanced stage of exchange than the larger ones. As for hollow nanocubes, also in regular nanocubes the initial nucleation of chloride-rich domains at the corners is followed by rapid propagation to the edges, confirming a general corner-initiated pathway toward continuous core–shell heterostructures. Hence, also in this case we could prepare, by partial halide exchange, epitaxial CsPbI_3_/CsPbCl_3_ core–shell nanocrystal heterostructures. Additional HAADF-STEM images of individual nanocrystals at an earlier stage of exchange, reported in Figure S25, confirm the above trend, especially the exchange starting at the corners/edges and the formation of CsI bilayers. Finally, and similarly to the hollow nanocubes case discussed earlier, after 1 week these regular nanocube heterostructures were mostly transformed (Figure S26) into δ-CsPbI_3_ phase nanowires.

In the final sample (the one recovered after complete Cl^–^ exchange) a notable feature is the formation of a hollow region in many of the nanocrystals (Figure 5g,h,j). Atomic resolution HAADF-STEM images of representative individual nanocrystals from this final sample are reported in Figure 6. These images reveal a variety of structural features, often combined in individual nanocrystals, such as the formation of an inner cavity with no other apparent defects (Figure 6a); the presence of RP-like planar defects but no inner cavity (Figure 6b), or the coexistence of both a cavity and RP defects, in some cases with the additional presence of channels from the outer surface to the inner cavity (Figure 6c). The formation of a cavity can be again explained by considerable volume contraction as the halide exchange progresses. RP-like defects and channels may also concur to locally reduce strain during the exchange reaction, as for the hollow nanocubes discussed earlier. Additional low magnification TEM images of the initial sample, of various intermediate samples, and of the final sample are reported in Figure S27.

**Figure 6:**
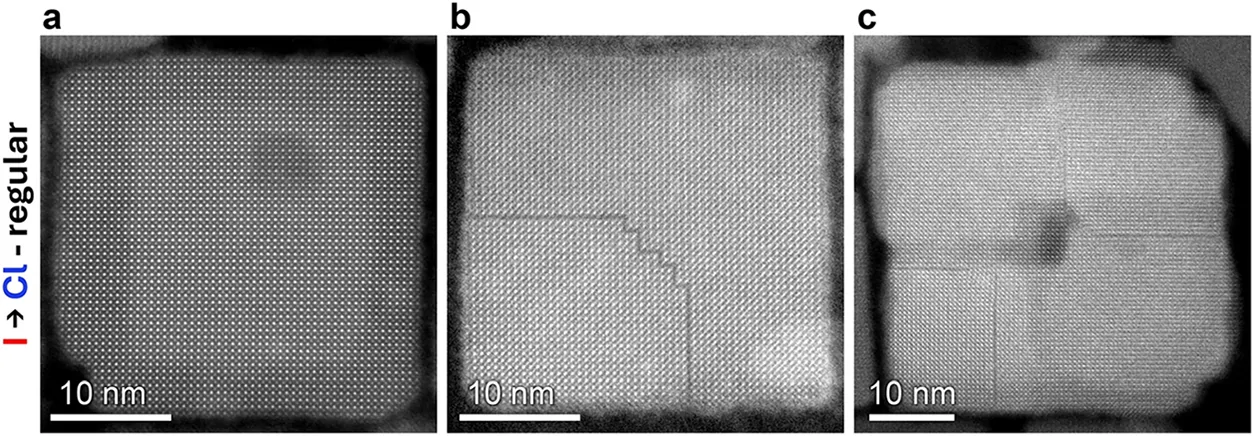
(a–c) Atomic resolution HAADF-STEM images of three representative nanocrystals from a sample of regular (initially) CsPbI_3_ nanocubes that had undergone complete I^–^ → Cl^–^ exchange. The three distinctive features are formation of an inner cavity (a), extensive RP-like planar defects (b), and the coexistence of the two, in some cases with the addition of channels from the outer surface to the inner cavity (c).

We performed finite-element calculations based on idealized continuum mechanical models under linear elastic theory to qualitatively map the distribution of hydrostatic strain during the I^–^ to Cl^–^ exchange for both regular and hollow morphologies (Figure S28a). In regular nanocubes (40 nm size), the Cl-rich regions initially form at the corners and subsequently expand along the edges.^27^ As this Cl^–^ shell progressively surrounds the I-based core, it intensifies the inward pressure, causing the core to become gradually more compressed. This cumulative effect leads to a state of maximum compression throughout the center of the nanocube (Figure S28b, left). Conversely, the hollow architecture, which features a 10 nm central void, provides an internal free surface that facilitates inward lattice relaxation. This relaxation prevents the I^–^ core from being significantly squeezed by the Cl^–^ shell. While the CsPbI_3_ regions between the void and the outer Cl-based shell are crushed by internal compression, the area immediately surrounding the hollow region remains more efficiently protected from these extreme compressive forces (Figure S28b, right). Interestingly, at the outer surface, both morphologies behave similarly (Figure S28c): here, the tensile strain arising from CsPbCl_3_ regions is the dominant factor, indicated by the localized red and orange regions appearing primarily at the CsPbCl_3_/CsPbI_3_ interface. By mitigating the internal compression that would otherwise develop in a regular cube, the hollow geometry then significantly reduces the mechanical stress, potentially rendering this architecture more robust against high-strain scenarios. In the case of regular nanocubes, this severe compression would create a substantial driving force. We hypothesize that this highly localized stress, potentially acting in concert with vacancy-mediated transport, contributes to the formation of the inner cavities that are observed in the fully exchanged samples (Figure 6).

We measured the PLQY and τ_avg_ of the CsPbI_3_/CsPbCl_3_ intermediates and final CsPbCl_3_ samples for both hollow nanocubes and regular nanocubes (Table 3). The fitting parameters of the PL decay spectra are reported in Table S5. For both series, partial Cl^–^ exchange led to a continuous decrease in PLQY and τ_avg_, following a trend similar to the I^–^ → Br^–^ results. The final, fully exchanged CsPbCl_3_ samples of both geometries, however, showed very low PLQY (∼1%), suggesting that nonradiative losses dominate at the ensemble level once the exchange is complete. Replacing iodide with chloride introduces a large 26% volume contraction (Table S2), which is expected to generate substantially more defects than a lower-strain iodide-bromide alloy system. As discussed before, in the hollow nanocubes the internal cavity allows the crystal lattice to relax inward (Figure S28), which may help prevent the I-rich core from being compressed as severely as in the regular nanocubes, where the lack of this internal relaxation pathway would cause the outer CsPbCl_3_ shell to squeeze the CsPbI_3_ core more tightly (Figure S28b). This difference in internal compression is a plausible qualitative rationale for why the PLQY of the regular nanocube heterostructures decreases somewhat more than that of the hollow ones over the same exchange range (Table 3). The intermediate exchange samples exhibit τ_avg_ of the order of hundreds of ns–values comparable to those observed in pure CsPbI_3_ and alloyed nanocubes (Tables 1, 2, and 3). Yet, according to the STEM-EDS elemental maps (Figure 5i) the homogeneous alloy formation can be safely ruled out, confirming instead the presence of a phase-segregated core–shell system. Given that fully exchanged CsPbCl_3_ exhibits much shorter τ_avg_ values (∼9–10 ns), the long ensemble decay times in the intermediate exchange samples are tentatively associated with emission from the CsPbI_3_ core.

**Table 3:** PLQY and τ_avg_ of Starting, Intermediate, and Final Samples in the I^–^ → Cl^–^ Exchange Reaction

**Sample**	**PLQY (%)**	**τ**_**avg**_ **(ns)**
**Hollow nanocubes**		
CsPbI_3_	42 ± 5	442
Cl/I = 0.2	29 ± 4	307
Cl/I = 0.4	23 ± 3	241
CsPbCl_3_	∼1	10
**Regular nanocubes**		
Start CsPbI_3_	45 ± 6	293
Cl/I = 0.2	20 ± 3	194
Cl/I = 0.4	14 ± 2	176
CsPbCl_3_	∼1	9

### Cl^–^ → I^–^ Exchange

We finally investigated the Cl^–^ → I^–^ exchange process for both hollow and regular nanocubes. For both samples, the reaction went through intermediate stages that were remarkably different from those of the I^–^ → Cl^–^ exchange discussed previously. In this case, it is easier to discuss the regular nanocubes first. Here, the initial stage of exchange was a surface reaction that transformed the outer CsPbCl_3_ surface into RP-like regions through the formation of several CsI bilayers (Figure 7a–c), thus creating a structure that could be roughly described as a CsPbCl_3_ core/RP-like shell. This behavior is very similar to previous findings from our group on the early stages of I^–^ exchange on much smaller (∼10 nm) CsPbCl_3_ nanocrystals, where a CsI bilayer was seen covering completely the entire surface of the nanocrystals.^16^ Optical data (Figure 7g,h) indicate the presence of the CsPbI_3_ phase as well from the early stages. Additional atomic resolution HAADF-STEM images of this intermediate sample are presented in Figure S29. A distinctive feature of the partially exchanged nanocubes is that the CsI bilayers were almost always confined at or near the facets, and only rarely they extended deep inside the nanocrystals (unlike the I^–^ → Cl^–^ exchange discussed earlier). Our density functional theory (DFT) calculations indicate that a configuration in which CsI bilayers are buried deep inside a CsPbCl_3_ nanocube is significantly more stable than configurations where the bilayers are situated on or near a facet (Figure S30a). However, despite this strong thermodynamic preference for the central regions (up to 84 kcal·mol^–1^ stabilization compared to the least stable surface configuration), the extremely low diffusivity of iodide ions within CsPbCl_3_ prevents the system from reaching this thermodynamic minimum, leaving the less stable configurations as the only kinetically accessible structures observed experimentally.

**Figure 7:**
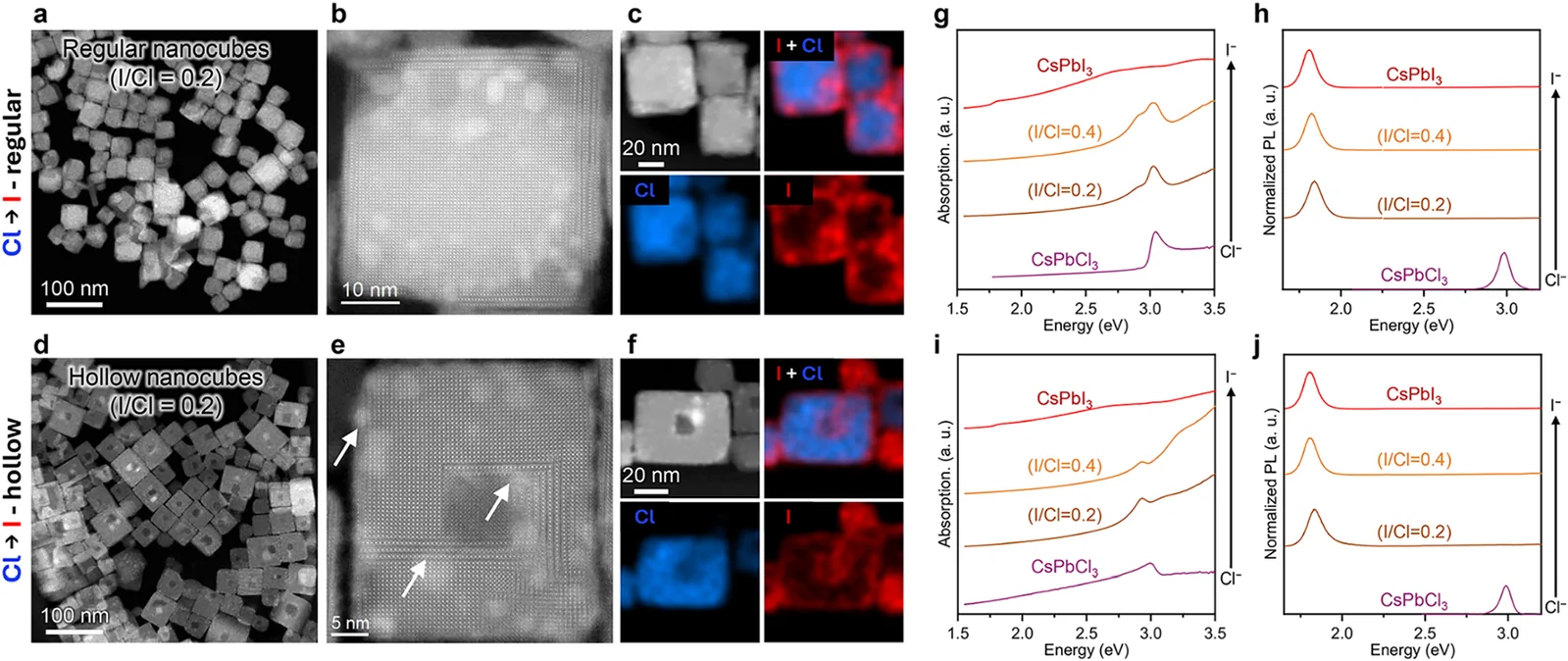
(a, b) HAADF-STEM images and (c) EDS elemental maps of intermediate exchange (I/Cl = 0.2) regular nanocubes. (d, e) HAADF-STEM images and (f) EDS elemental maps for intermediate exchange (I/Cl = 0.2) hollow nanocubes. The CsI bilayers surrounding the outer and inner surface and penetrating the shell connecting the inner cavity with the outer surface are indicated with white arrows in panel e. (g, i) Absorption and (h, j) PL spectra of CsPbCl_3_, CsPbI_3_/CsPbCl_3_ (I/Cl = 0.2 and 0.4) and CsPbI_3_ (g, h) regular nanocubes and (i, j) hollow nanocubes.

The same exchange reaction on the hollow nanocubes (Figure 7d–f) produced a significantly different outcome, as in this case CsI bilayers were found on both the outer and the inner surface of the cubes and also cutting across the nanocrystals in a way that connected the outer and the inner surface. While we show several examples in Figure S31, the nanocrystal of Figure 7e is particularly telling. Here, the lower right corner has been transformed to CsPbI_3_. At the same time, several CsI bilayers are seen across the nanocrystal, with many surrounding the inner facets, and several CsI bilayers (on the left) connecting the inner cavity with the outer surface. We attribute this higher variability in the CsI bilayer texturing to the distinct morphology of the hollow nanocubes. The presence of the internal cavity is expected to provide greater structural flexibility compared to a rigid solid core (in regular nanocubes), which likely promotes higher diffusivity of the iodide ions. Thus, by mitigating the kinetic limitations observed in regular nanocubes, the hollow morphology enables the CsI bilayers to access their thermodynamic minimum. Our DFT calculations for both small and large internal voids (Figure S30b) confirm that the thermodynamic driving force pushing the CsI bilayers inward remains fully valid across all morphologies. Interestingly, the magnitude of this stabilization provides direct evidence of the cavity’s mechanical buffering: for comparable configurations in different morphologies, we obtained −66 kcal·mol^–1^ stabilization (relative to the least stable structure) in regular nanocubes, −45 and – 42 kcal·mol^–1^ in hollow nanocubes with small and large voids, respectively. These values point to the internal voids partially relieving the global lattice strain, consequently softening the thermodynamic penalty of having these CsI layers at the surface.

In both regular and hollow nanocubes a signature of the formation of these CsI bilayers was found in the XRD patterns of the intermediate exchange samples, where features that could be assigned to the Cs_2_PbI_2_Cl_2_ RP phase were identified (Figure S32). Here too, in the intermediate exchange samples the emission from CsPbCl_3_ was quenched due to efficient exciton transfer from the CsPbCl_3_ regions to the RP and/or CsPbI_3_ regions, and only emission from CsPbI_3_ could be observed. Finally, complete exchange led in both cases to nanocrystals that were fully CsPbI_3_, according to optical spectra (Figure 7g–j) and XRD (Figure S32). However, in both cases their shapes were very irregular, and the nanocrystals were highly unstable, as they rapidly transformed into long wires of the δ-CsPbI_3_ phase (Figures S33 and S34).

The striking difference between the I^–^ → Cl^–^ and Cl^–^ → I^–^ exchanges on nanocrystals is likely explained by two separate and likely concurring factors: (i) the disparate mechanical properties of the two starting lattices (Table S6), with the CsPbCl_3_ being significantly stiffer than CsPbI_3_, such that it cannot elastically accommodate the 26% volume expansion when incorporating I^–^ into the lattice, as the expanding CsPbCl_3_ core is physically blocked by the outer CsPbI_3_ shell. Consequently, in CsPbCl_3_ this strain is partially released through the formation of the observed CsI bilayers. This occurs also in CsPbI_3_ but to a much lower extent; (ii) the small Cl^–^ ions should have much higher diffusivity in CsPbI_3_ than the much larger I^–^ ions in CsPbCl_3_, so again in CsPbCl_3_ the only way for I^–^ ions to engage in exchange reactions is through the pervasive formation of CsI bilayers.

### Carrier Dynamics of Core–Shell CsPbI_3_/CsPbCl_3_ Nanocubes

The core–shell CsPbI_3_/CsPbCl_3_ heterostructures based on both hollow and regular nanocubes represent the most compelling case of core–shell nanocrystals entirely based on halide perovskites synthesized to date. Unlike previously reported alloyed or partially segregated systems, our CsPbI_3_/CsPbCl_3_ heterostructures exhibit two distinct perovskite domains connected through epitaxial interfaces within a single nanocrystal. This unique architecture provides a platform for further investigating interfacial optical coupling and charge-transfer processes in the perovskite heterostructures. The combination of semiconductors CsPbCl_3_ and CsPbI_3_ with different bandgaps within the same nanocrystal is expected to result in the transfer of photoexcited excitons and tunable dynamics as compared to the pure compounds.

To study the exciton dynamics in the CsPbI_3_/CsPbCl_3_ heterostructures, we performed transient absorption (TA) spectroscopy on the freshly prepared colloidal dispersions of hollow and regular nanocubes at three stages: starting CsPbI_3_, partially exchanged CsPbI_3_/CsPbCl_3_ (Cl/I = 0.4) and fully exchanged CsPbCl_3_ samples. A pump wavelength of 390 nm was used to excite both CsPbCl_3_ and CsPbI_3_ domains in the nanocubes and compare them with the pure compositions of the starting CsPbI_3_ and fully exchanged CsPbCl_3_ nanocubes. As exciton transfer from the CsPbCl_3_ to the CsPbI_3_ regions of the heterostructures was expected due to the different bandgaps, the analysis of TA spectra focused on the photoinduced transmission (ground-state bleach, GSB) spectra and dynamics, originating from the excitons in CsPbCl_3_ and CsPbI_3_. For hollow nanocubes, the most interesting sample was the Cl/I = 0.4 one, where the excitonic peaks of both CsPbCl_3_ and CsPbI_3_ components were observable in the linear absorption spectra (Figure S35a) and which gave a measurable TA signal from both CsPbCl_3_ and CsPbI_3_ components, albeit only at a relatively high pump excitation fluence (see Experimental Section for details). Samples of hollow nanocubes with a lower Cl/I ratio of 0.2 were also studied but did not show signatures of the CsPbCl_3_ phase, possibly due to its relatively small volume fraction (Figure S35b). In the following, we will continue the discussion only for the case of hollow nanocubes, as the observed dynamics were similar in regular nanocubes, for which we direct the reader to the SI (Figure S36).

The steady-state absorption spectrum of the Cl/I = 0.4 hollow nanocube heterostructures evidenced two excitonic features at 430 and 670 nm, corresponding to the CsPbCl_3_ and CsPbI_3_ domains, respectively (Figure S35a). In comparison, the starting CsPbI_3_ and final CsPbCl_3_ samples showed only band edge excitonic features at around 675 and 410 nm, respectively (Figure S35a). The weak GSB of the CsPbCl_3_ component of the heterostructure appeared at around 430 nm, red-shifted in comparison to pure CsPbCl_3_ (Figure 8a), possibly due to strain and/or alloying. The GSB of CsPbCl_3_ decayed faster in the CsPbI_3_/CsPbCl_3_ heterostructure than in the pure CsPbCl_3_ hollow nanocubes (Figure 8b). A biexponential function was sufficient to fit the observed decays, yielding the amplitude-weighted times of 45 ± 20 ps in the heterostructure and 240 ± 30 ps in pure CsPbCl_3_ (Table S7). That accelerated decay of the CsPbCl_3_ GSB is correlated with a few ps delay in reaching the maximum of the GSB signal of the CsPbI_3_ phase in the heterostructure relative to the pure CsPbI_3_ sample (Figure 8c, see Table S8 for details). These two connected observations are consistent with the exciton transfer from the CsPbCl_3_ shell (faster GSB decay) to the CsPbI_3_ core (a delayed GSB maximum), in agreement with a prior report.^17^ In addition, both the starting CsPbI_3_ sample and the intermediate sample exhibited a weak and broad, slowly decaying bleach centered near 450 nm (Figures 8a and S35b), which was assigned to a higher-energy transition of the CsPbI_3_ domains. As this spectral feature overlaps with GSB from CsPbCl_3_, it may contribute to the longer component of the biexponential decay of the GSB, and is also consistent with electronic coupling between CsPbCl_3_ and CsPbI_3_ components of the heterostructure.

**Figure 8:**
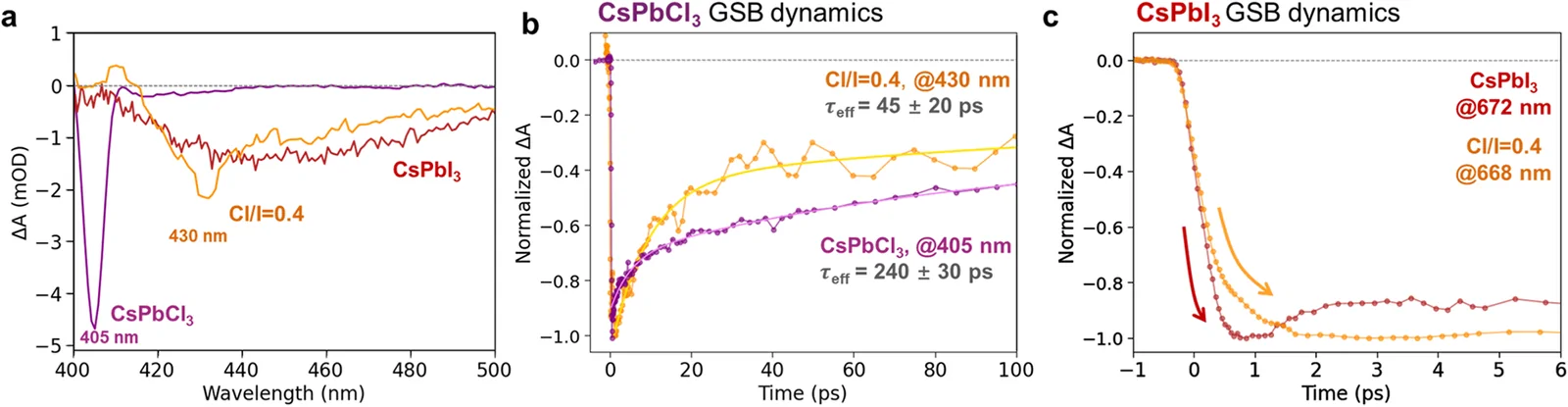
(a) TA spectra at 5 ps pump–probe delay in the 400–500 nm spectral range of CsPbCl_3_, CsPbI_3_/CsPbCl_3_ (Cl/I = 0.4) and CsPbI_3_ hollow nanocubes. (b) Comparison of the dynamics of the GSB of CsPbCl_3_ component in CsPbI_3_/CsPbCl_3_ (Cl/I = 0.4) and CsPbCl_3_ hollow nanocubes. (c) Comparison of the dynamics of the GSB of the CsPbI_3_ component in CsPbI_3_/CsPbCl_3_ (Cl/I = 0.4) and CsPbI_3_ hollow nanocubes.

## Conclusions

In summary, we have studied a set of halide exchange reactions in large halide perovskite nanocrystals with either regular nanocube or hollow nanocube geometries. The exchanges between the perovskites with the closest lattice parameters (I^–^ → Br^–^ and Br^–^ → Cl^–^) proceed with a gradual change in lattice parameter and intermediate optical properties, while the dynamics of the I^–^ ↔ Cl^–^ replacement are more complex. Moreover, for the I^–^ → Cl^–^ exchange and the reverse case we could observe different behaviors for the regular and hollow configurations. Our results indicate that the initial presence of a hollow region in the CsPbI_3_ nanocrystals might help to alleviate the strain generated during the exchange process. Also, the formation of CsI bilayers is a mechanism through which strain can be partially relieved during the I^–^ → Cl^–^ exchange and especially in the reverse case. Additionally, these bilayers might represent preferred avenues for exchange between Cl^–^ and I^–^ from the inner regions of the nanocrystals, since I^–^ escape through the CsPbCl_3_ shell appears to be less likely. While the presence of the hollow region can help to release the strain in CsPbI_3_/CsPbCl_3_ heterostructures, the substantial volume difference between CsPbI_3_ and CsPbCl_3_ always produces many lattice defects, which negatively affect the PLQY and structural stability. The intermediate core–shell CsPbI_3_/CsPbCl_3_ heterostructures were stable enough to be studied spectroscopically, and in both cases we observed dynamics consistent with electronic coupling and picosecond-scale exciton transfer from the CsPbCl_3_ shell to the CsPbI_3_ core region. Future strategies employing concomitant A- and B-site cation alloying might represent a promising route to mitigate lattice strain and stabilize the core–shell architectures by creating buffer regions that might also prevent or at least delay halide interdiffusion.

## Experimental Section

### Chemicals

Hexadecane (99%), oleic acid (OA, technical grade, 90%), oleylamine (OLAm, 98%), cesium­(I) carbonate (Cs_2_CO_3_, 98%), silver acetate (AgOAc, 99.9%), lead­(II) acetate trihydrate (Pb­(OAc)_2_·3H_2_O, 99.99%), lead­(II) chloride (PbCl_2_, 99.999%), lead­(II) bromide (PbBr_2_, 99.999%), lead­(II) iodide (PbI_2_, 99.999%), dihexylamine (97%), 1-octadecene (ODE, 90%) and toluene (anhydrous, 99.8%) were purchased from Sigma-Aldrich. Benzoyl bromide (98%) was purchased from Tokyo Chemical Industry (TCI). All reagents were used as received without any further experimental purification.

### Preparation of the Samples and Exchange Reactions

A general scheme of preparation of the samples and of the various exchange reactions is presented in Scheme 1.

**Scheme 1:**
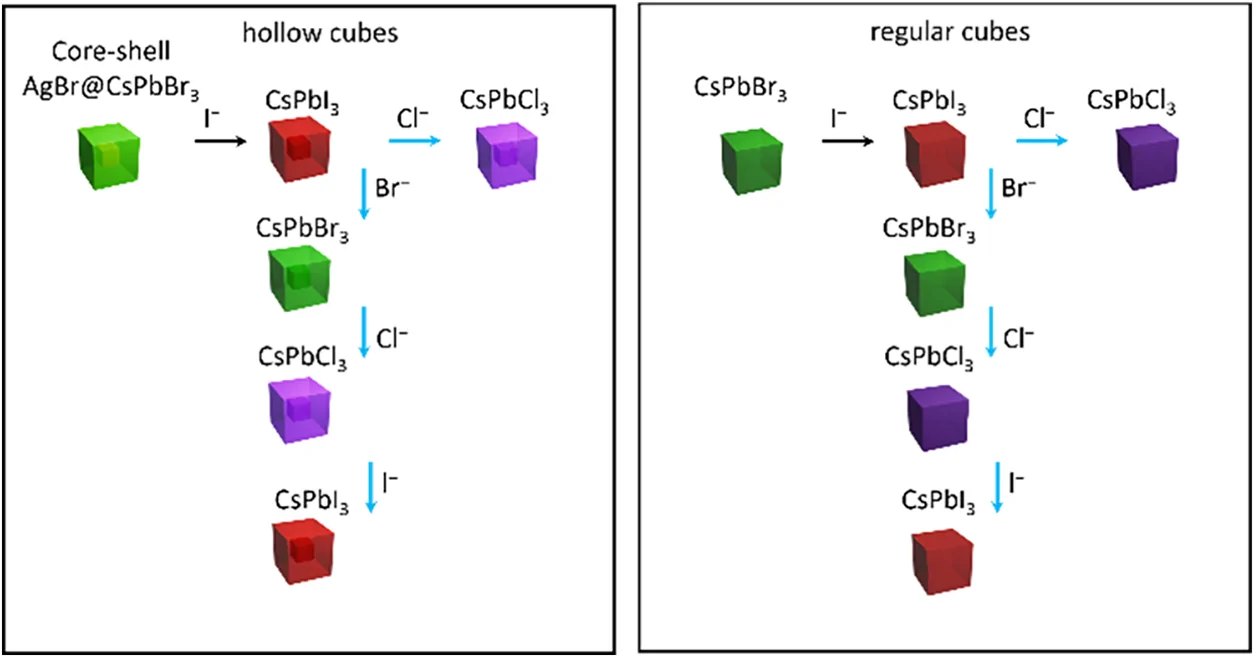
Preparation of the Samples and Corresponding Halide Exchange Reactions^a^

a In the scheme, all the cyan arrows represent halide exchange reactions studied in this work.

In essence, the progenitor for the hollow nanocubes was a sample of core@shell AgBr@CsPbBr_3_ cubes, prepared by direct synthesis. This was then converted to hollow CsPbI_3_ cubes, from which hollow CsPbBr_3_ and CsPbCl_3_ cubes were obtained either by sequential exchange (I^–^ → Br^–^ → Cl^–^) or direct I^–^ → Cl^–^ exchange. The progenitor for the regular nanocubes was a sample of regular CsPbBr_3_ cubes, prepared by direct synthesis. This was then converted to regular CsPbI_3_ cubes, from which regular CsPbBr_3_ and CsPbCl_3_ cubes were obtained either by sequential exchange (I^–^ → Br^–^ → Cl^–^) or direct I^–^ → Cl^–^ exchange.

#### Synthesis of CsPbI_3_ Hollow Nanocubes (Scheme 1, Left)

The synthesis of CsPbI_3_ hollow nanocubes was carried out through I^–^ exchange on the core@shell AgBr@CsPbBr_3_ cubes based on previous research.^26^ First, core@shell AgBr@CsPbBr_3_ nanocubes were synthesized by partial replacement of Pb^2+^ by Ag^+^ in the synthesis of CsPbBr_3_ nanocrystals. The I^–^ exchange reaction was conducted under ambient temperature in the glovebox. 2 mL of AgBr@CsPbBr_3_ suspension (15 mM in Br^–^) was dispersed in 4 mL of toluene, then 1 mL of iodine ion precursor (50 mM in I^–^) solution was added under vigorous stirring. Complete I^–^ exchange was confirmed by the photoluminescence measurements. The crude solution was precipitated upon centrifugation at 6000 rpm for 10 min, and the precipitate was redispersed in 2 mL of toluene for further characterizations.

#### Synthesis of Regular CsPbI_3_ Nanocubes (Scheme 1, Right)

The synthesis of regular CsPbI_3_ nanocubes was carried out through I^–^ exchange on the large-sized CsPbBr_3_ nanocubes.

##### Synthesis of Regular CsPbBr_3_ Nanocubes (∼40 nm)

0.05 mmol of Cs_2_CO_3_ and 0.2 mmol of Pb­(OAc)_2_·3H_2_O were weighed in a flask, then 1.5 mL (4.73 mmol) of oleic acid, 0.56 mL (2.40 mmol) of dihexylamine and 12 mL of ODE were added using the pipet. The flask was connected to a Schlenk line and was pumped to vacuum at room temperature for 30 min and at 100 °C for 60 min. The flask was then fluxed under a nitrogen atmosphere, and the temperature was increased to 190 °C. 550 μL of benzoyl bromide solution (50 μL of benzoyl bromide (0.42 mmol) dissolved in 500 μL of 1-octadecene) was swiftly injected. After 4 min, the reaction was quenched rapidly to room temperature using an ice–water bath. The crude solution was precipitated with centrifugation at 6000 rpm for 10 min. The supernatant was discarded and the precipitate was dispersed in 4 mL of anhydrous toluene. The resulting dispersion was centrifuged at 2000 rpm for 5 min, leading to a yellowish precipitate on the bottom of the vial, then 4 mL of anhydrous toluene was poured to get the solution of CsPbBr_3_ nanocrystals.

##### Synthesis of Regular CsPbI_3_ Nanocubes through I^–^ Exchange

The I^–^ exchange reaction was conducted under ambient temperature in the glovebox. 2 mL of CsPbBr_3_ suspension (15 mM in Br^–^) was dispersed in 4 mL of toluene, then 1 mL of iodine precursor (50 mM in I^–^) solution was added under vigorous stirring. Complete I^–^ exchange was confirmed by the photoluminescence measurements. The crude solution was precipitated upon centrifugation at 6000 rpm for 10 min, and the precipitate was redispersed in 2 mL of toluene for further characterizations.

### Preparation of I^–^ (Br^–^ and Cl^–^) Precursor Solutions

The iodine ion precursor solution was prepared by loading 0.5 mmol of PbI_2_, 2.5 mL of OA, 2.5 mL of OLAm and 15 mL hexadecane into a 40 mL vial and placing it into an aluminum block on top of a hot plate. The mixture was pumped to vacuum at room temperature for 30 min and at 100 °C for 30 min. Then the temperature increased to 150 °C until the lead halide salt was dissolved (∼15–20 min). The final mixture was cooled down to room temperature and stored in a nitrogen-filled glovebox. The Br^–^ and Cl^–^ precursor solutions were prepared by the same procedure by loading 0.5 mmol of PbBr_2_ and PbCl_2_ in the vial.

### I^–^ to Br^–^ Exchange Reactions

All exchanges were carried out at room temperature. One mL of CsPbI_3_ nanocubes solution (15 mM in I^–^) was dispersed in 2 mL of toluene. 60 μL of bromide precursor solution (50 mM in Br^–^, corresponding to a Br/I ratio equal to 0.2) was injected to it under vigorous stirring, after which the exchange reaction was allowed to proceed for 10 min, to yield CsPb­(I:Br)_3_ nanocubes at an early stage of exchange. This step (injection of 60 μL of bromide precursor solution, followed by 10 min reaction) could be repeated several times, to yield CsPb­(I:Br)_3_ nanocubes at more advanced stages of exchange (corresponding to Br/I ratios equal to 0.4, 0.6, and 0.8). In a final addition step, an excess amount of bromide precursor solution (120 μL) was injected to ensure complete exchange and thus deliver CsPbBr_3_ nanocubes, as this addition led to a total Br/I ratio equal to 1.2:1. The nanocrystals, either at an intermediate stage or at the final stage of exchange, were precipitated from the crude solution by centrifugation at 6000 rpm for 5 min. The precipitate was dispersed in 1 mL of anhydrous toluene and stored in a nitrogen-filled glovebox for further characterizations.

### Br^–^ to Cl^–^ Exchange Reactions

All exchanges were carried out at room temperature. One mL of CsPbBr_3_ nanocubes solution (15 mM in Br^–^) was dispersed in 2 mL of toluene. 60 μL of chloride precursor solution (50 mM in Cl^–^, corresponding to a Cl/Br ratio equal to 0.2) was injected to it under vigorous stirring, after which the exchange reaction was allowed to proceed for 10 min, to yield CsPb­(Br:Cl)_3_ nanocubes at an early stage of exchange. This step (injection of 60 μL of chloride precursor solution, followed by 10 min reaction) could be repeated several times, to yield CsPb­(Br:Cl)_3_ nanocubes at more advanced stages of exchange (corresponding to Cl/Br ratios equal to 0.4, 0.6, and 0.8). In the final addition step, an excess amount of chloride precursor solution ( 120 μL) was injected to ensure complete exchange and thus deliver CsPbCl_3_ nanocubes, as this addition led to a total Cl/Br ratio equal to 1.2:1. The nanocrystals, either at an intermediate stage or at the final stage of exchange, were precipitated from the crude solution by centrifugation at 6000 rpm for 5 min. The precipitate was dispersed in 1 mL of anhydrous toluene and stored in a nitrogen-filled glovebox for further characterizations.

### I^–^ to Cl^–^ Exchange Reactions

All exchanges were carried out at room temperature. One mL of CsPbI_3_ nanocubes solution (15 mM in I^–^) was dispersed in 2 mL of toluene. 60 μL of chloride precursor solution (50 mM in Cl^–^, corresponding to a Cl/I ratio equal to 0.2) was injected to it under vigorous stirring, after which the exchange reaction was allowed to proceed for 10 min, to yield CsPbI_3_/CsPbCl_3_ nanocubes at an early stage of exchange. This step (injection of 60 μL of chloride precursor solution, followed by 10 min reaction) could be repeated several times, to yield CsPbI_3_/CsPbCl_3_ nanocubes at more advanced stages of exchange (corresponding to Cl/I ratios equal to 0.4, 0.6, and 0.8). In the final addition step, an excess amount of chloride precursor solution (120 μL) was injected to ensure complete exchange and thus deliver CsPbCl_3_ nanocubes, as this addition led to a total Cl/I ratio equal to 1.2:1. The nanocrystals, either at an intermediate stage or at the final stage of exchange, were precipitated from the crude solution by centrifugation at 6000 rpm for 5 min. The precipitate was dispersed in 1 mL of anhydrous toluene and stored in a nitrogen-filled glovebox for further characterizations.

### Cl^–^ to I^–^ Exchange Reactions

All exchanges were carried out at room temperature. One mL of CsPbCl_3_ nanocubes solution (15 mM in Cl^–^) was dispersed in 2 mL of toluene. 60 μL of iodide precursor solution (50 mM in I^–^, corresponding to an I/Cl ratio equal to 0.2) was added to it under vigorous stirring, after which the exchange reaction was allowed to proceed for 10 min, to yield nanocubes at an early stage of exchange. This step (injection of 60 μL of iodide precursor solution, followed by 10 min reaction) could be repeated several times, to yield nanocubes at more advanced stages of exchange (corresponding to I/Cl ratios equal to 0.4, 0.6, and 0.8). In the final addition step, an excess amount of iodide precursor solution (120 μL) was injected to ensure complete exchange and thus deliver CsPbI_3_ nanocubes, as this addition led to a total I/Cl ratio equal to 1.2:1. The nanocrystals, either at an intermediate stage or at the final stage of exchange, were precipitated from the crude solution by centrifugation at 6000 rpm for 5 min. The precipitate was dispersed in 1 mL of anhydrous toluene and stored in a nitrogen-filled glovebox for further characterizations.

### Characterizations

#### Optical Characterizations

Optical absorption spectra were collected on a Varian Cary 300 UV–vis absorption spectrophotometer. The PL spectra of perovskite nanocrystals were measured on a Varian Cary Eclipse spectrophotometer using an excitation wavelength of 350 nm for the CsPbBr_3_ and CsPbCl_3_ phases and of 400 nm for CsPbI_3_ phase. The suspensions were prepared by diluting nanocube samples in 3 mL of toluene in 1 cm path length quartz cuvettes. The PLQY was measured on the Edinburgh Instruments FLS920 spectrofluorometer equipped with an integrating sphere. The nanocube suspension was dispersed in anhydrous toluene with an optical density of 0.12 at 400 nm, which was the excitation wavelength employed for PLQY measurements, then PLQY was measured by using a calibrated integrating sphere. The PL decay measurements were conducted on the Edinburgh FLS900 fluorescence spectrometer equipped with a Xe lamp and a monochromator for steady-state PL excitation and a time-correlated single photon counting unit coupled with an Edinburgh Instruments EPL-375 pulsed laser diode (λ_ex_ = 371.8 nm, pulse width = 68.8 ps) for time-resolved PL.

TA experiments were performed on two different setups for different samples, keeping the same experimental conditions. The reported dynamics were acquired with relatively high pump fluence of 100 μJ/cm^2^, due to the weak signal from the Cl-component. Hollow CsPbI_3_ and heterostructures with Cl/I = 0.2 were measured at Lund University using a home-built setup seeded by 60 fs pulses at 796 nm generated by a Solstice amplifier (8 W, 4 kHz, Spectra Physics). The beam is split and directed to optical parametric amplifiers (TOPAS-C, Light Conversion) in order to generate 390 nm pulses for the pump, and 1350 nm pulses for the probe beam. The pump–probe delay is set by a mechanical delay stage, after which the pump beam is chopped to 2 kHz. The probe beam is focused in a CaF_2_ crystal to generate a supercontinuum pulse. After interacting with the sample, the probe beam enters a spectrograph to be dispersed and detected by photodiode arrays (Pascher Instruments). The heterostructures with Cl/I = 0.4 were measured at IIT Genova using a Yb-based femtosecond laser system (Pharos-SP-HP, Light Conversion) coupled to a tunable optical parametric amplifier (Orpheus, Light Conversion). The pump pulse duration was approximately 160 fs, while the instrument response function, experimentally determined from the pump–probe cross-correlation, was approximately 230 fs. TA spectra were acquired using a Harpia-TA spectrometer (Light Conversion). The supercontinuum white-light probe was generated by focusing a fraction of either the fundamental laser output at approximately 1030 nm or its second harmonic at approximately 515 nm into a sapphire crystal, yielding visible-near-infrared probe light in the 500–900 nm range (for probing GSB of CsPbI_3_ exciton) or UV-visible probe light in the 350–500 nm range (for probing CsPbCl_3_ exciton), respectively. We note that, in the present setup, no single broadband probe is available that can simultaneously cover the GSB features of both CsPbCl_3_ and CsPbI_3_. The pump–probe delay was controlled by varying the optical path length of the probe beam using a delay line. The measurements at IIT Genova were performed at a repetition rate of 50 kHz.

#### X-ray Powder Diffraction

XRD analysis was performed on a PANanalytical Empyrean X-ray diffractometer, endowed with a 1.8 kW Cu Kα ceramic X-ray tube (λ = 1.5406 Å) and a PIXcel3D 2 × 2 area detector, running at 45 kV and 40 mA. Perovskite nanocrystal solutions were concentrated in the vial through a flow of nitrogen, then were drop-cast on a zero-diffraction single crystal silicon substrate. The XRD patterns were collected under ambient conditions.

#### Transmission Electron Microscopy (TEM) and Scanning TEM (STEM)

TEM images in a large field of view were collected on a JEOL JEM-1400 Plus microscope with a thermionic gun (LaB_6_ crystal) with an acceleration voltage of 120 kV. Perovskite nanocrystal solutions were diluted and sonicated in anhydrous toluene and then dropped cast on the copper TEM grids with an ultrathin carbon film. High-resolution scanning transmission electron microscopy (HR-STEM) images were acquired on a probe-corrected Thermo Fisher Spectra 300 STEM operated at 300 kV. Images were acquired on a High-Angle Annular Dark Field (HAADF) detector with a current of 30 pA. Compositional maps were acquired using Velox, with a probe current of ∼150 pA and rapid rastered scanning by Energy-Dispersive X-ray spectroscopy (EDS) on a Dual-X system comprising two detectors, on either side of the sample, for a total acquisition angle of 1.76 sr. Geometric Phase Analysis (GPA) of the HR-STEM images was performed using the commercial Digital Micrograph software package and homemade scripts.^31–33^

#### Computational Methods

Finite-element analysis of the internal mechanical response was performed utilizing the open-source FEniCS computing platform (DOLFIN backend). 3D conformal tetrahedral meshes of the regular and hollow core–shell geometries were generated via the OpenCASCADE kernel with Gmsh and subsequently processed using meshio. The structural distortion was modeled within a 3D linear elastic continuum framework. Complete mathematical formulations, boundary conditions, and material-specific parameters employed for the modeling are detailed in section S1 of the SI. DFT calculations were performed with the CP2K software package utilizing the Gaussian and Plane Waves (GPW) method. Exchange and correlation interactions were described using the PBE functional. Valence electron wave functions were expanded using DZVP-MOLOPT-SR-GTH basis sets, while core electrons were represented by GTH norm-conserving pseudopotentials. The electron density was mapped onto a regular grid with a planewave cutoff of 400 Ry and four multigrids. The systems were modeled in the gas phase using nonperiodic boundary conditions within a 60 Å × 60 Å × 60 Å cubic simulation box, providing sufficient vacuum padding to avoid spurious interactions. Self-consistent field (SCF) iterations employed orbital transformation (OT) with DIIS acceleration (7 vectors), a convergence threshold of 1.0 × 10^–6^, and a maximum of 200 SCF cycles. Geometry optimizations were driven by the LBFGS algorithm for a maximum of 500 iterations. Relative stabilization energies were evaluated by calculating the difference in the total optimized ground-state energy of each configuration with respect to the least stable, surface-confined reference structure.

## Supplementary Material



## Notes

Schematic models on CsPbI_3_/CsPbCl_3_ heterostructures in regular and hollow nanocubes, and detailed calculational methods; a free of charge and open-source code to perform the strain calculations can be found on the GitHub repository: https://github.com/jllusarc/nanocube-strain-models; additional characterizations (TEM, XRD, PL decay and STEM analysis) of nanocubes in regular or hollow morphologies (PDF)
