# Supplementary material for: Halide Exchange Reactions in Large Halide Perovskite Nanocrystals
Source: ACS Nano. 2026 Oct 29;20(44):29667–81. doi: 10.1021/acsnano.6c10843 (PMC13757948; doi:10.1021/acsnano.6c10843)
Supplement: Supplementary file 1 [file nn6c10843_si_001.pdf]

Supporting Information for:

Halide Exchange Reactions in Large Halide Perovskite Nanocrystals

Zhanzhao Li[1,2‡*], Yurii P. Ivanov[3‡], Nikolaos Livakas[1], Lorenzo Tallarini[4], Jordi Llusar[1], Aswin Asaithambi[1], Andrea Toma[5], Dmitry Baranov[4], Giorgio Divitini[3], Liberato Manna[1*]

[1] Nanochemistry, Istituto Italiano di Tecnologia, Via Morego 30, 16163, Genova, Italy
[2] State Key Laboratory of Coordination Chemistry, School of Chemistry, Nanjing University, 210023, China
[3] Electron Spectroscopy and Nanoscopy, Istituto Italiano di Tecnologia, Via Morego 30, 16163, Genova, Italy
[4] Division of Chemical Physics and NanoLund, Department of Chemistry, Lund University, P.O. Box 124, Lund, SE-22100 Sweden
[5] Clean Room Facility, Istituto Italiano di Tecnologia, Via Morego 30, 16163, Genova, Italy

Contents: Figures S1-S36, Tables S1-S8.

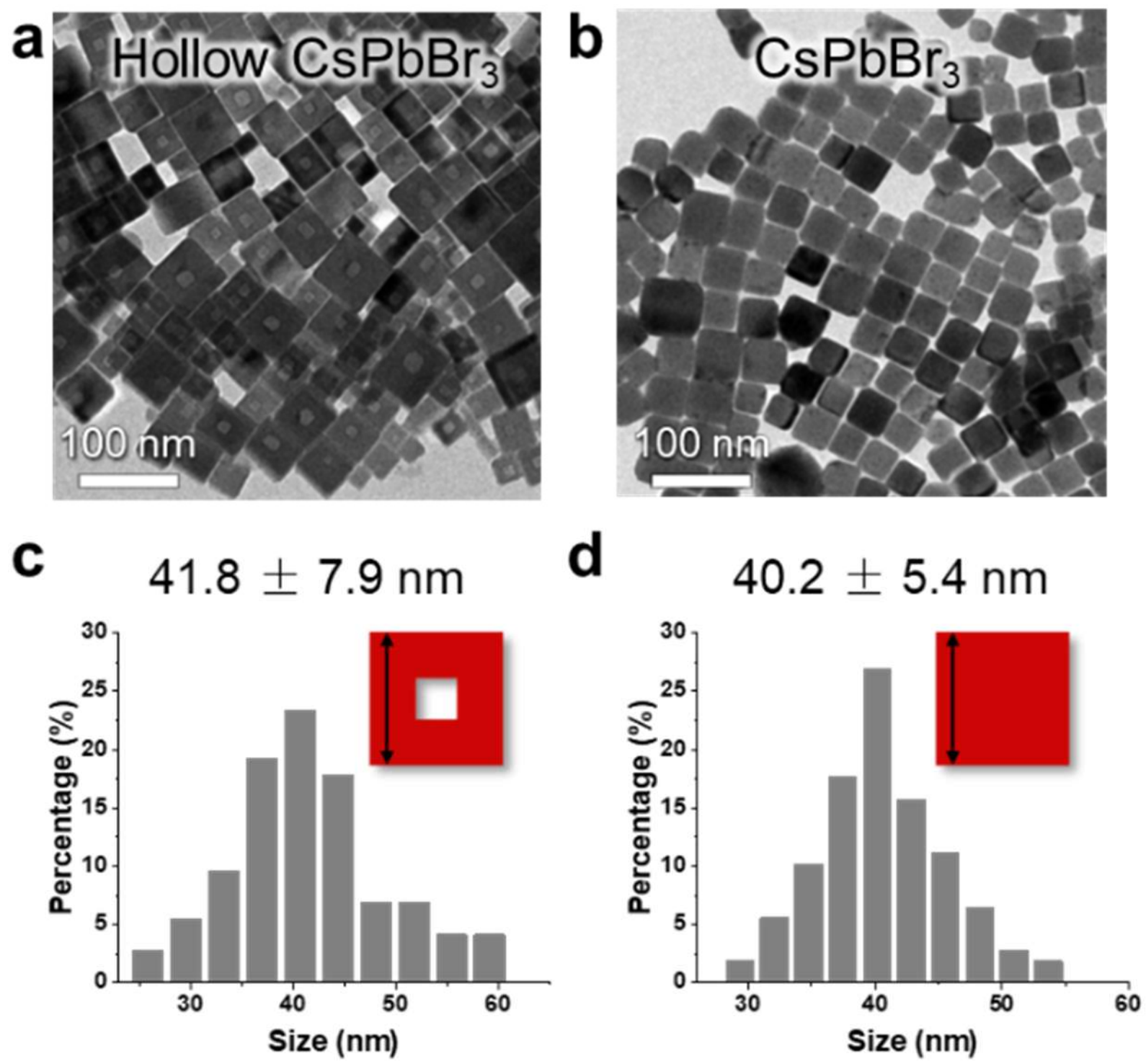

Figure S1. (a, b) TEM images and (c, d) size distributions of (a, c) hollow $CsPbBr_3$ nanocubes and (b, d) regular $CsPbBr_3$ nanocubes.

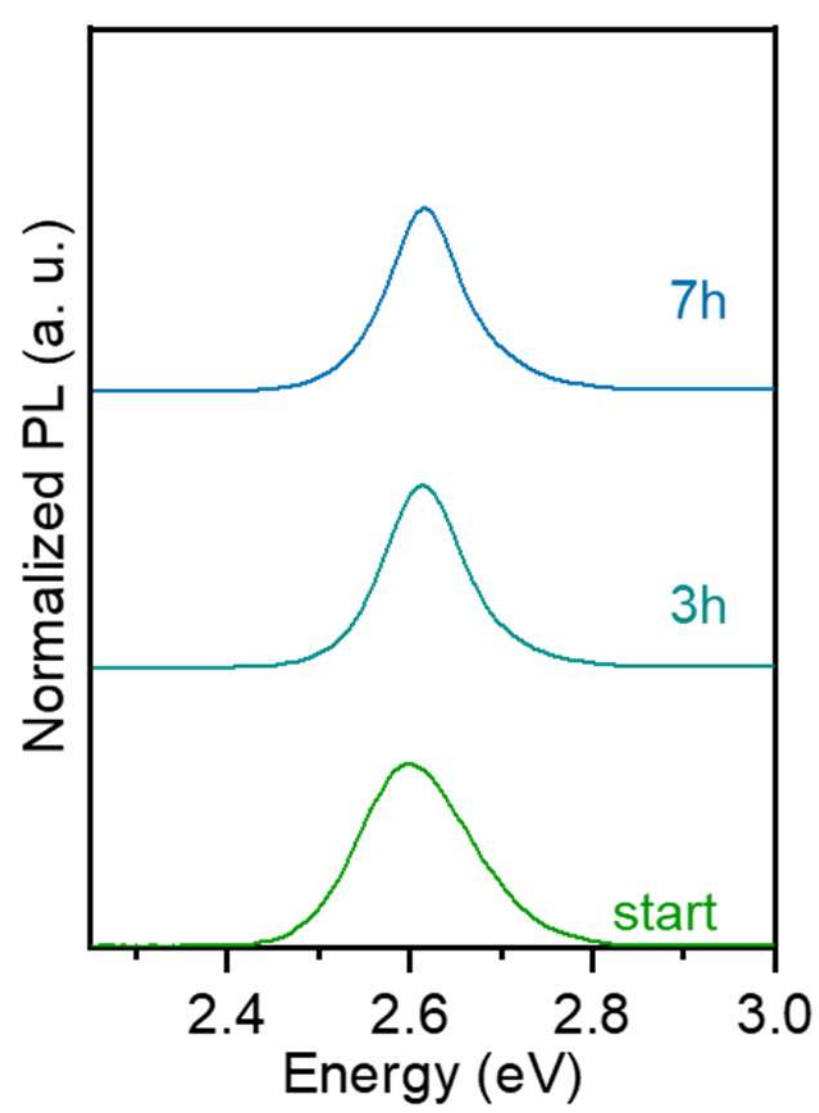

Figure S2. Time evolution of the PL spectrum in the solution sample of hollow $CsPb(Br:Cl)_3$ (Cl/Br = 0.4) nanocubes.

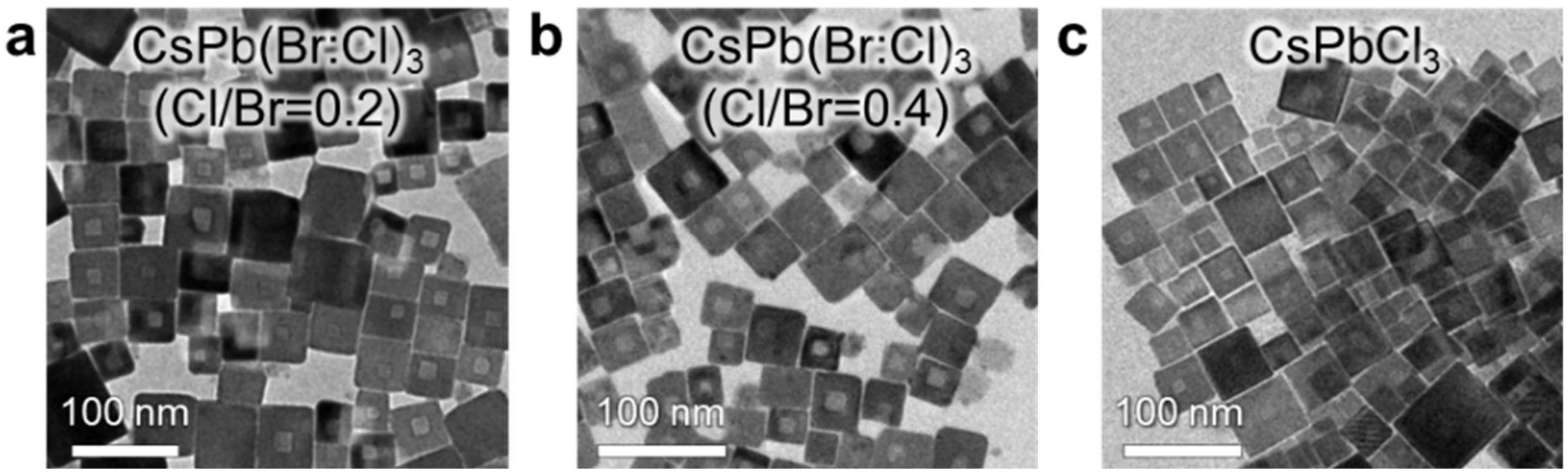

Figure S3. TEM images of hollow $CsPb(Br:Cl)_3$ (Cl/Br = 0.2, 0.4) and $CsPbCl_3$ nanocubes in $Br^- \rightarrow Cl^-$ exchange.

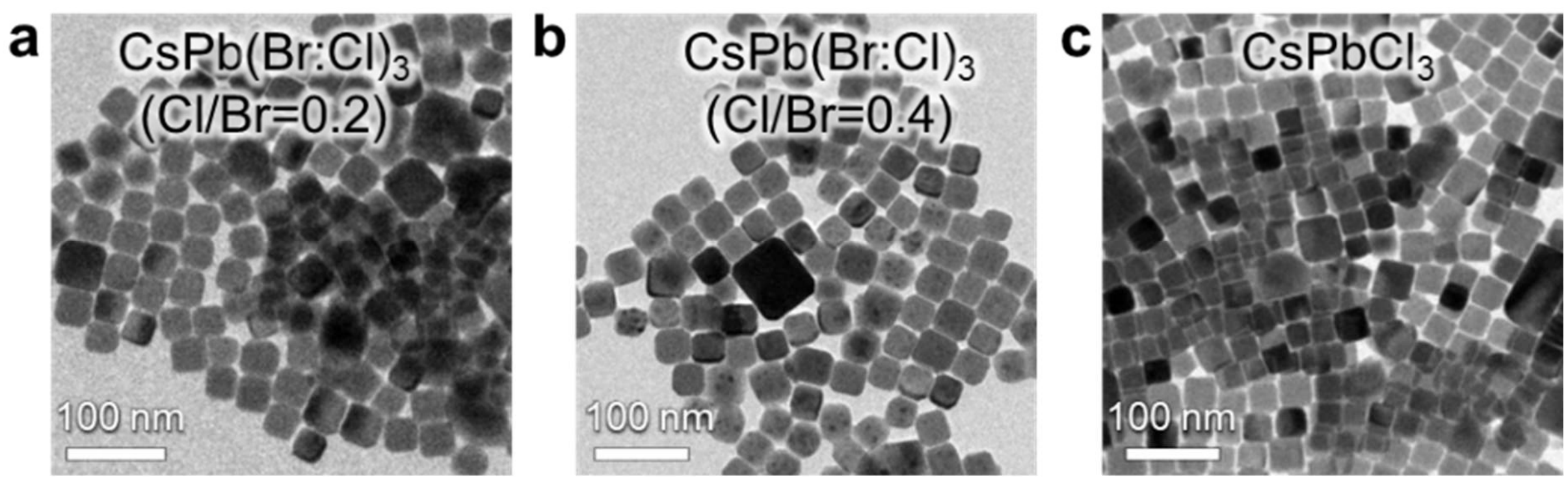

Figure S4. TEM images of regular $CsPb(Br:Cl)_3$ (Cl/Br = 0.2, 0.4) and $CsPbCl_3$ nanocubes in $Br^- \rightarrow Cl^-$ exchange.

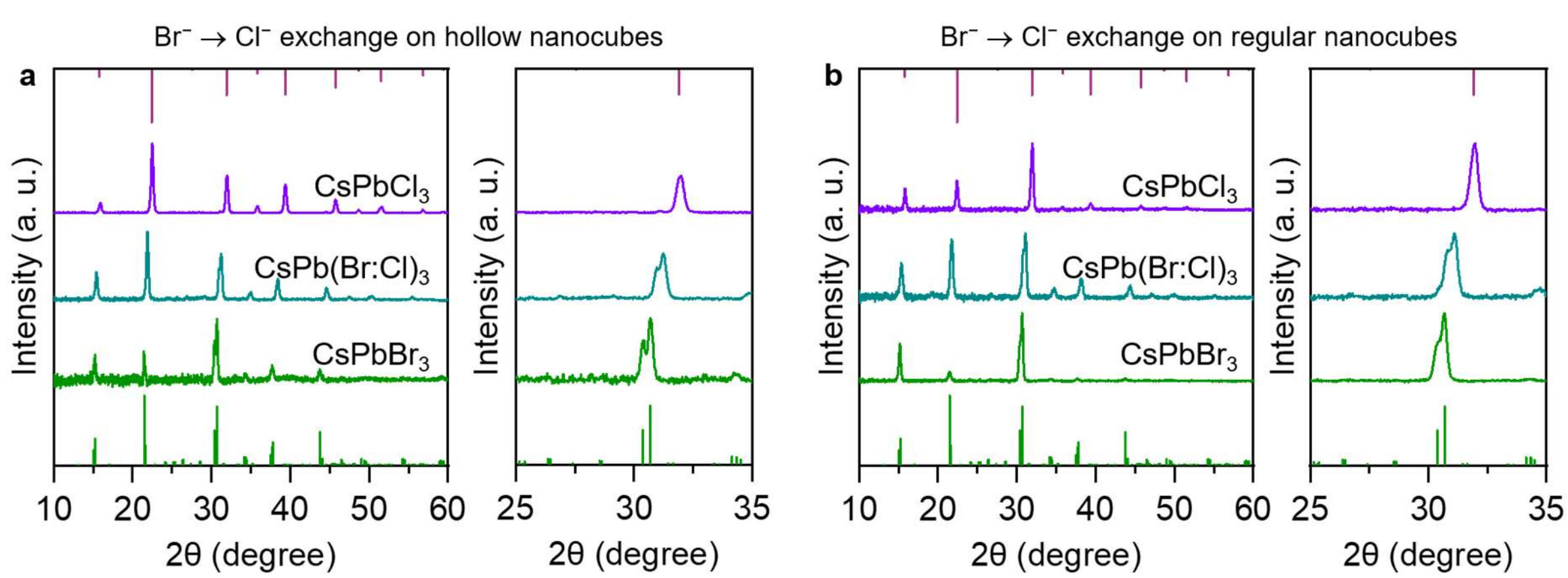

Figure S5. XRD patterns and detailed view of the 2Θ=25°–35° range for (a) hollow $CsPbBr_3$, $CsPb(Br:Cl)_3$ (Cl/Br = 0.4) and $CsPbCl_3$ nanocubes and (b) regular $CsPbBr_3$, $CsPb(Br:Cl)_3$ (Cl/Br = 0.4) and $CsPbCl_3$ nanocubes in $Br^- \rightarrow Cl^-$ exchange. The green reference marks are for orthorhombic $CsPbBr_3$ (ICSD number 97851) and the purple ones are for cubic $CsPbCl_3$ (ICSD number 29072).

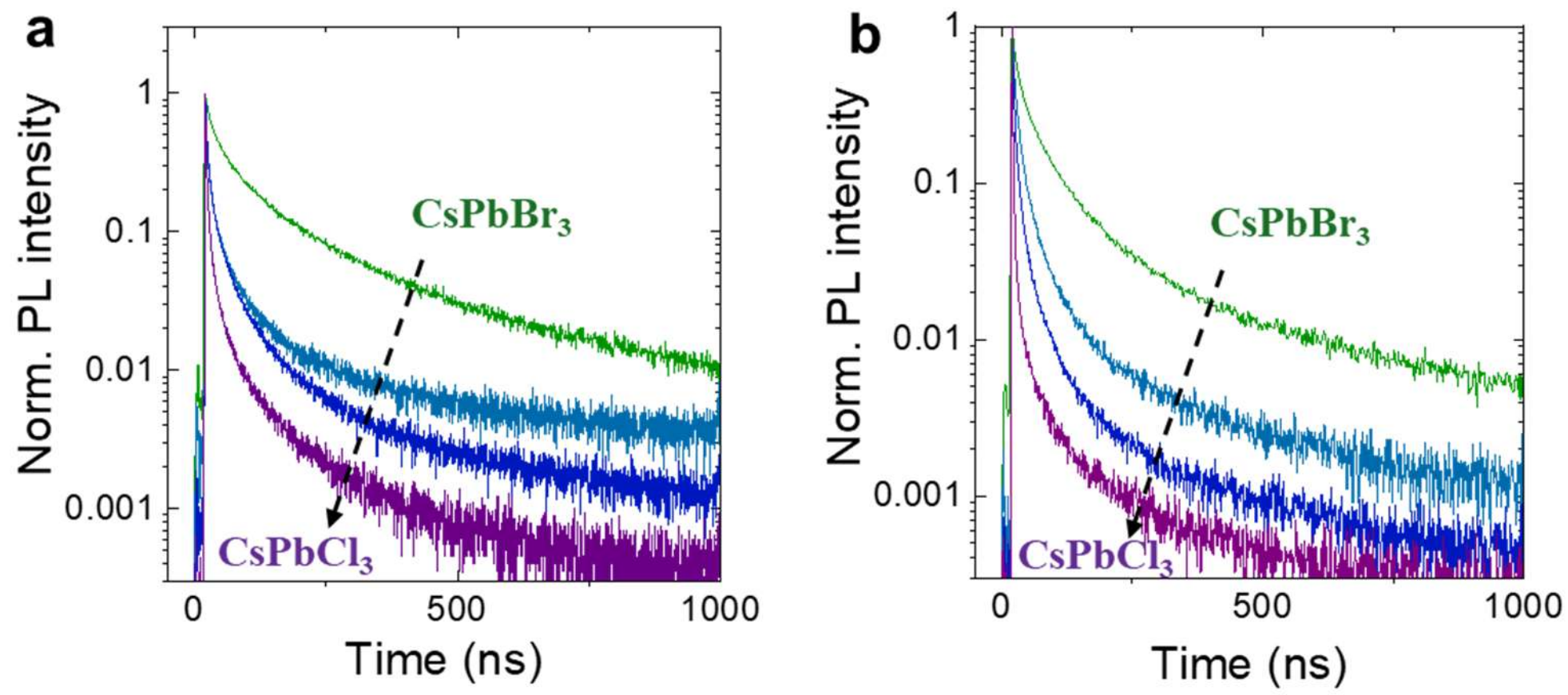

Figure S6. PL decay spectra of $CsPbBr_3$, $CsPb(Br:Cl)_3$ (Cl/Br = 0.4, 0.6), $CsPbCl_3$ for (a) hollow nanocubes and (b) regular nanocubes. The PL decay curves of the samples were measured at the PL peak maximum, as shown in Figure 1b,f.

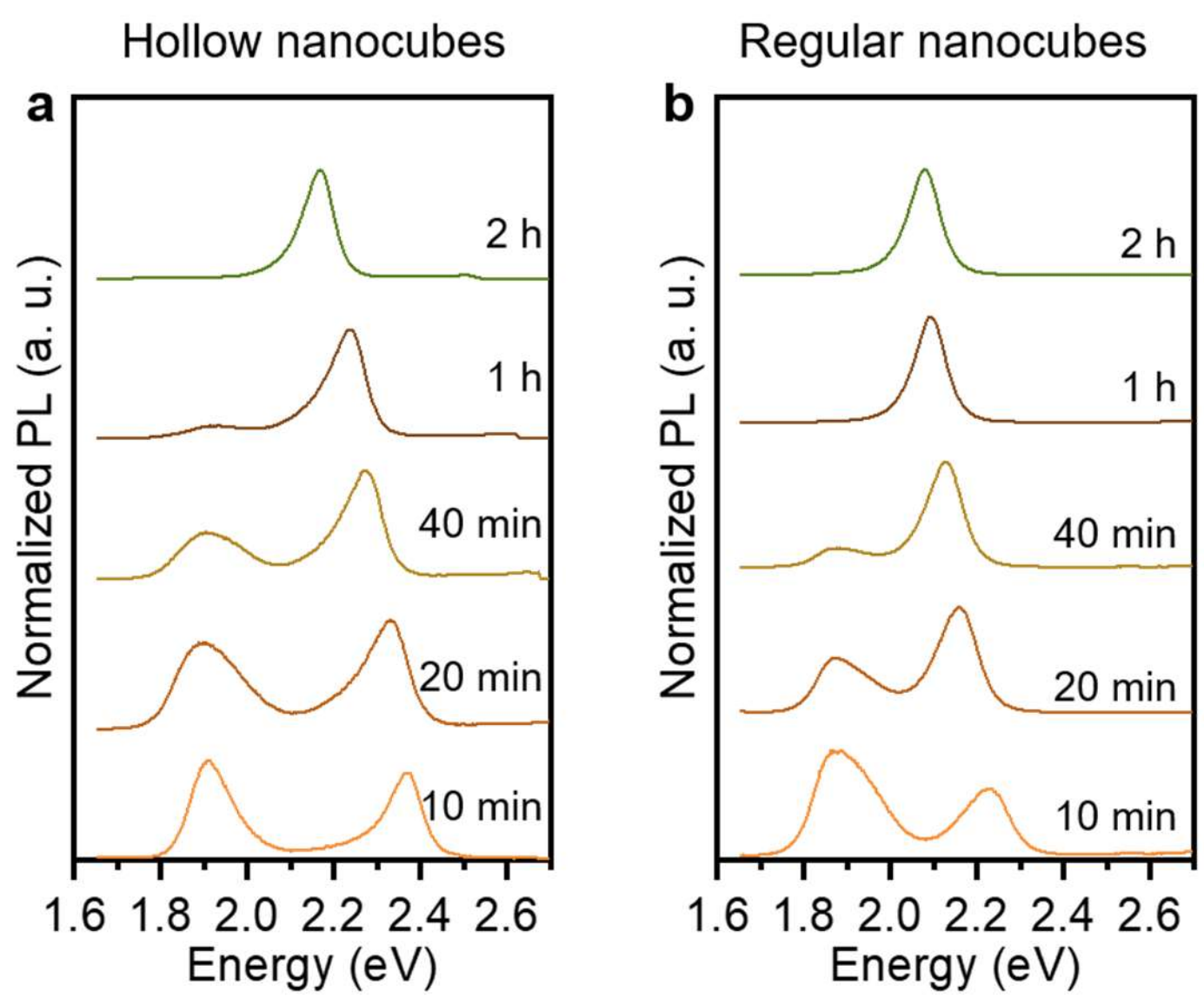

Figure S7. PL evolution of the intermediate (a) $CsPb(I:Br)_3$ (Br/I = 0.4) hollow nanocubes and (b) $CsPb(I:Br)_3$ (Br/I = 0.4) regular nanocubes at different reaction times (10 min, 20 min, 40 min, 1 hour and 2 hours).

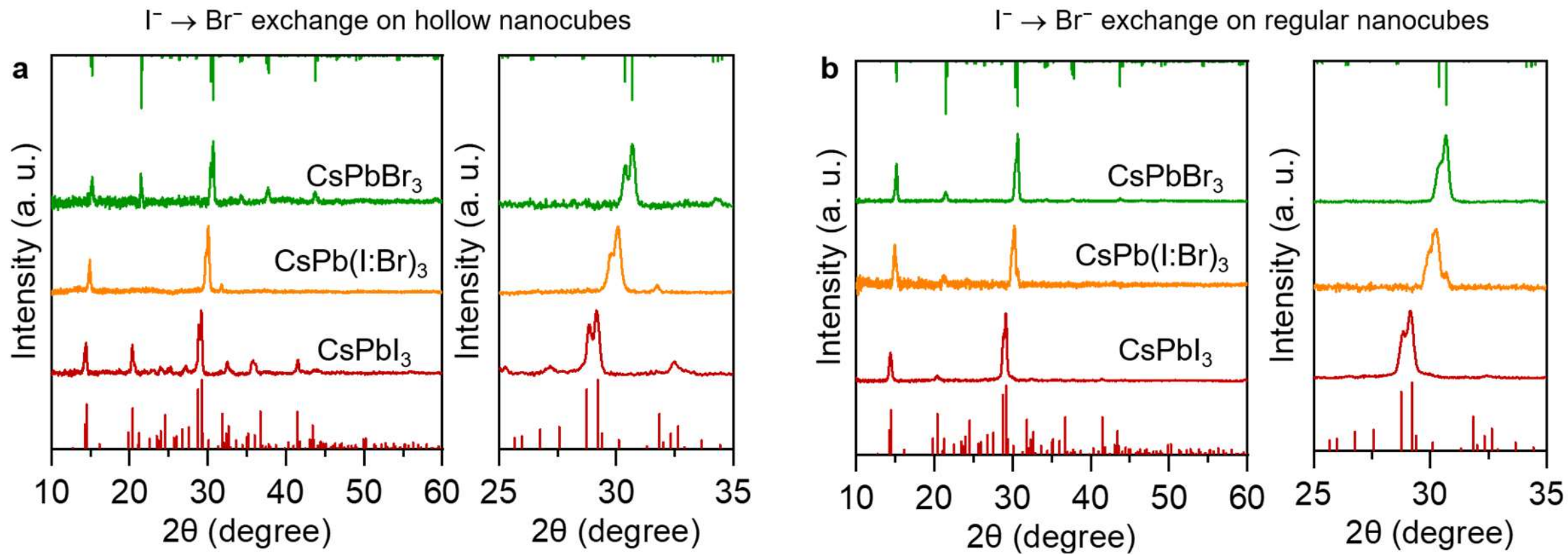

Figure S8. XRD patterns and detailed view of the 2Θ=25°–35° range for (a) hollow $CsPbI_3$, $CsPb(I:Br)_3$ (Br/I = 0.4) and $CsPbBr_3$ nanocubes and (b) regular $CsPbI_3$, $CsPb(I:Br)_3$ (Br/I = 0.4) and $CsPbBr_3$ nanocubes in $I^- \rightarrow Br^-$ exchange. The red reference marks are for orthorhombic $CsPbI_3$ (ICSD number 69423).

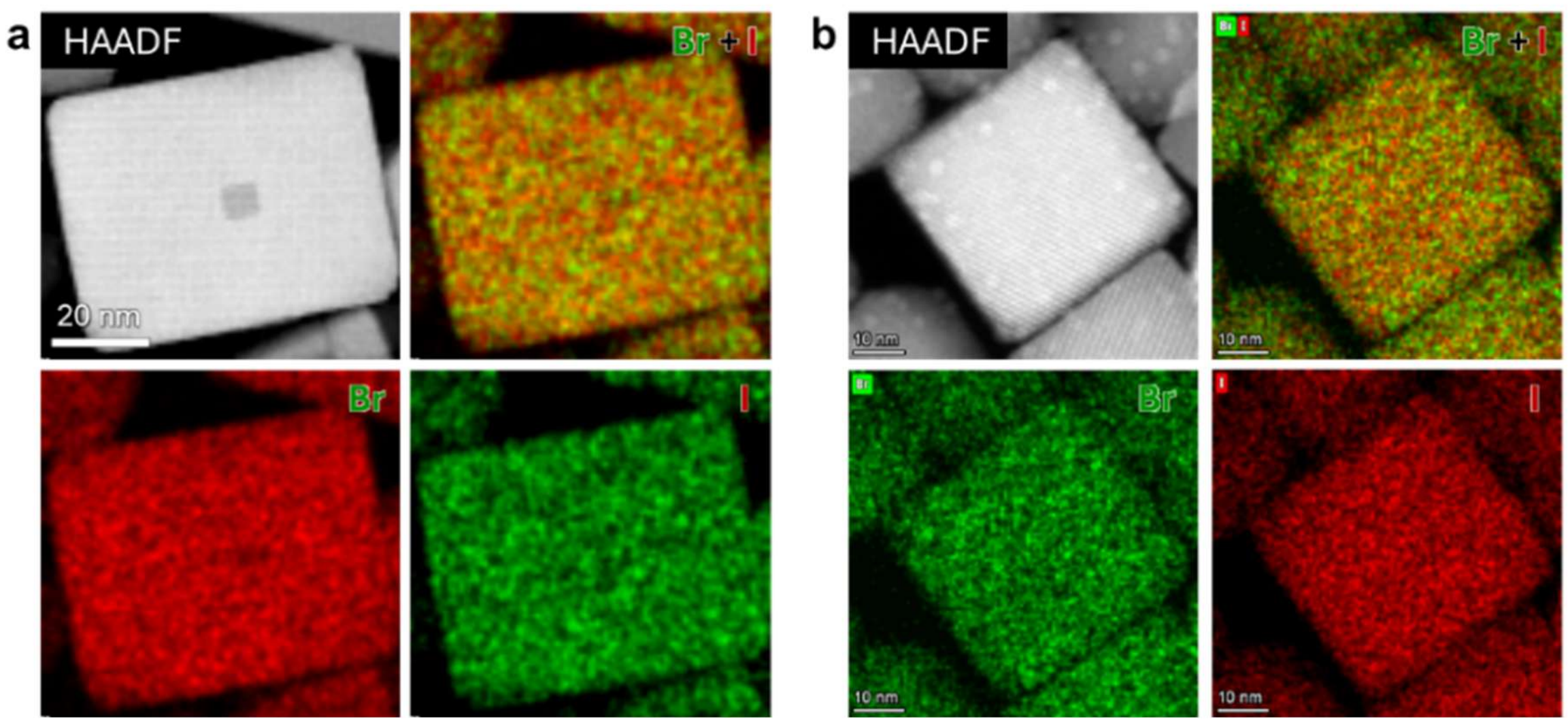

Figure S9. STEM-EDS elemental maps of samples of (a) hollow $CsPb(I:Br)_3$ (Br/I = 0.4) nanocubes and (b) regular $CsPb(I:Br)_3$ (Br/I = 0.4) nanocubes after 7 h.

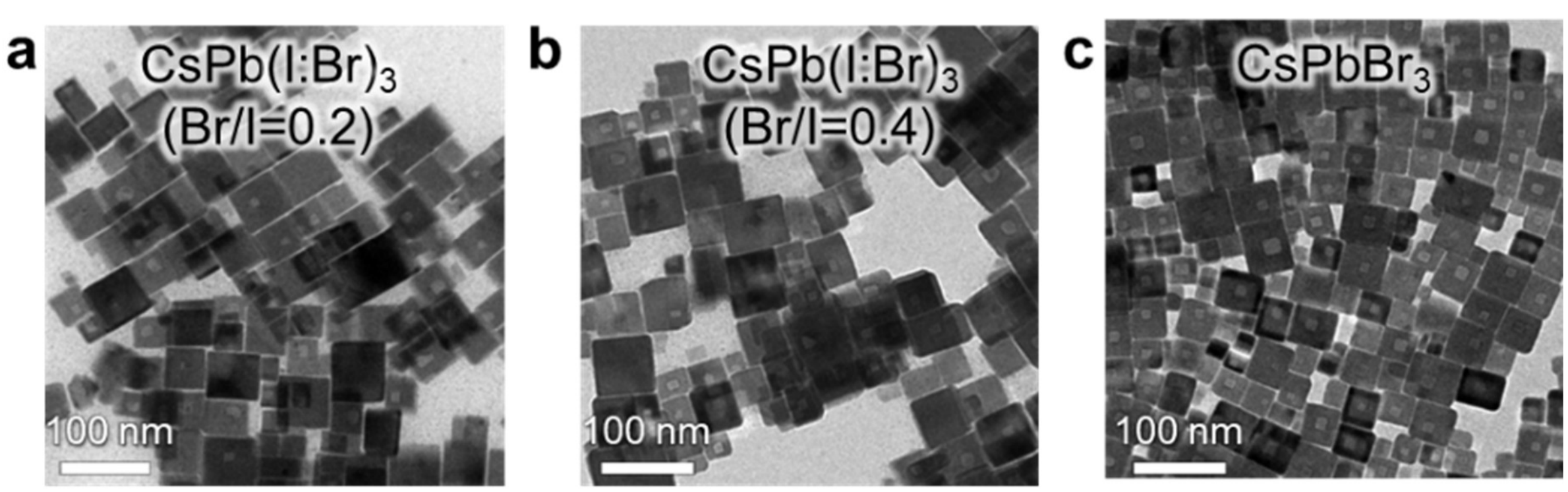

Figure S10. TEM images of (a, b) hollow $CsPb(I:Br)_3$ (Br/I = 0.2, 0.4) and (c) $CsPbBr_3$ nanocubes in $I^- \rightarrow Br^-$ exchange.

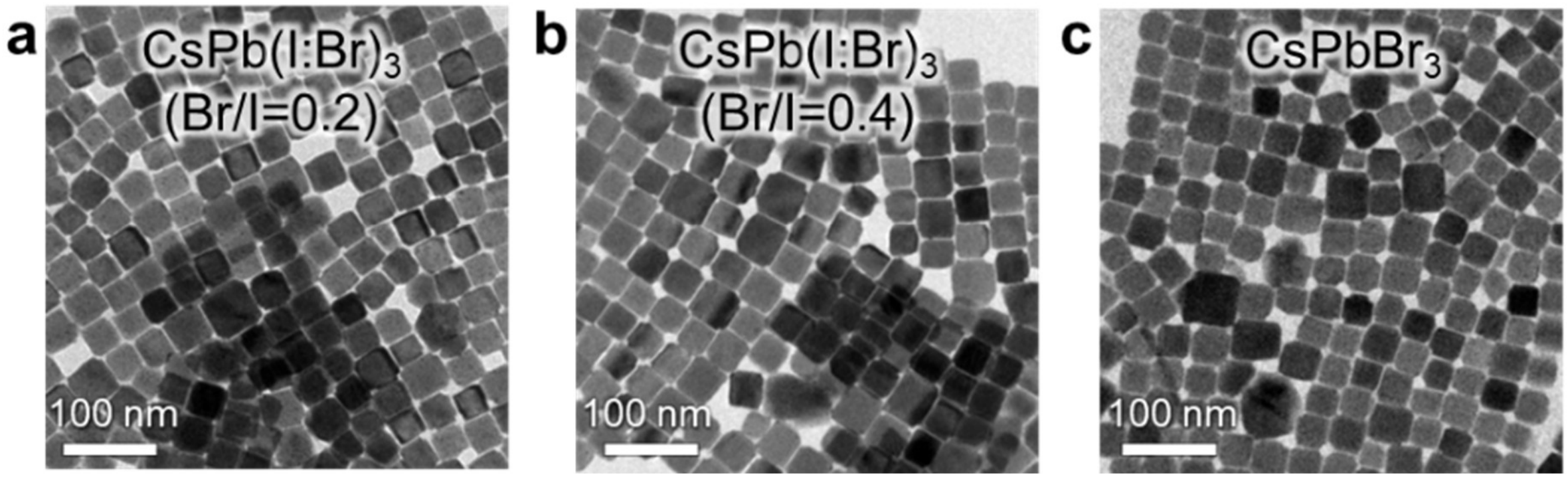

Figure S11. TEM images of (a, b) regular CsPb(I:Br)$_3$ (Br/I = 0.2, 0.4) and (c) $CsPbBr_3$ nanocubes in $I^- \rightarrow Br^-$ exchange.

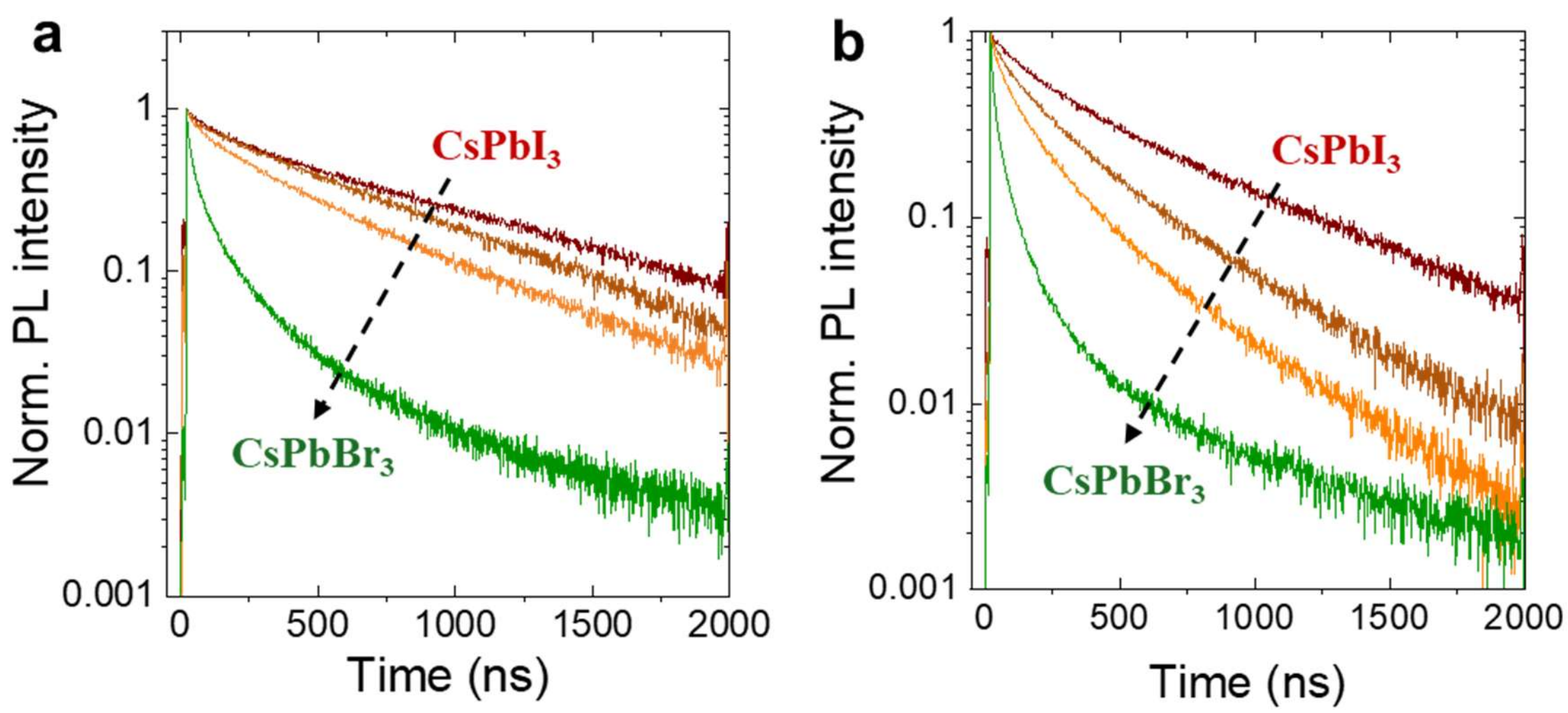

Figure S12. PL decay spectra of $CsPbI_3$, CsPb(I:Br)$_3$ (Br/I = 0.2, 0.4), $CsPbBr_3$ for (a) hollow nanocubes and (b) regular nanocubes. The PL decay curves of the $CsPbI_3$, CsPb(I:Br)$_3$ (Br/I = 0.2), $CsPbBr_3$ samples were measured at the corresponding PL peak maximum shown in Figure 2 b,g. For the CsPb(I:Br)$_3$ (Br/I = 0.4) sample, the PL decay curve was measured under the PL peak maximum (the emission peaks have merged into a single peak after 3 hours), as shown in Figure 2 c,h.

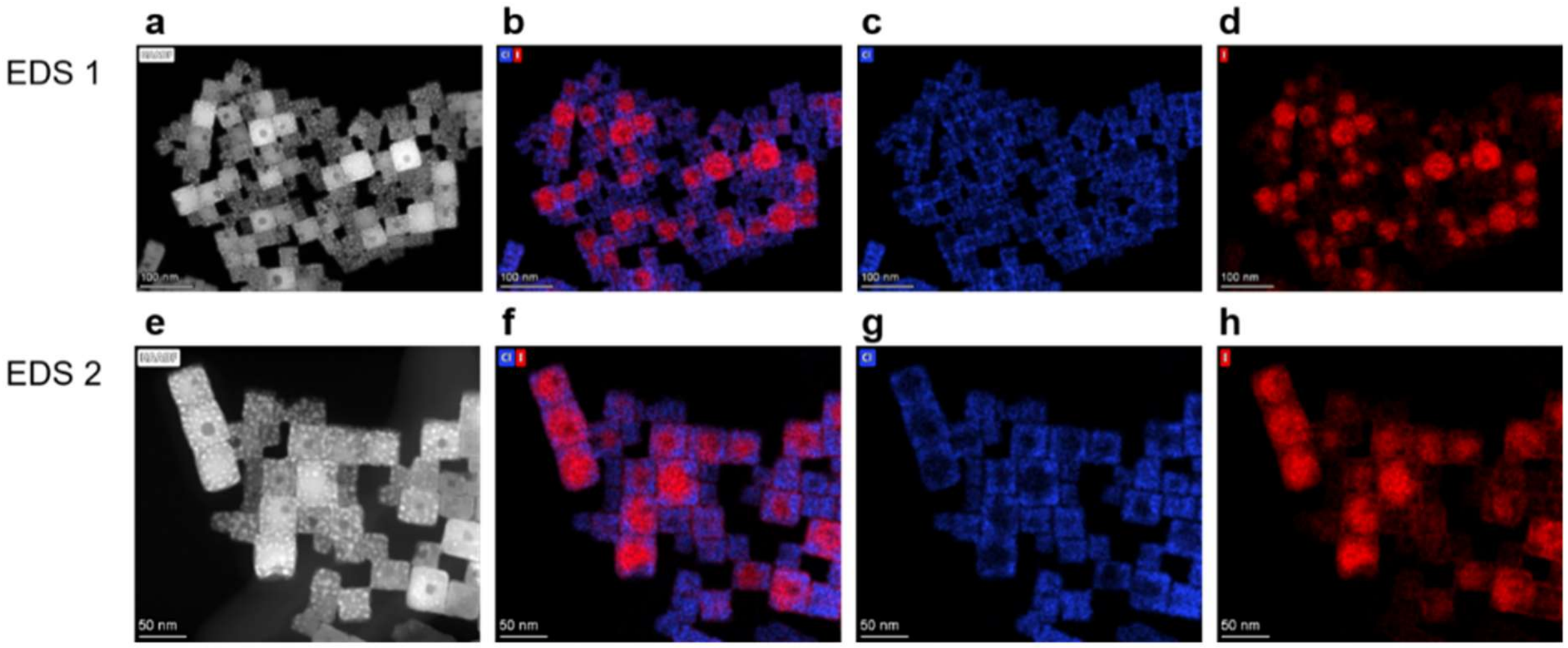

Figure S13. Large-area elemental analysis of $CsPbI_3/CsPbCl_3$ (Cl/I = 0.4) hollow nanocubes. HAADF-STEM image (a, e), composite EDS overlay map (b, f), and individual elemental maps for Cl^- (c, g) and I^- (d, h) recorded across a broad ensemble.

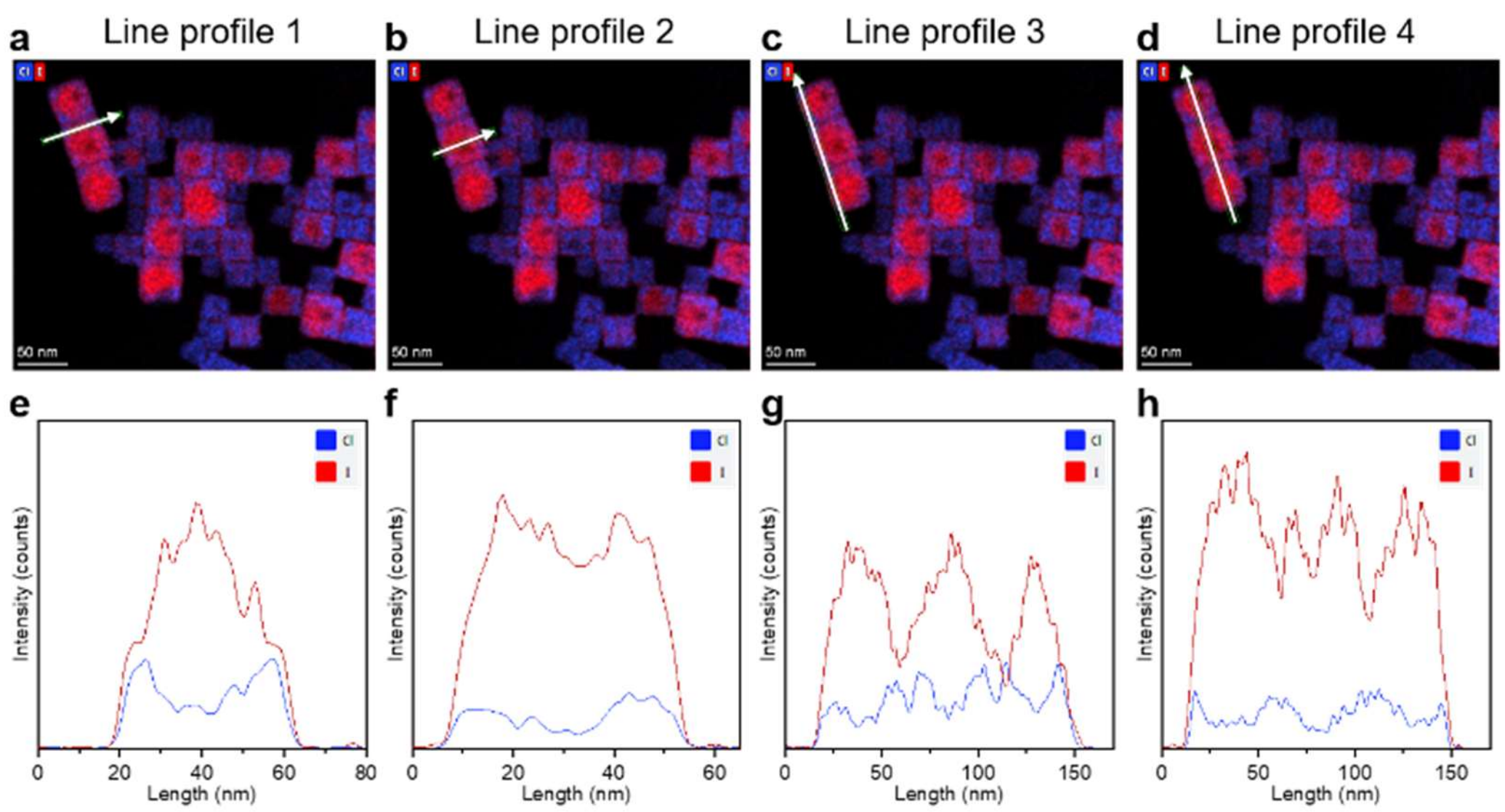

Figure S14. Line profile analysis of EDS signal in $CsPbI_3/CsPbCl_3$ (Cl/I = 0.4) hollow nanocubes. (a-d) Overlaid Cl^- and I^- elemental maps. The white arrows indicate the corresponding line scan extracted in the panel below across the corners (a, c) and the edges (b, d). (e-h) Corresponding spatial EDS intensity profiles of Cl^- (violet) and I^- (red) extracted along the line profiles 1-4 (e-h). The corners have a high Cl content; the relative Cl content in the middle of the facets is comparatively lower but still significant, demonstrating that a full shell is formed. Furthermore, the higher relative Cl^- intensity and increased shell thickness observed at the corner regions compared to the edge regions demonstrate that anion exchange proceeds faster at the corners than at the edges, while the exact thickness of Cl^- phase cannot be identified.

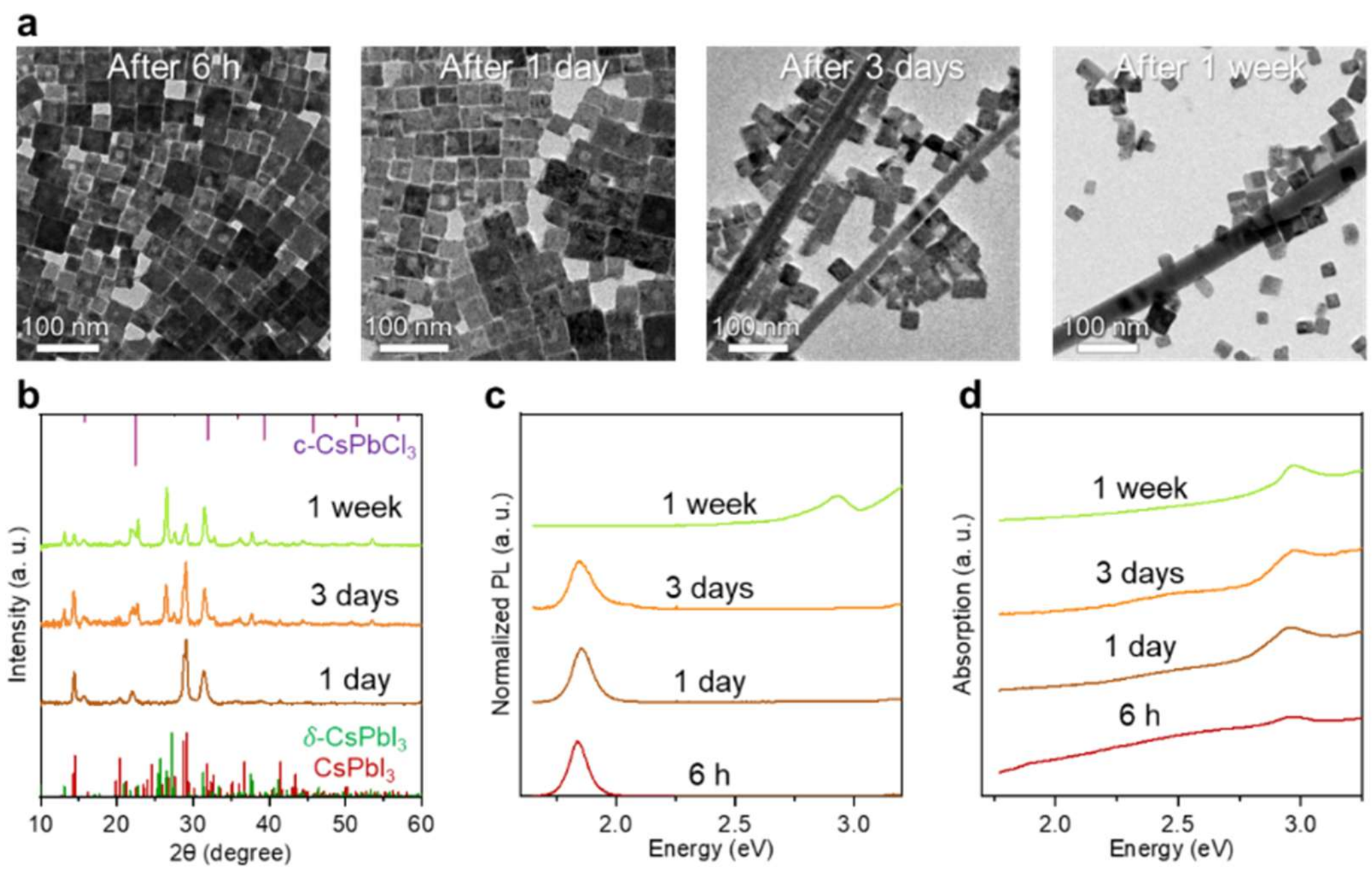

Figure S15. (a) TEM images, (b) XRD, (c) photoluminescence and (d) absorption spectra of the intermediate $CsPbI_3/CsPbCl_3$ (Cl/I = 0.4) hollow nanocubes after 6 h, 1 day, 3 days and 1 week.

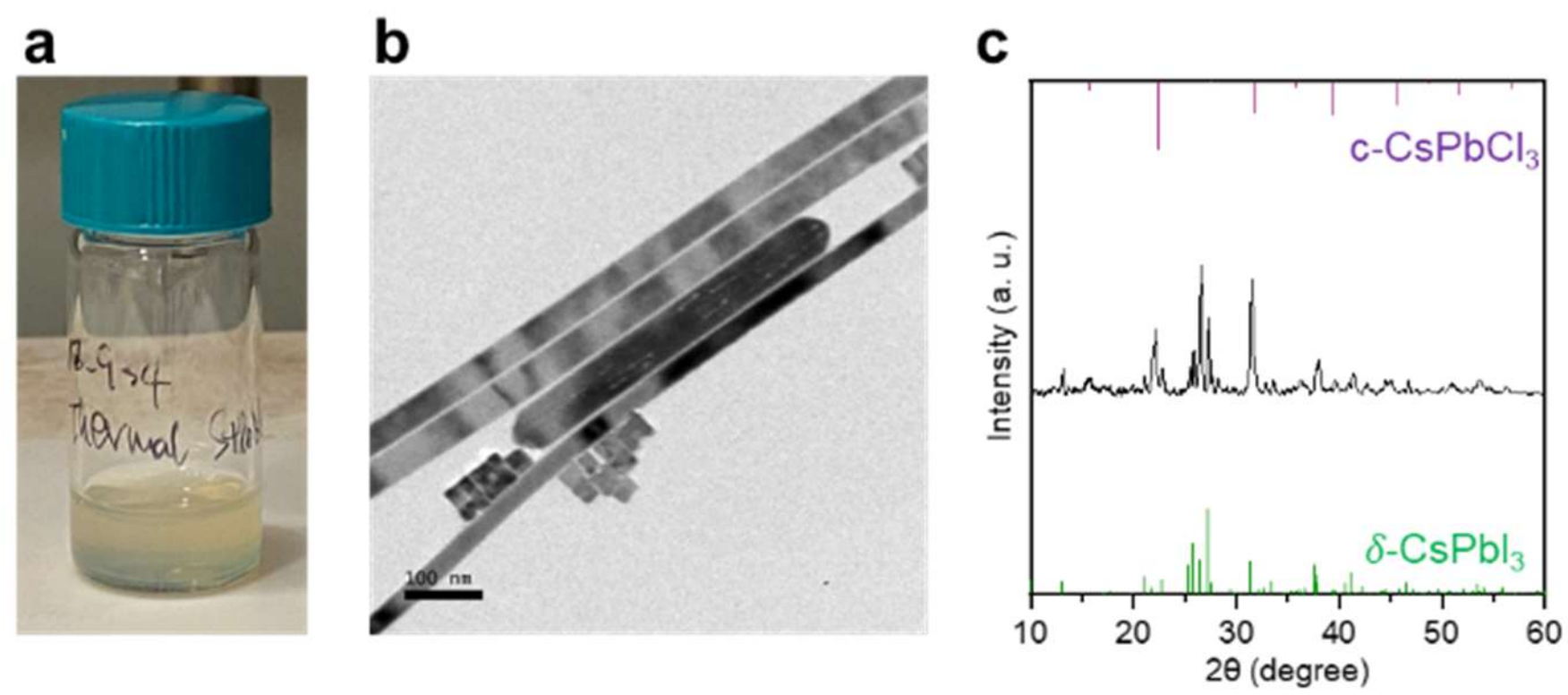

Figure S16. Thermal stability of $CsPbI_3/CsPbCl_3$ (Cl/I = 0.4) hollow nanocubes. (a) Digital photo, (b) TEM image and (c) XRD pattern of $CsPbI_3/CsPbCl_3$ sample after heating the nanocubes dispersion at 80 °C for 1 hour.

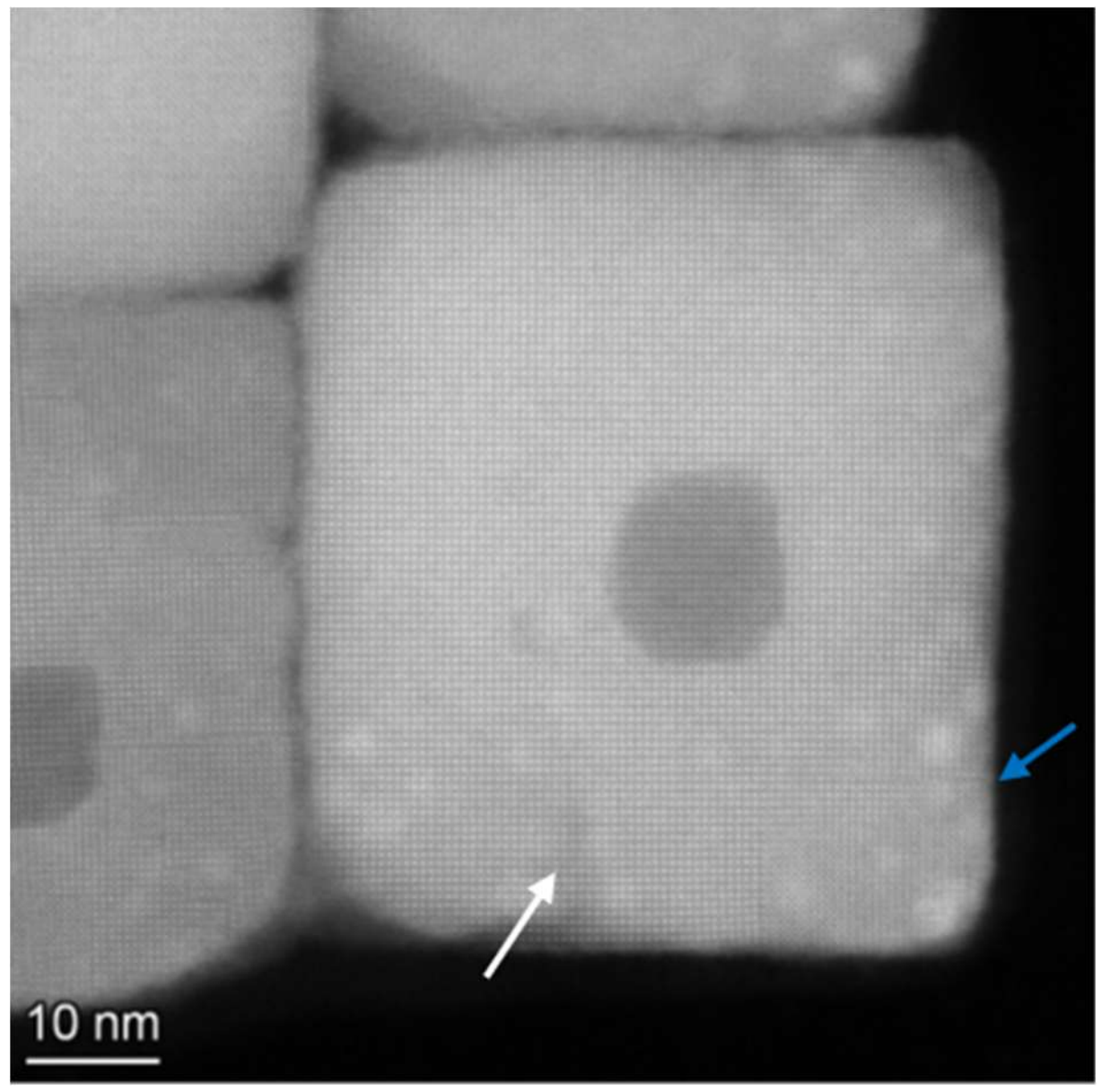

Figure S17. HAADF-STEM image of the hollow nanocubes at an early stage of $I^- \rightarrow Cl^-$ exchange. The onset of channel formation can be seen near the surface (marked with a white arrow). Also, the double Cs-I atomic planes can be seen at the edges/corners of the cube (marked with a blue arrow).

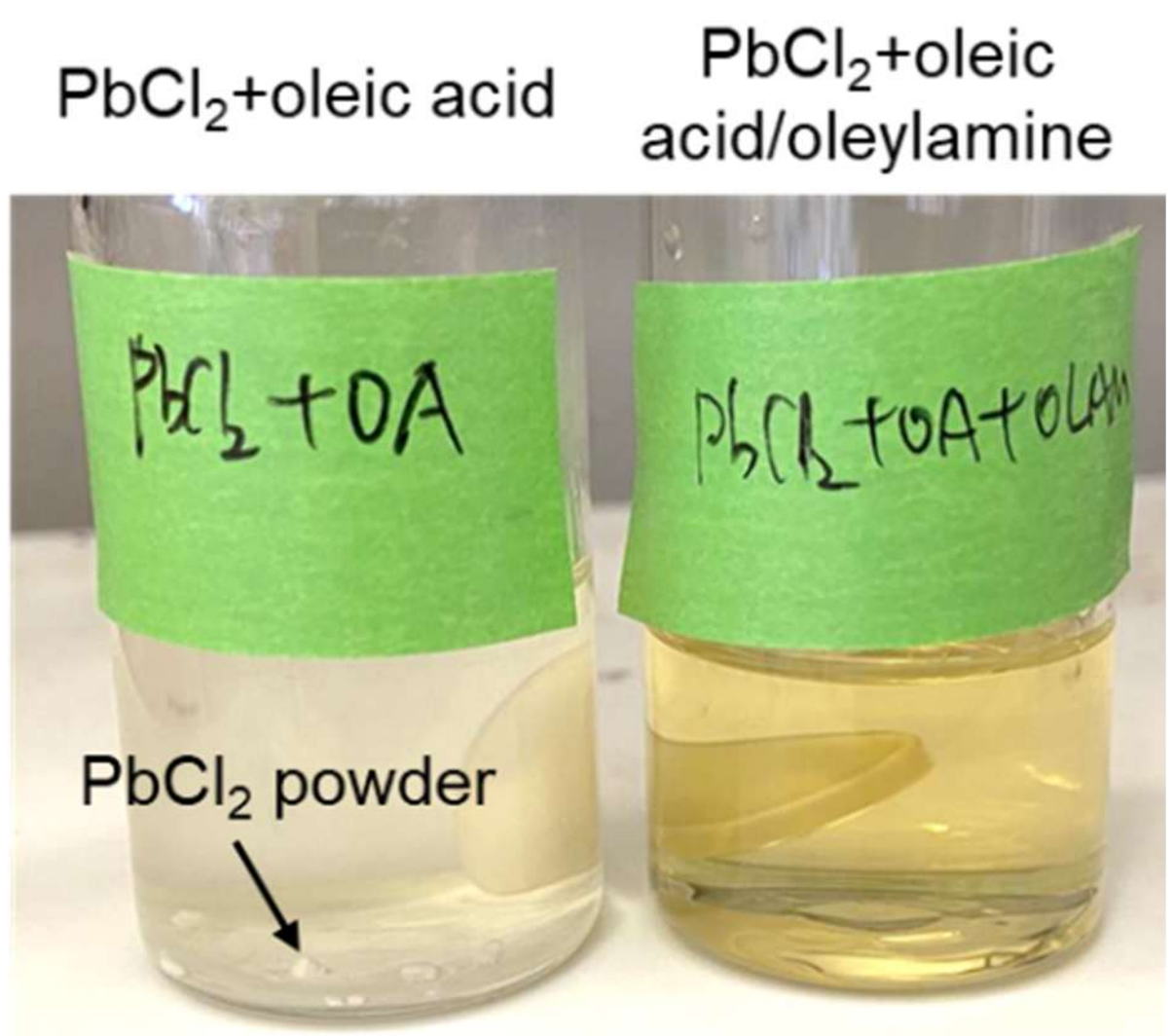

Figure S18. Digital photo of solutions of $PbCl_2$ + oleic acid and $PbCl_2$ + oleic acid/oleylamine.

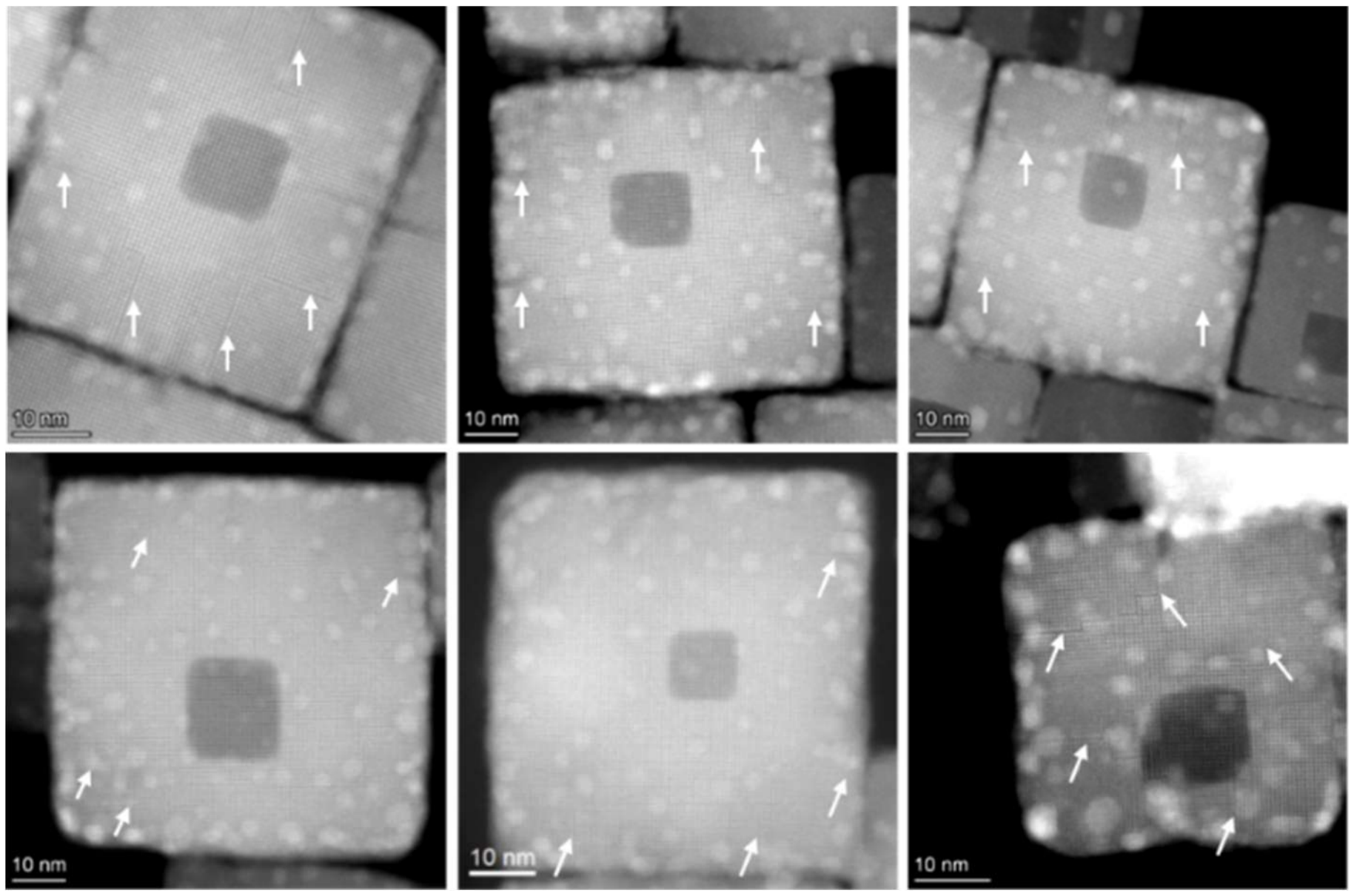

Figure S19. Atomic resolution HAADF-STEM image of hollow $CsPbI_3/CsPbCl_3$ nanocubes at an intermediate stage of exchange (Cl/I = 0.4). RP-type planar defects are indicated with white arrows.

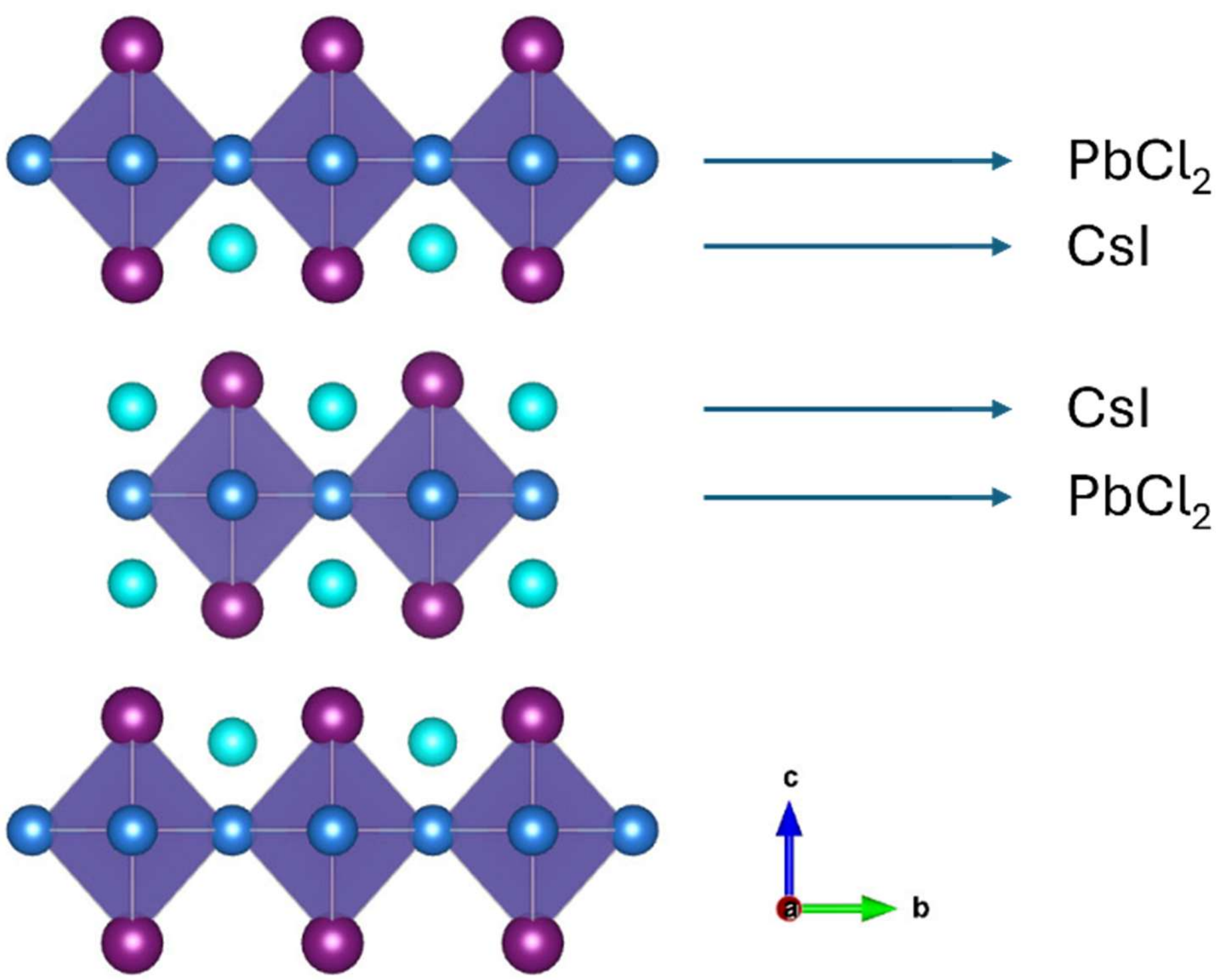

Figure S20. Ruddlesden-Popper (RP) $Cs_2PbI_2Cl_2$ phase. Cs^+ = cyan, I^- = purple, Cl^- = blue, and $[PbCl_4I_2]^{4-}$ octahedra = violet.

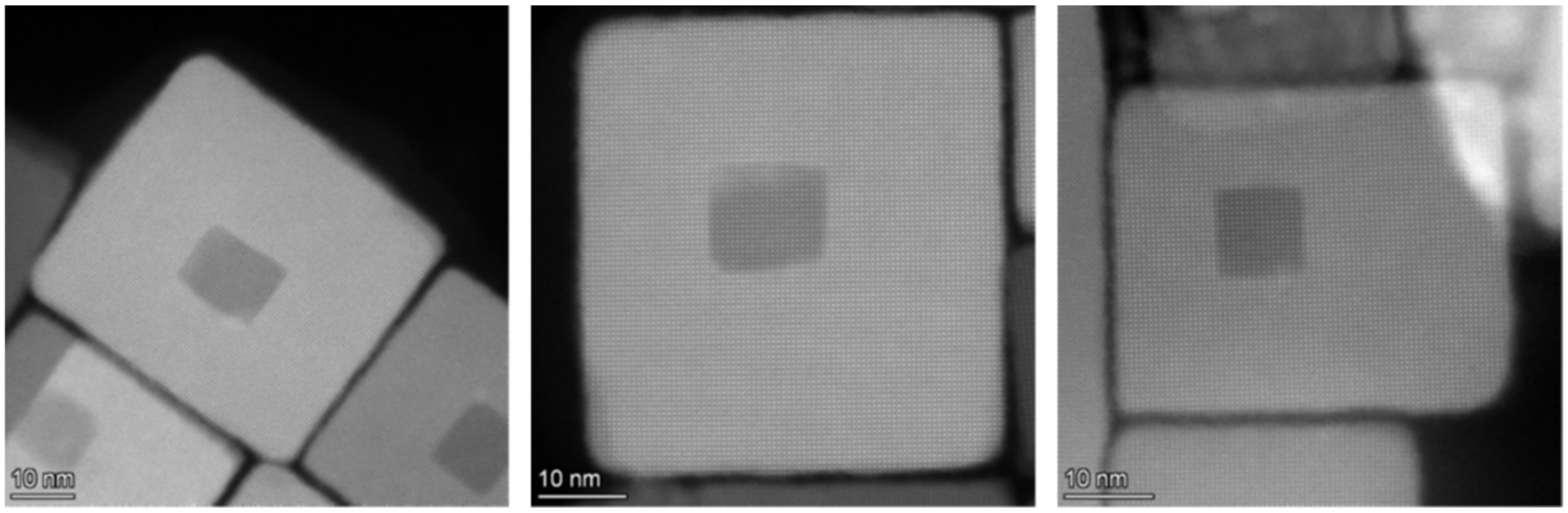

Figure S21. STEM images of the hollow $CsPbCl_3$ nanocubes after complete $I^- \rightarrow Cl^-$ exchange.

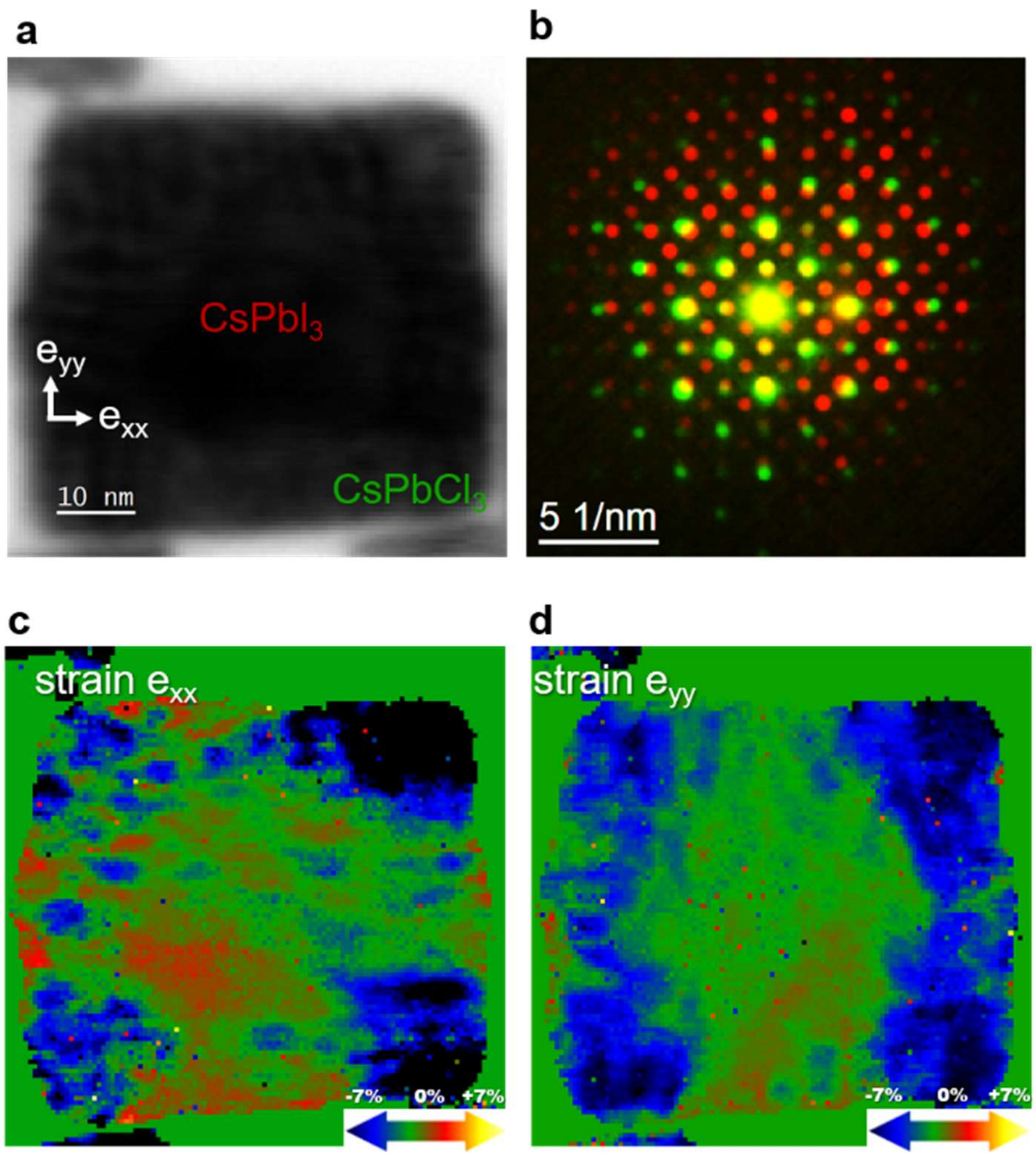

Figure S22. (a) 4D STEM local lattice parameter analysis for $CsPbI_3/CsPbCl_3$ (Cl/I = 0.4) hollow nanocube and (b) overlay of the local diffraction patterns from $CsPbI_3$ (red) and $CsPbCl_3$ (green) domains. (c, d) Variation of lattice parameters along two components (e_{xx} and e_{yy}) for the lattice strain in the nanocube. The total lattice parameter variation is a combination of a difference in relaxed lattices for the two phases and hydrostatic strain.

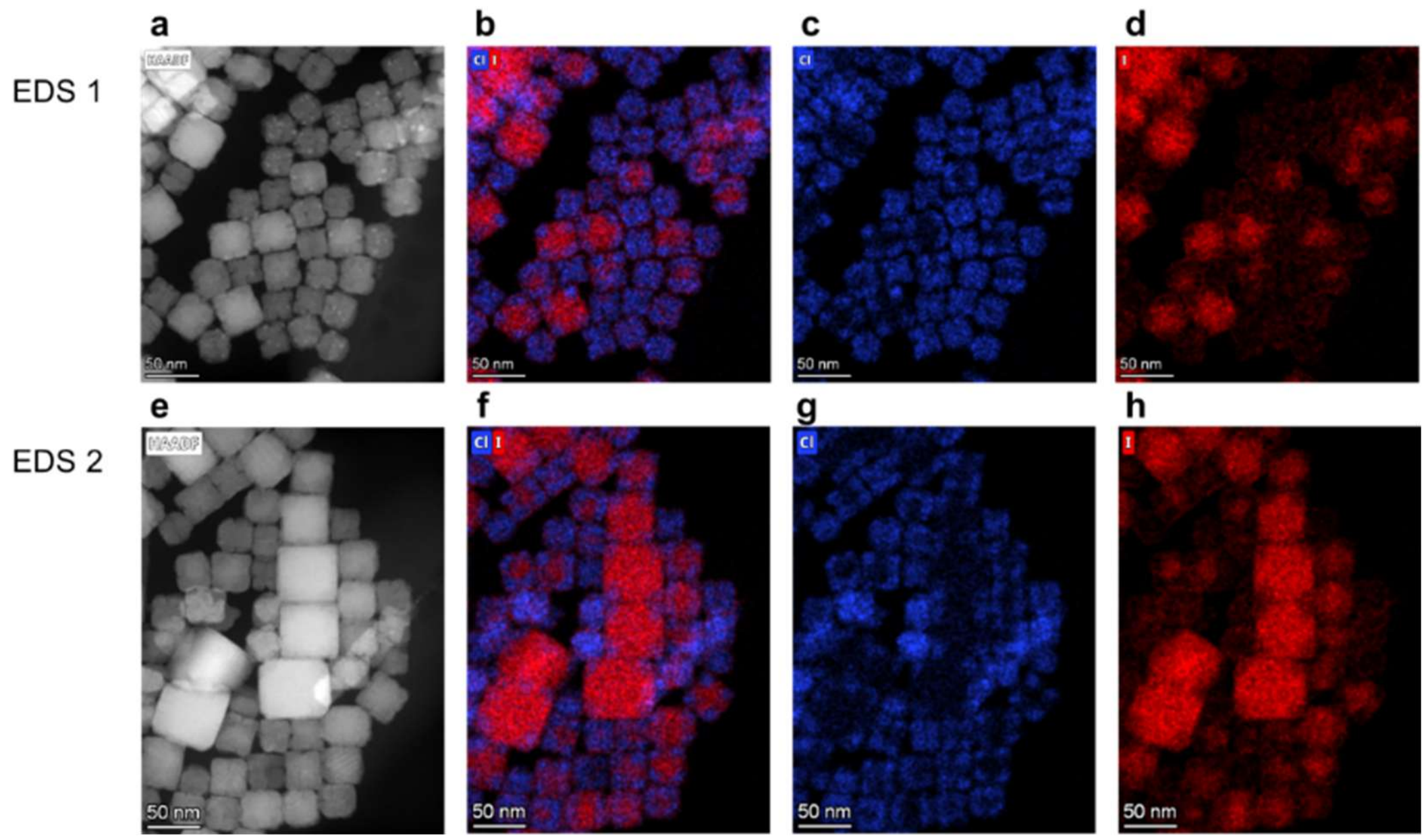

Figure S23. Large-area elemental analysis of $CsPbI_3/CsPbCl_3$ (Cl/I = 0.4) regular nanocubes. HAADF-STEM image (a, e), composite EDS overlay map (b, f), and individual elemental maps for Cl^- (c, g) and I^- (d, h) recorded across a broad ensemble.

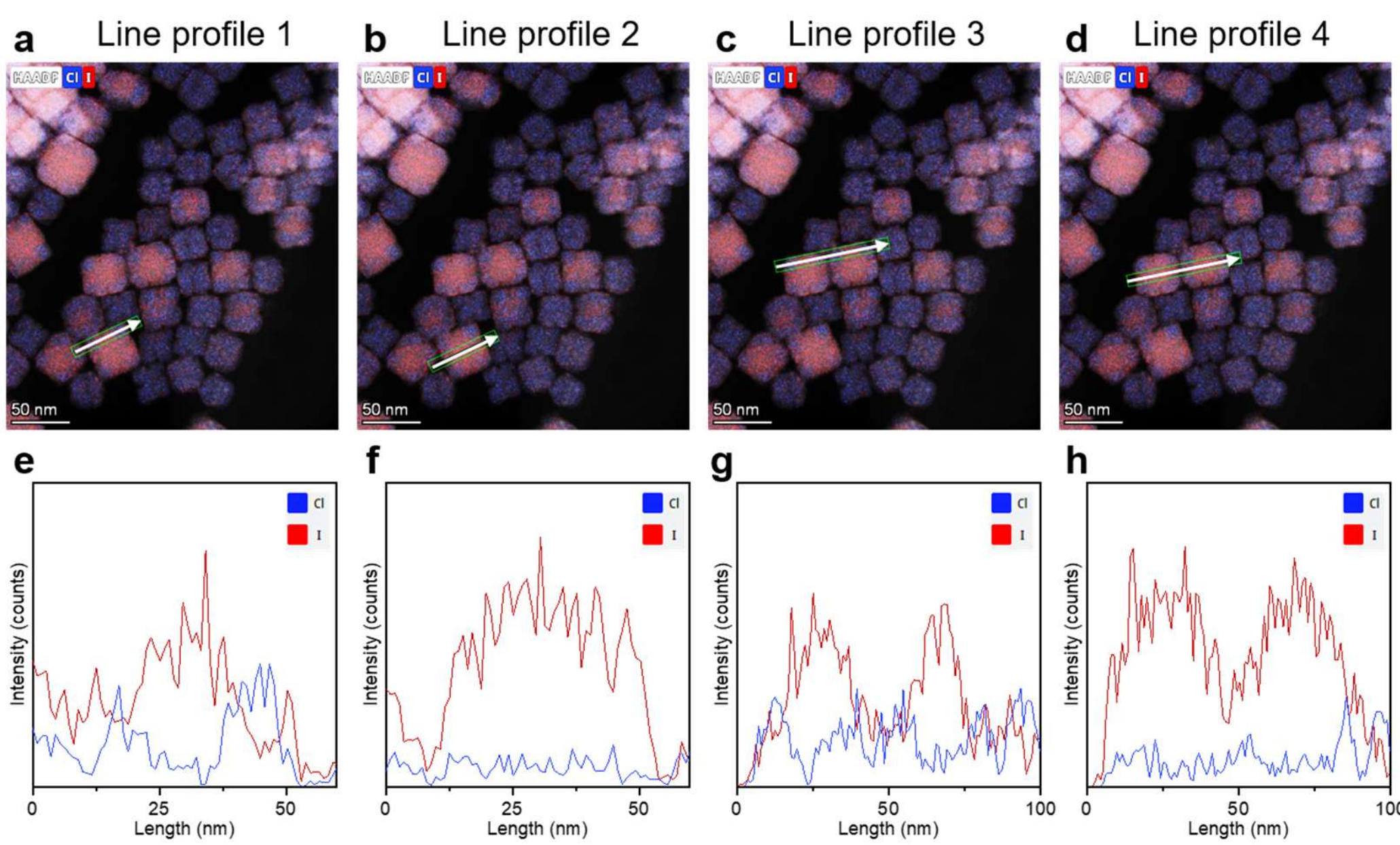

Figure S24. Line profiles of the EDS signal in $CsPbI_3/CsPbCl_3$ (Cl/I = 0.4) regular nanocubes. (a-d) Overlaid Cl^- and I^- elemental maps showing the linear scan tracks across representative regular nanocubes across the corners (a, c) and the edges (b, d). (e-h) Corresponding spatial EDS intensity profiles of Cl^- (violet) and I^- (red) extracted along the line profiles 1-4 (e-h). The line profiles confirm a continuous core-shell spatial architecture. Due to the broad size distribution, we found a size-dependent exchange kinetics, where smaller nanocubes progressed faster through the reaction. While chloride domains initially nucleate at the corners in larger cubes, they propagate along the edges in smaller ones quickly to coalesce into a continuous Cl^- rich shell around the $CsPbI_3$ core.

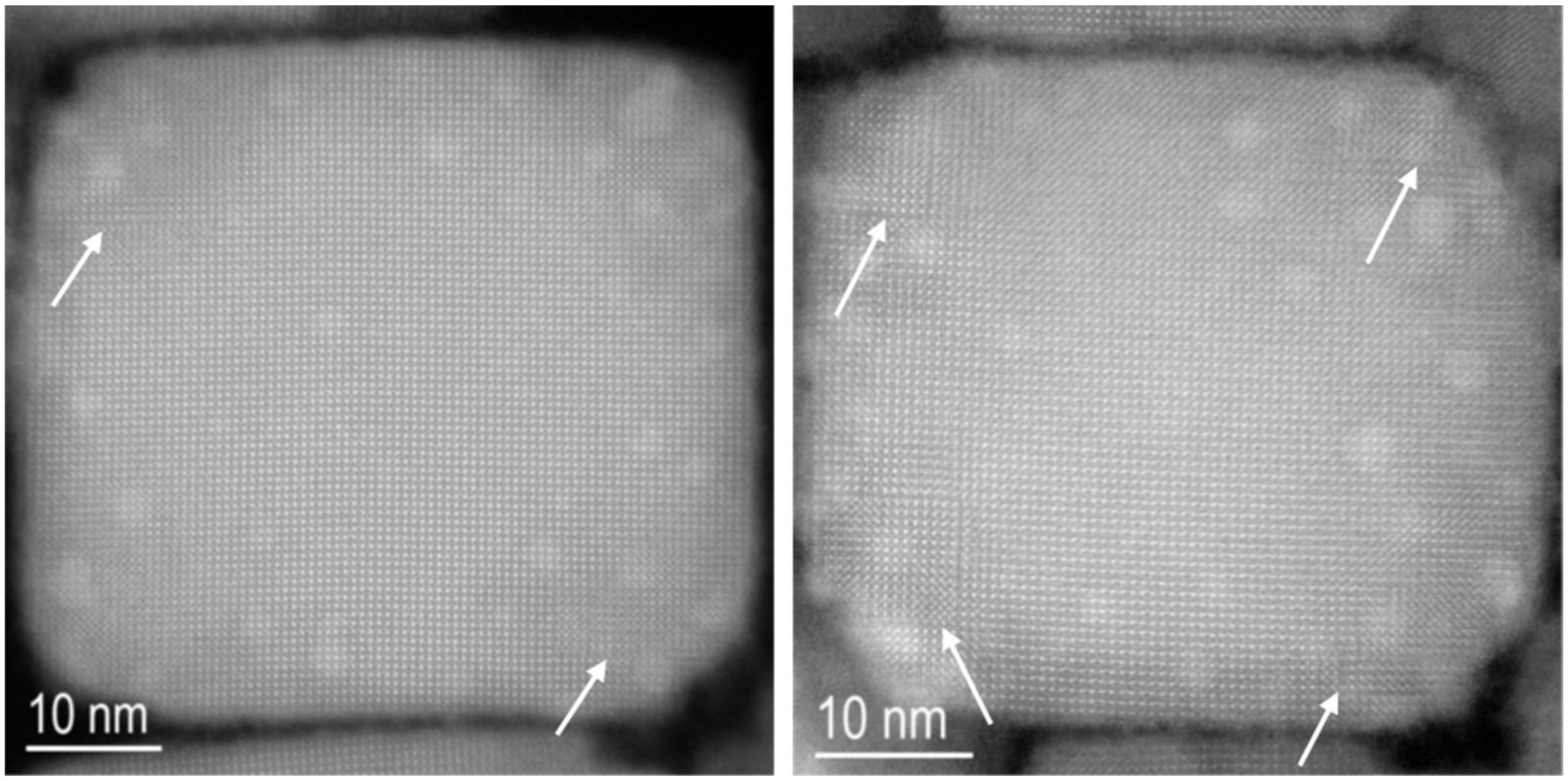

Figure S25. HAADF-STEM images of the regular nanocubes at an early stage of $I^- \rightarrow Cl^-$ exchange. The double Cs-I atomic planes can be seen at the edges/corners of the cubes (marked with white arrows).

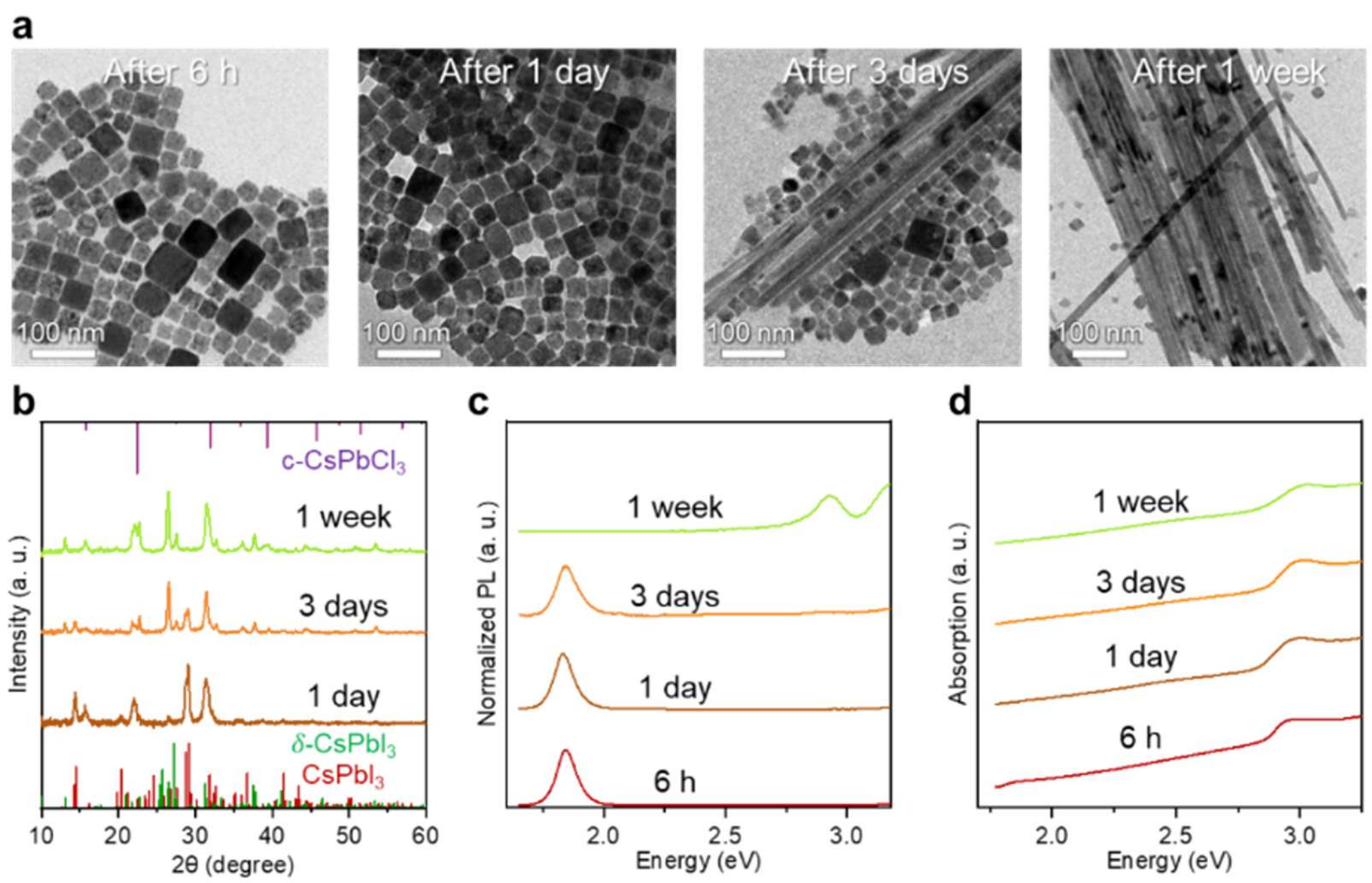

Figure S26. (a) TEM images, (b) XRD, (c) photoluminescence spectra and (d) absorption spectra of the intermediate $CsPbI_3/CsPbCl_3$ (Cl/I = 0.4) regular nanocubes after 6 h, 1 day, 3 days and 1 week.

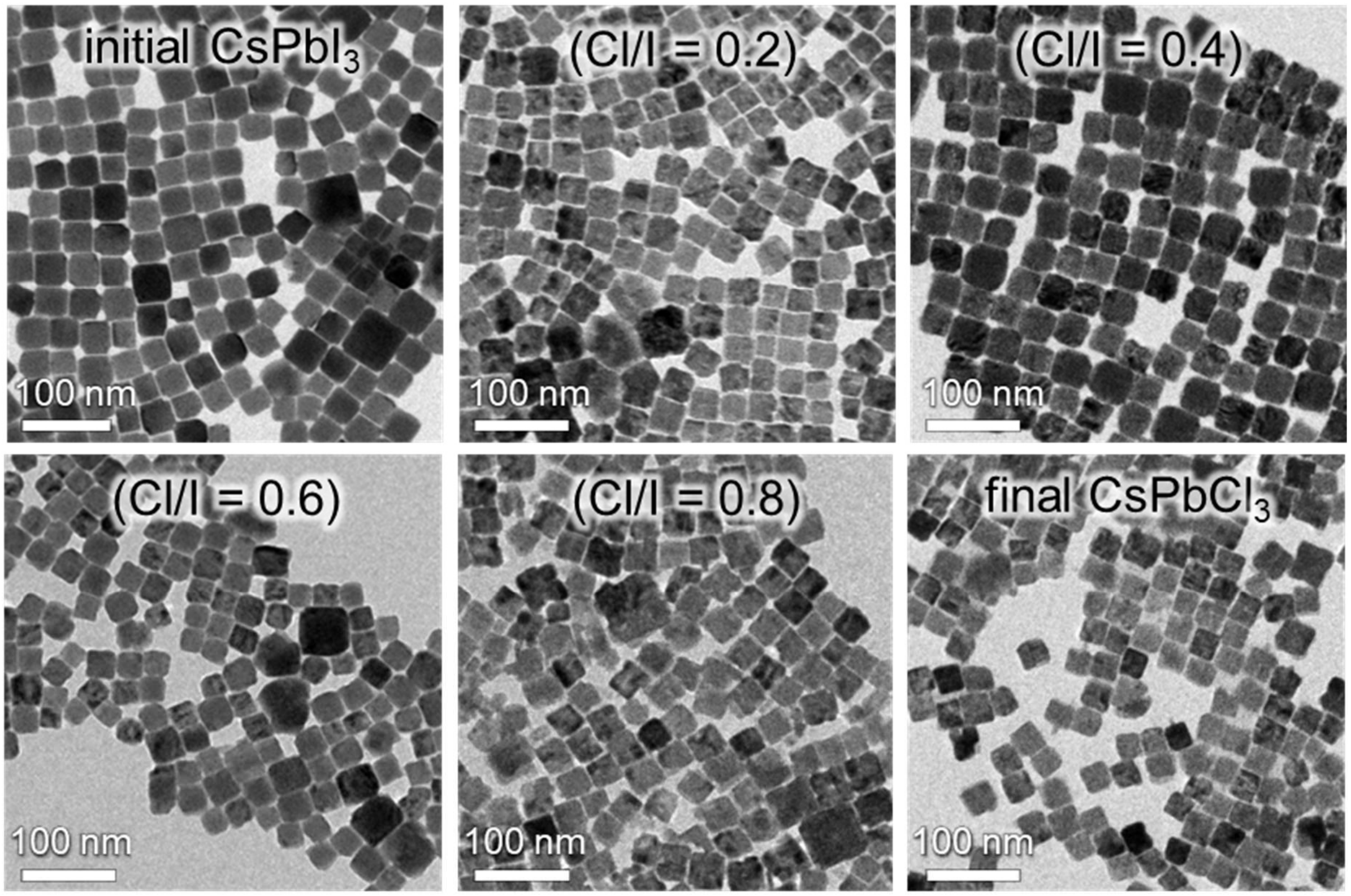

Figure S27. TEM images of regular $CsPbI_3$, $CsPbI_3/CsPbCl_3$ (Cl/I = 0.2, 0.4, 0.6, 0.8) and $CsPbCl_3$ nanocubes in $I^- \rightarrow Cl^-$ exchange.

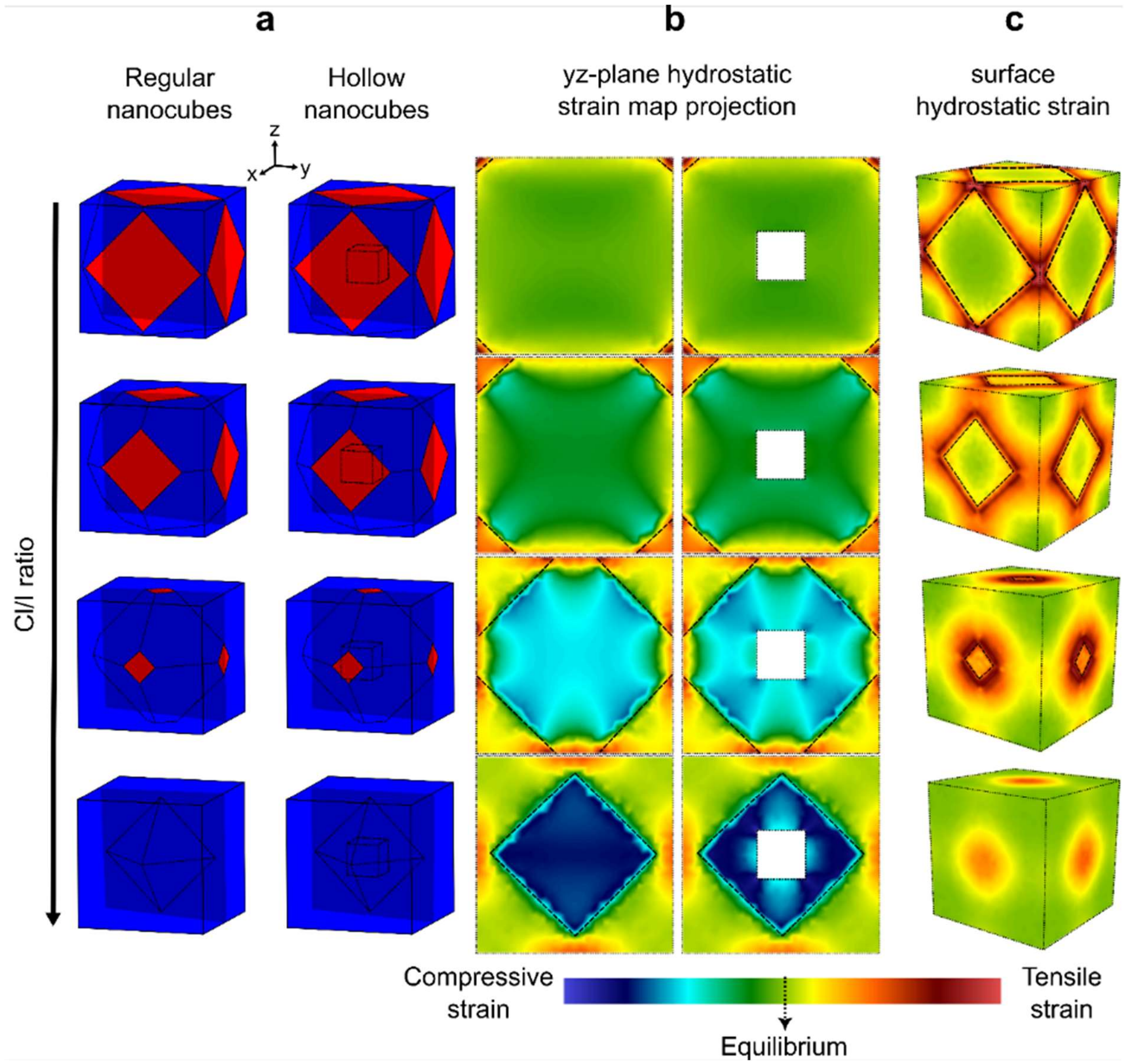

Figure S28. (a) Schematic models of regular (left) and hollow (right) nanocubes with an outer volume of 40×40×40 nm^3 used for performing the linear elastic strain calculations. The hollow nanocubes feature a central 10×10×10 nm^3 void. The shadowed planes within the 3D models indicate the specific *yz*-cross-sections extracted for the 2D projections in panel (b). The downward vertical arrow illustrates the gradual growth of the exchanged $CsPbCl_3$ domains (blue) into the initial $CsPbI_3$ core (red). To simulate the progression of the anion exchange, the inner unexchanged core domain geometrically evolved from a cuboctahedron to an octahedron, while the outer cube boundary remained constant. (b) Internal hydrostatic strain maps across the *yz*-plane projections defined in (a). This specific plane was selected to capture the maximum mechanical distortion, as the highly anisotropic z-direction (*c*-axis) of the orthorhombic core exhibits the most pronounced strain gradients compared to the mechanically near-symmetric *xy*-plane. (c) 3D surface hydrostatic strain maps demonstrating localized tensile strain accumulation arising from $CsPbCl_3$. Comparable strain profiles are observed for both geometries. Dotted lines delimit the geometry boundaries, whereas the dashed lines separate the core and shell domains. The color scale corresponds to the dimensionless hydrostatic strain, with limits of ± 0.13 (compressive strain in dark blue, tensile strain in dark red) and green representing the unstrained equilibrium lattice.

S1. Computational Methods for Figure S28.

S1-1. Geometric modeling and meshing

The 3D geometric models of the core-shell nanocubes were generated employing the OpenCASCADE geometry kernel within the open-source finite-element mesh generator Gmsh. [1] The outer dimensions of the nanocube were fixed at 40 × 40 × 40 nm^3. To simulate the progression of the anion exchange, the inner unexchanged $CsPbI_3$ core domain (Ω_{core}) was geometrically defined by the intersection of the outer cube with a parametrically scaled octahedron.

To guarantee a perfectly conformal mesh at the phase boundaries – a strict requirement for ensuring continuous displacement fields ($\mathbf{u}$) across the heterostructure – the core and shell domains were topologically united using Boolean fragmentation. This forced the generation of shared boundary nodes at the exact interface of the two domains. For models incorporating an internal void, a central 10 × 10 × 10 nm^3 cubic cavity was explicitly subtracted from the core volume utilizing a Boolean difference operation before fragmentation. The continuous geometry was subsequently discretized into 3D tetrahedral elements. Physical volume tags were assigned to the discrete domains (internal core = 1, outer shell = 2) and the mesh was exported via the meshio library into the XDMF format to preserve subdomain markers for the spatially dependent stiffness tensors in the finite element solver.

S1-2. Continuum Mechanics Formulation

The internal stress and strain distributions arising from the lattice mismatch between the unexchanged core and the exchanged domains were calculated by means of a linear elastic continuum model. Geometrically, the exchanged $CsPbCl_3$ region occupies the entire volume between the outer cube boundary and the inner core (Ω_{shell}).

The total strain tensor ($\boldsymbol{\epsilon}$) is defined by the symmetric gradient of the displacement field ($\mathbf{u}$):

$$\epsilon_{ij} = \frac{1}{2}\left(\frac{\partial u_i}{\partial x_j} + \frac{\partial u_j}{\partial x_i}\right) \quad . \tag{1}$$

Note that *i* and *j* run from 1 to 3, and they correlate to the three spatial coordinates (1:*x*, 2:*y*, and 3:*z*).

To ensure mathematical consistency across the domains while reflecting the physical crystallography, the core was modeled using a pseudocubic approximation of the orthorhombic $CsPbI_3$ phase. The lattice mismatch was introduced exclusively within Ω_{core} as a uniform diagonal eigenstrain tensor (ϵ^*):

$$\epsilon^* = \begin{pmatrix} \frac{a_{pc} - a_s}{a_s} & 0 & 0 \\ 0 & \frac{b_{pc} - b_s}{b_s} & 0 \\ 0 & 0 & \frac{c_{pc} - c_s}{c_s} \end{pmatrix} \quad , \tag{2}$$

where a_{pc}, b_{pc}, c_{pc} are the respective pseudocubic lattice parameters of the core, and a_s, b_s, c_s are the cubic lattice parameters of the shell. The crystallographic axes of the pseudocubic core and cubic shell were assumed to be fully coherent and aligned with the global geometric axes, resulting in negligible shear misfit (off-diagonal terms in Equation (2) are zero).

The constitutive relation determining the Cauchy stress tensor ($\boldsymbol{\sigma}$) incorporates this lattice mismatch. The normal stress components (axial compression/tension – Eqs. (3.1)-(3.3)) and shear stress components (shape distortion – Eqs. (3.4)-(3.6)) within the core domain are explicitly defined as:

$$\sigma_{xx} = C_{11}\left(\epsilon_{xx} - \frac{a_{pc} - a_s}{a_s}\right) + C_{12}\left(\epsilon_{yy} - \frac{b_{pc} - b_s}{b_s}\right) + C_{13}\left(\epsilon_{zz} - \frac{c_{pc} - c_s}{c_s}\right) \quad , \tag{3.1}$$

$$\sigma_{yy} = C_{12}\left(\epsilon_{xx} - \frac{a_{pc} - a_s}{a_s}\right) + C_{22}\left(\epsilon_{yy} - \frac{b_{pc} - b_s}{b_s}\right) + C_{23}\left(\epsilon_{zz} - \frac{c_{pc} - c_s}{c_s}\right) \quad , \tag{3.2}$$

$$\sigma_{zz} = C_{13}\left(\epsilon_{xx} - \frac{a_{pc} - a_s}{a_s}\right) + C_{23}\left(\epsilon_{yy} - \frac{b_{pc} - b_s}{b_s}\right) + C_{33}\left(\epsilon_{zz} - \frac{c_{pc} - c_s}{c_s}\right) \quad , \tag{3.3}$$

$$\sigma_{yz} = \sigma_{zy} = 2C_{44}\epsilon_{yz} \quad , \tag{3.4}$$

$$\sigma_{xz} = \sigma_{zx} = 2C_{55}\epsilon_{xz} \quad , \tag{3.5}$$

$$\sigma_{xy} = \sigma_{yx} = 2C_{66}\epsilon_{xy} \quad , \tag{3.6}$$

The system was solved by enforcing the equilibrium equation for force balance ($\nabla \cdot \boldsymbol{\sigma} = 0$):

$$\frac{\partial \sigma_{xx}}{\partial x} + \frac{\partial \sigma_{xy}}{\partial y} + \frac{\partial \sigma_{xz}}{\partial z} = 0 \quad , \tag{4.1}$$

$$\frac{\partial \sigma_{yx}}{\partial x} + \frac{\partial \sigma_{yy}}{\partial y} + \frac{\partial \sigma_{yz}}{\partial z} = 0 \quad , \tag{4.2}$$

$$\frac{\partial \sigma_{zx}}{\partial x} + \frac{\partial \sigma_{zy}}{\partial y} + \frac{\partial \sigma_{zz}}{\partial z} = 0 \quad . \tag{4.3}$$

Eqs. (4.1)-(4.3) represent the x-, y-, and z-direction components, respectively, of the equilibrium equation.

S1-3. Finite Element Implementation

The governing equilibrium equation ($\nabla \cdot \boldsymbol{\sigma} = 0$) was solved using the open-source computing platform FEniCS (via the Python interface).[2] The discrete tetrahedral mesh was mapped to a first-order Continuous Galerkin function space (degree 1). The problem was formulated in its variational weak form:

$$\int_{\Omega} \epsilon(\mathbf{v}) : \mathbb{C} : \epsilon(\mathbf{u}) d\Omega = \int_{\Omega_{\text{core}}} \epsilon(\mathbf{v}) : \mathbb{C} : \epsilon^* d\Omega \quad , \tag{5}$$

where $\mathbf{u}$ is the trial displacement field, $\mathbf{v}$ is the test function, ϵ is the total strain tensor, and $\mathbb{C}$ is the spatially dependent, piecewise elastic stiffness tensor mapping the respective orthorhombic ($\mathbb{C}_{\mathrm{core}}$) and cubic ($\mathbb{C}_{\mathrm{shell}}$) parameters in **Table S6** to their physical subdomains. $\mathbb{C}_{\mathrm{core}}$ and $\mathbb{C}_{\mathrm{shell}}$ are explicitly given – in Voigt notation – by:

$$\mathbb{C}_{\mathrm{core}} = \begin{pmatrix} C_{11} & C_{12} & C_{13} & 0 & 0 & 0 \\ C_{12} & C_{22} & C_{23} & 0 & 0 & 0 \\ C_{13} & C_{23} & C_{33} & 0 & 0 & 0 \\ 0 & 0 & 0 & C_{44} & 0 & 0 \\ 0 & 0 & 0 & 0 & C_{55} & 0 \\ 0 & 0 & 0 & 0 & 0 & C_{66} \end{pmatrix} , \tag{5.1}$$

$$\mathbb{C}_{\mathrm{shell}} = \begin{pmatrix} C_{11} & C_{12} & C_{12} & 0 & 0 & 0 \\ C_{12} & C_{11} & C_{12} & 0 & 0 & 0 \\ C_{12} & C_{12} & C_{11} & 0 & 0 & 0 \\ 0 & 0 & 0 & C_{44} & 0 & 0 \\ 0 & 0 & 0 & 0 & C_{44} & 0 \\ 0 & 0 & 0 & 0 & 0 & C_{44} \end{pmatrix} . \tag{5.2}$$

To eliminate rigid body translation and isolate the purely internal eigenstrain deformation, a single pointwise Dirichlet boundary condition ($\mathbf{u} = 0$) was applied. For the regular nanocubes, this kinematic constraint was located at the center of the structure; for the hollow nanocubes, the constraint was anchored to a coordinate on the internal void wall. The linear system was evaluated employing a direct sparse solver (LU decomposition). Following the calculation of the displacement field, the resulting hydrostatic strain field was projected onto a degree-0 Discontinuous Galerkin (DG) function space for element-wise visualization and extraction.

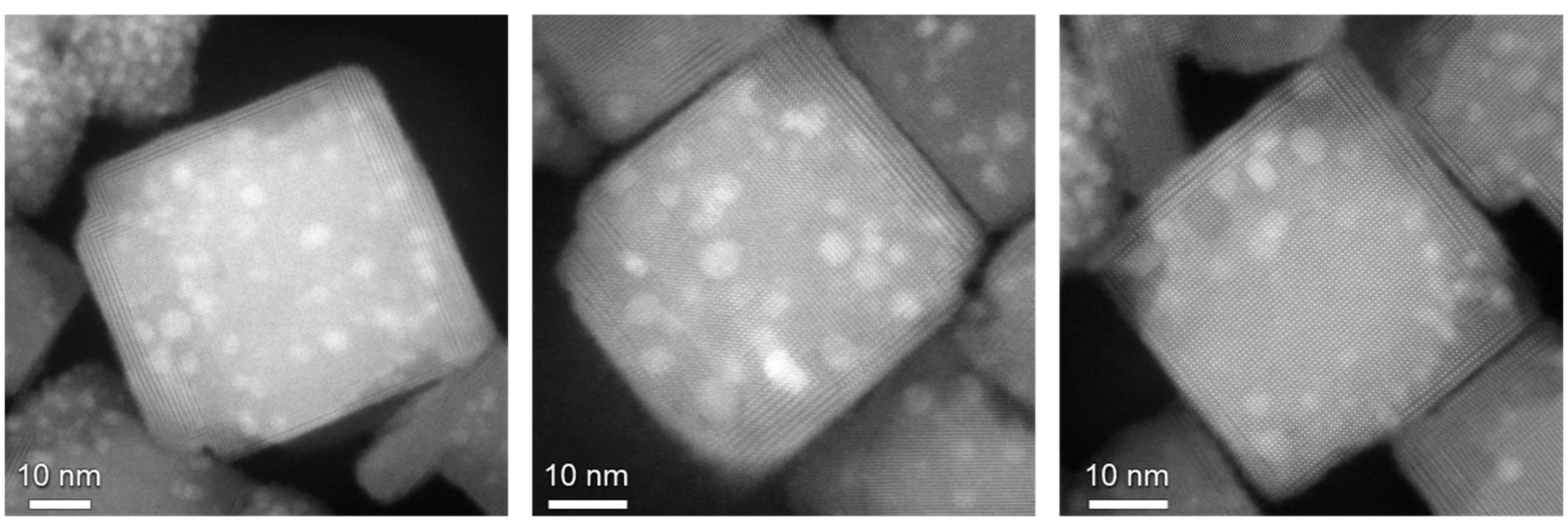

Figure S29. Atomic resolution HAADF-STEM images of $CsPbI_3/CsPbCl_3$ (I/Cl = 0.2) regular nanocubes.

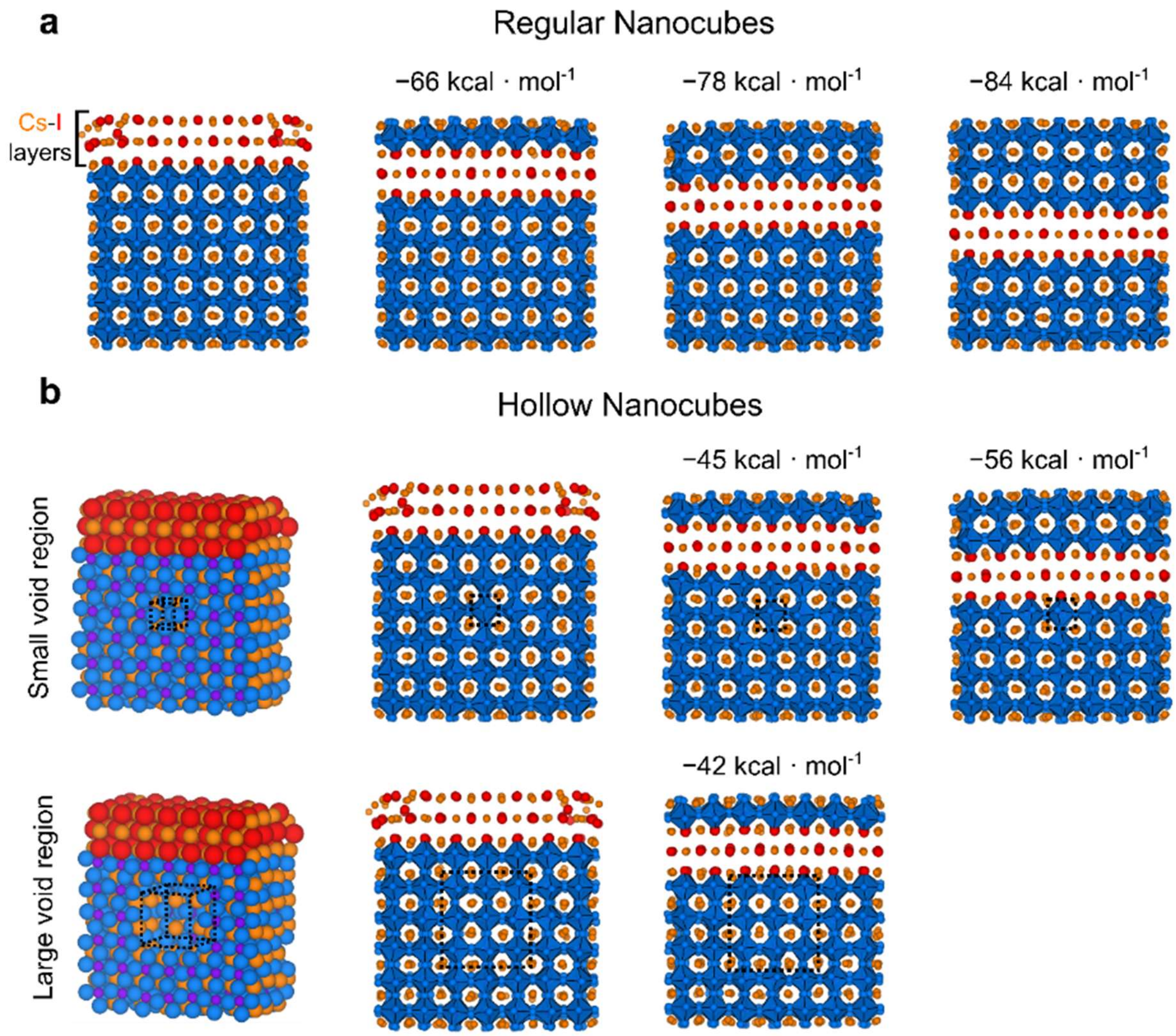

Figure S30. (a) Atomistic models of regular solid nanocubes ($Cs_{363}Pb_{294}Cl_{966}$ – $Cs_{177}I_{162}$) showing the progressive stabilization of the system as the Cs-I planar defect is shifted from the surface into the central region. In all representations, the elements are color-coded as in the elemental maps in the main text: Cs (orange), Pb (purple), Cl (blue), and I (red), with the Cl^- octahedra also rendered in blue. The values above the structures indicate the relative stabilization energy of each configuration compared to the initial surface-located state. (b) Equivalent atomistic models for hollow nanocubes containing small ($Cs_{365}Pb_{293}Cl_{966}$ – $Cs_{177}I_{162}$, top row) and large ($Cs_{363}Pb_{267}Cl_{912}$ – $Cs_{177}I_{162}$, bottom row) central voids. Each sequence begins with a cross-sectional 3D space-filling view of the initial configuration to explicitly visualize the empty internal regions, which are delineated by dashed black boxes. Relative stabilization energies for each configuration are provided above the respective models.

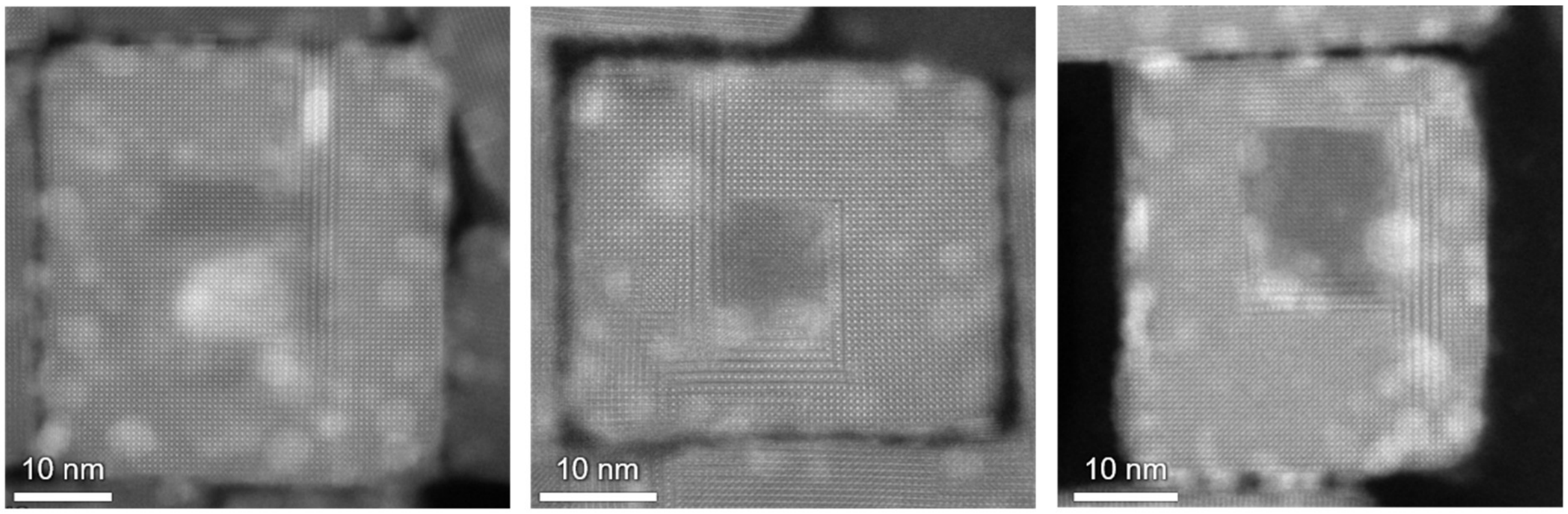

Figure S31. Atomic resolution HAADF-STEM images of hollow $CsPbI_3/CsPbCl_3$ (I/Cl = 0.2) nanocubes.

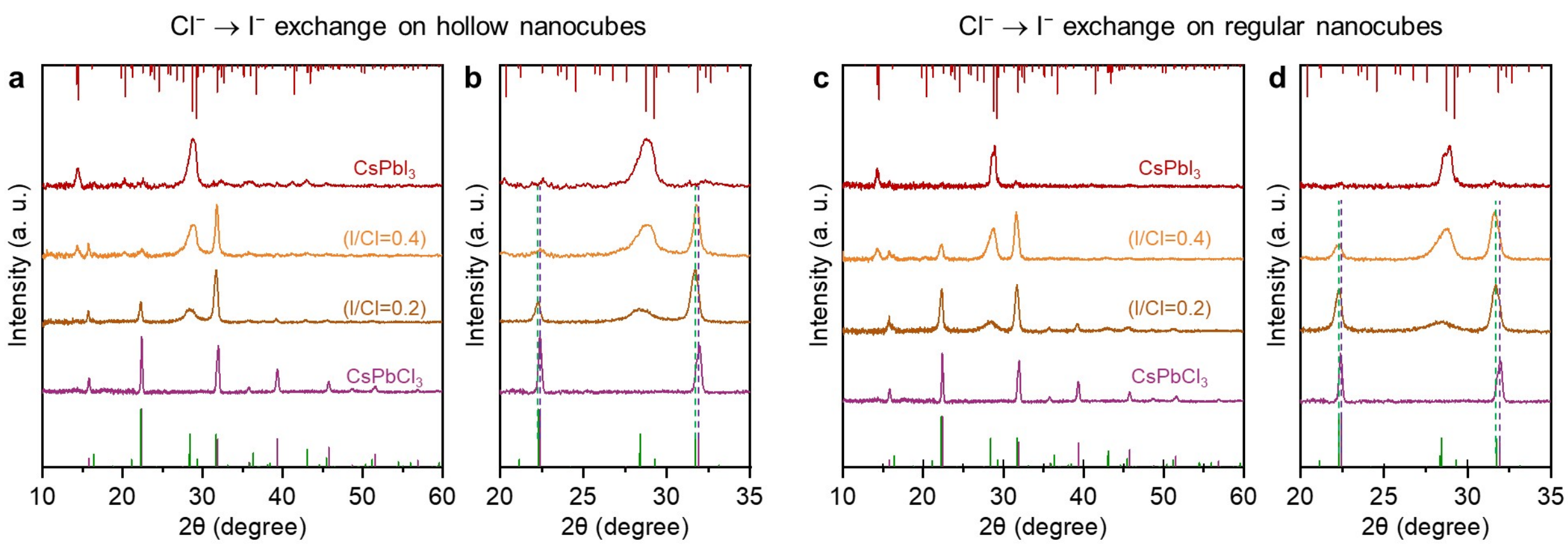

Figure S32. XRD patterns of (a, b) hollow $CsPbCl_3$, $CsPbCl_3/CsPbI_3$ (I/Cl = 0.2, 0.4) and $CsPbI_3$ nanocubes and (c,d) regular $CsPbCl_3$, $CsPbCl_3/CsPbI_3$ (I/Cl = 0.2, 0.4) and $CsPbI_3$ nanocubes in $Cl^- \rightarrow I^-$ exchange. The green reference marks are for the $Cs_2PbI_2Cl_2$ RP phase.[3]

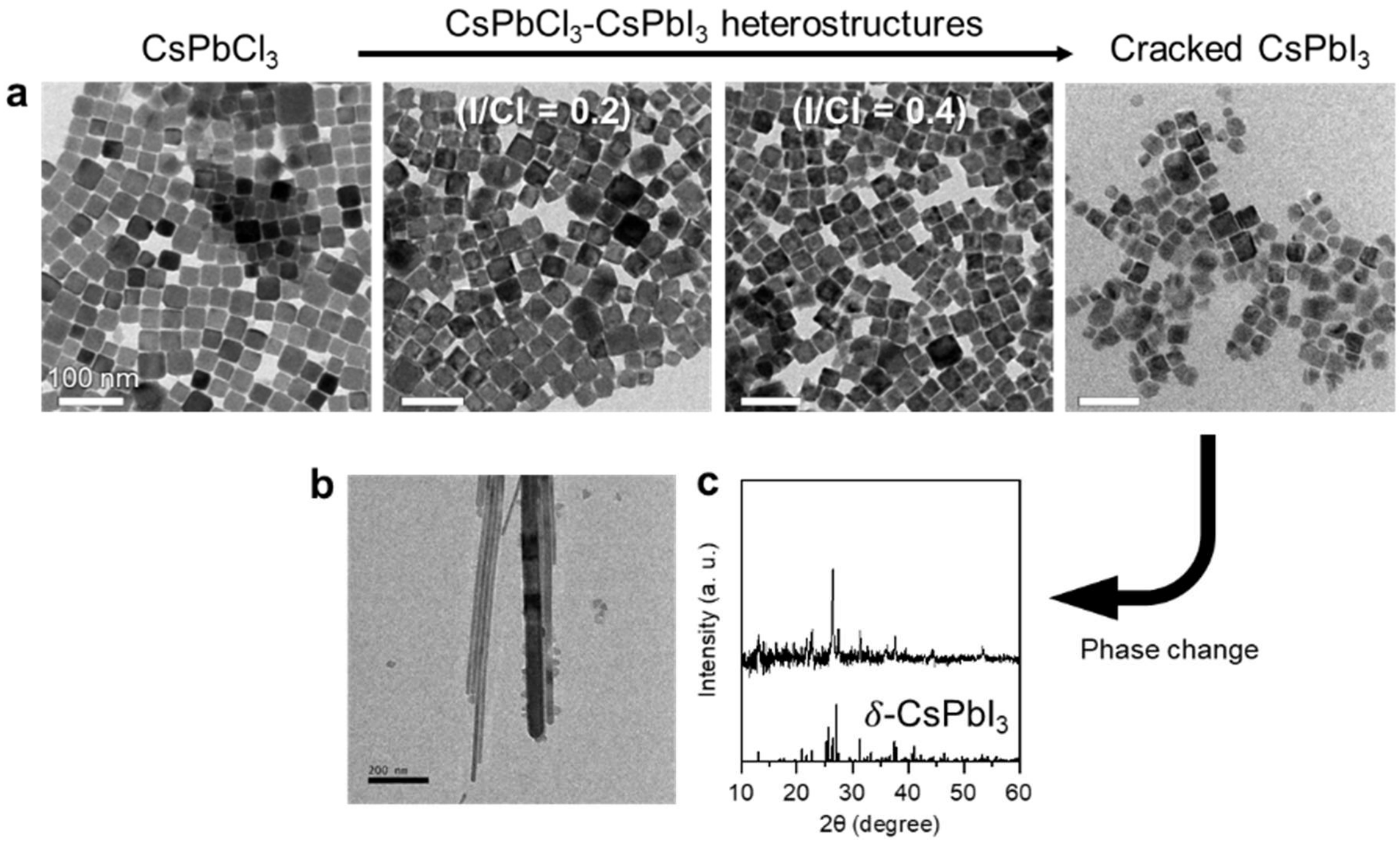

Figure S33. (a) TEM images of regular $CsPbCl_3$, $CsPbI_3/CsPbCl_3$ (I/Cl = 0.2 and 0.4) and $CsPbI_3$ nanocubes. The scale bar in panel a is 100 nm. (b) TEM image and (c) XRD of $CsPbI_3$ nanocubes after 2 hours. The cracked $CsPbI_3$ nanocrystals are highly unstable and rapidly transformed into the δ-$CsPbI_3$ phase (ICSD number 35640).

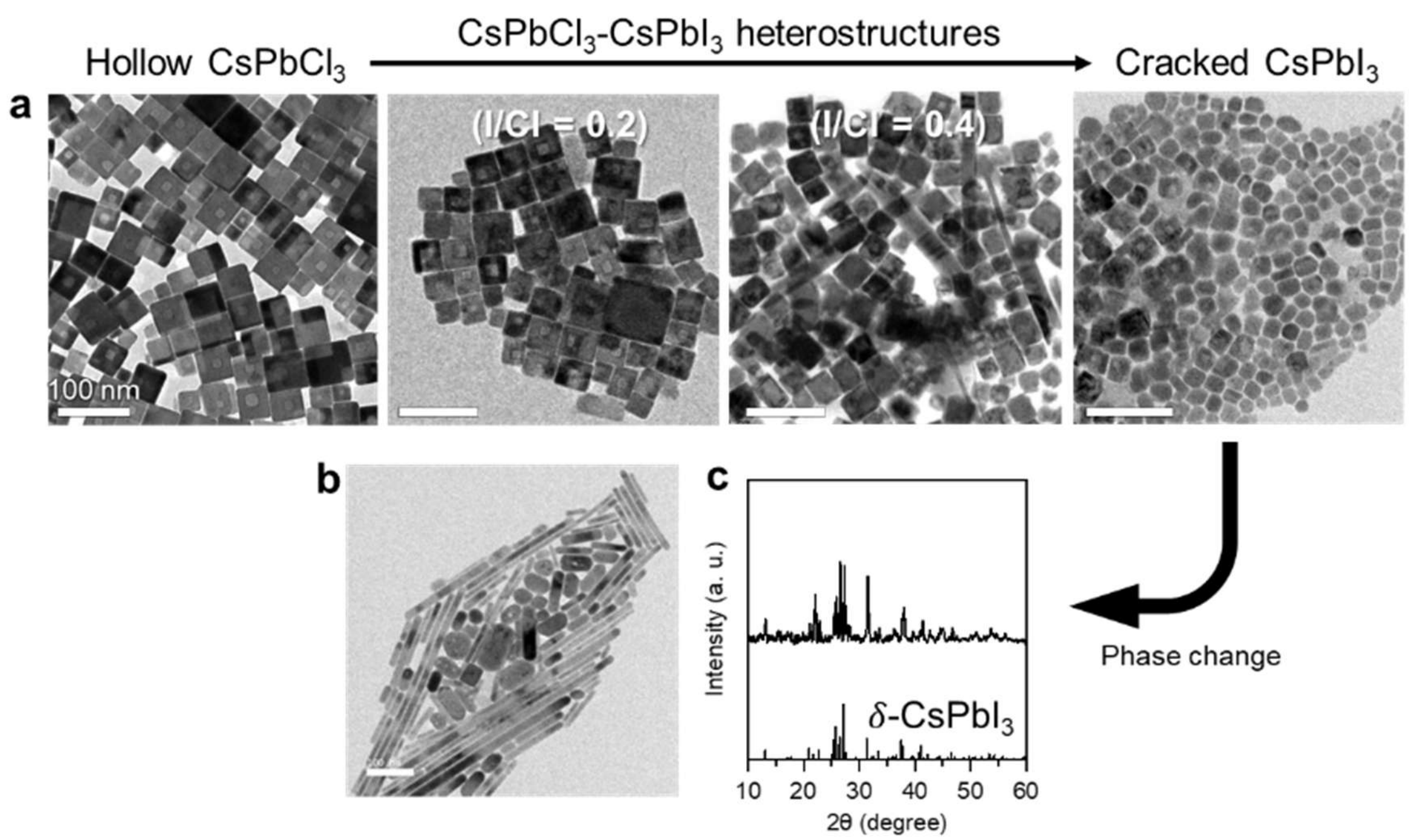

Figure S34. (a) TEM images of hollow $CsPbCl_3$, $CsPbI_3/CsPbCl_3$ (I/Cl = 0.2 and 0.4) and $CsPbI_3$ nanocubes. The scale bar in panel a is 100 nm. (b) TEM image and (c) XRD of $CsPbI_3$ nanocubes after 2 hours. The cracked $CsPbI_3$ nanocrystals are highly unstable and rapidly transformed into the δ-$CsPbI_3$ phase.

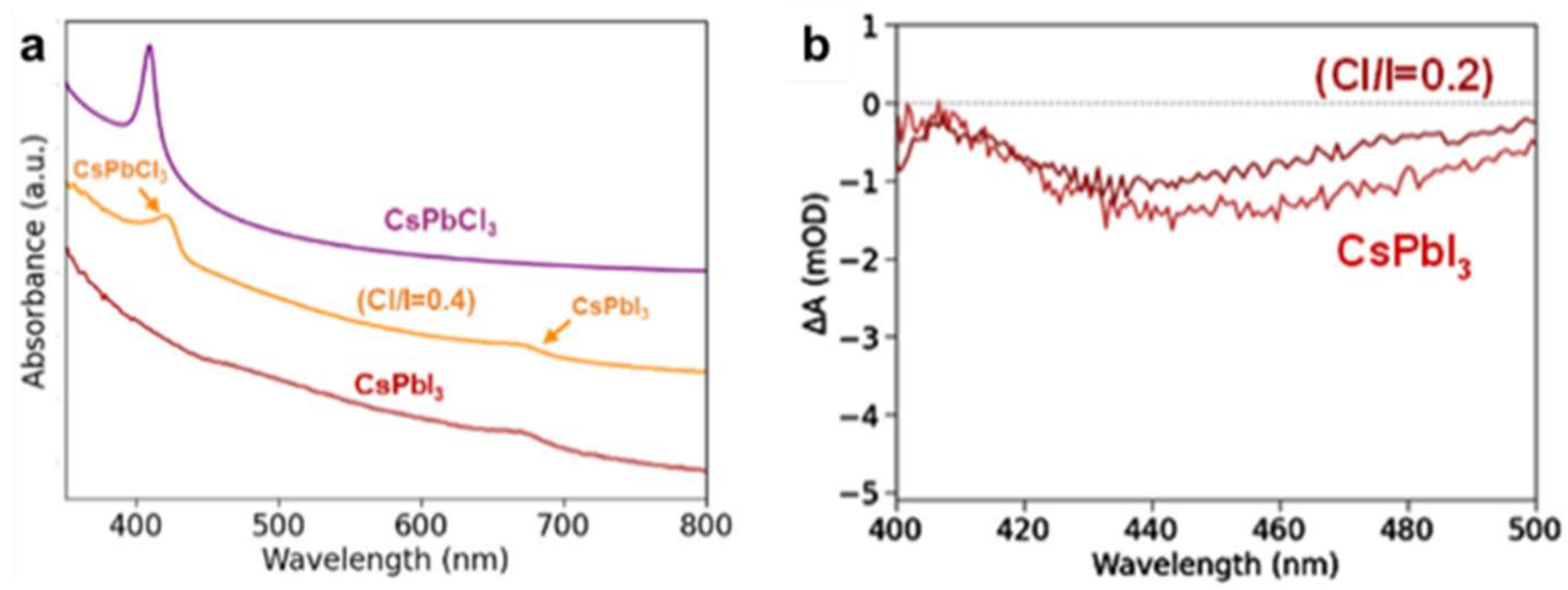

Figure S35. (a) Steady-state absorption of hollow $CsPbI_3$, intermediate $CsPbI_3/CsPbCl_3$ (Cl/I = 0.4), and $CsPbCl_3$ nanocubes, highlighting the presence of both Cl and I phase excitonic absorption peaks in the Cl/I = 0.4 sample. (b) Transient absorption spectra of the partially exchanged hollow nanocrystal sample with Cl/I = 0.2 showing the absence of any $CsPbCl_3$-related bleach (photoinduced transmission), pump fluence 100 µJ/cm^2. For reference, the bleach of $CsPbCl_3$ in heterostructures with Cl/I = 0.4 appears at 430 nm (Figure 8a in the main text).

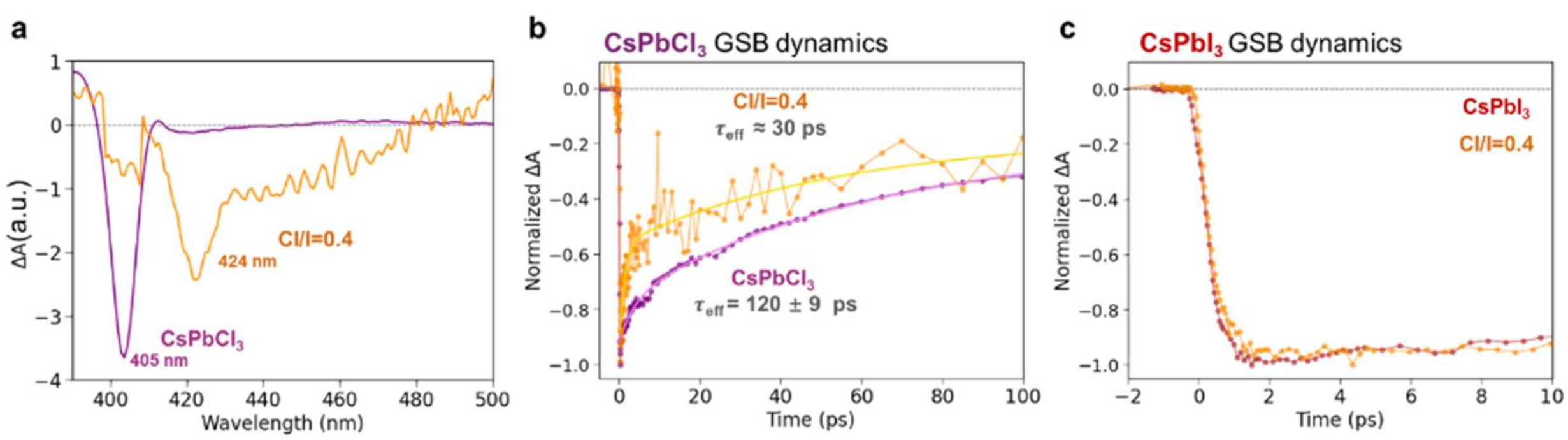

Figure S36. (a) Transient absorption spectra of regular nanocubes of pure $CsPbCl_3$ and intermediate $CsPbI_3/CsPbCl_3$ heterostructures (Cl/I = 0.4), excitation fluence of 100 µJ/cm^2. (b) Transient absorption dynamics at the two minima of spectra shown in (a). (c) Comparison of the dynamics of the I-phase bleach in the regular nanocubes of pure $CsPbI_3$ and intermediate $CsPbI_3/CsPbCl_3$ heterostructures (Cl/I = 0.4).

Table S1. PL peak and full width at half maximum (FWHM) of the PL peak of hollow $CsPbBr_3$, $CsPb(Br:Cl)_3$ (Cl/Br = 0.2, 0.4, 0.6, 0.8) and $CsPbCl_3$ for both hollow and regular nanocubes.

Morphology	Ratio of anions	Peak max position (eV)	FWHM (eV)
Hollow nanocubes	Start $CsPbBr_3$	2.38	0.07
	Cl/Br = 0.2	2.49	0.14
	Cl/Br = 0.4	2.60	0.14
	Cl/Br = 0.6	2.69	0.15
	Cl/Br = 0.8	2.80	0.10
	Final $CsPbCl_3$	3.00	0.08
Regular nanocubes	Start $CsPbBr_3$	2.38	0.07
	Cl/Br = 0.2	2.49	0.07
	Cl/Br = 0.4	2.62	0.08
	Cl/Br = 0.6	2.73	0.08
	Cl/Br = 0.8	2.85	0.08
	Final $CsPbCl_3$	3.00	0.08

Table S2. Calculation of volume of unit cell in orthorhombic $CsPbI_3$ (ICSD number 69423), orthorhombic $CsPbBr_3$ (ICSD number 97851) and cubic $CsPbCl_3$ (ICSD number 29072) phases. Calculation of volume reduction percentages from $CsPbI_3$ to $CsPbCl_3$ or $CsPbBr_3$ phase, and from $CsPbBr_3$ to $CsPbCl_3$ phase. * indicates four Pb atoms in the orthorhombic unit cell.

Perovskite phase	Parameters in the unit cell (Å)	Volume of unit cell (Å^3)	Volume reduction from $CsPbI_3$	Volume reduction from $CsPbBr_3$
Orthorhombic $CsPbI_3$	a=8.85600, b=8.57600, c=12.47200	947.24*	–	–
Orthorhombic $CsPbBr_3$	a=8.2550, c=8.2070, b=11.7590	796.66*	16%	–
Cubic $CsPbCl_3$	a=b=c=5.6050	176.09	26%	12%

Table S3. Fitting parameters of the PL decay spectra of the samples in Br^- to Cl^- exchange reaction. Biexponential decay was used for the fitting procedures. The PL decay spectra were fitted on the Edinburgh FLS900 fluorescence software. The reported τ_{avg} is an amplitude-weighted value calculated as:

$$\tau_{avg} = \frac{B_1\tau_1 + B_2\tau_2}{B_1 + B_2}$$

Br^- to Cl^- Samples	τ_{avg} (ns)	τ_1 (ns)	B_1 (%)	τ_2 (ns)	B_2 (%)
**Hollow nanocubes**					
$CsPbBr_3$	80	21.2 ± 0.6	34	112.6 ± 1.1	66
$CsPb(Br:Cl)_3$(Cl/Br = 0.4)	32	4.1 ± 0.1	45	59.3 ± 0.8	55
$CsPb(Br:Cl)_3$(Cl/Br = 0.6)	25	3.8 ± 0.1	39	38.6 ± 0.8	61
$CsPbCl_3$	14	2.1 ± 0.1	54	26.4± 0.4	46
**Regular nanocubes**					
$CsPbBr_3$	55	13.6 ± 0.2	49	95.3 ± 1.3	51
$CsPb(Br:Cl)_3$(Cl/Br = 0.4)	18	4.9 ± 0.1	57	36.6 ± 0.6	43
$CsPb(Br:Cl)_3$(Cl/Br = 0.6)	12	2.7 ± 0.1	59	25.4 ± 0.4	41
$CsPbCl_3$	9	1.9 ± 0.1	58	19.5 ± 0.3	42

Table S4. Fitting parameters of the PL decay spectra of the samples in I^- to Br^- exchange reaction.

I^- to Br^- Samples	τ_{avg} (ns)	τ_1 (ns)	B_1 (%)	τ_2 (ns)	B_2 (%)
**Hollow nanocubes**					
$CsPbI_3$	442	50.0 ± 4.3	4	458.2 ± 14.1	96
$CsPb(I:Br)_3$(Br/I = 0.2)	337	42.6 ± 3.7	3	344.1 ± 7.6	97
$CsPb(I:Br)_3$(Br/I = 0.4)	251	45.5± 3.4	7	265.2± 4.3	93
$CsPbBr_3$	80	21.2 ± 0.6	34	112.6 ± 1.1	66
**Regular nanocubes**					
$CsPbI_3$	293	50.0 ± 2.9	8	313.1 ± 5.9	92
$CsPb(I:Br)_3$(Br/I = 0.2)	178	50.0 ± 1.1	17	203.8 ± 3.2	83
$CsPb(I:Br)_3$(Br/I = 0.4)	132	42.1 ± 0.9	29	168.6± 2.0	71
$CsPbBr_3$	55	13.6 ± 0.2	49	95.3 ± 1.3	51

Table S5. Fitting parameters of the PL decay spectra of the samples in I^- to Cl^- exchange reaction.

I^- to Cl^- Samples	τ_{avg} (ns)	τ_1 (ns)	B_1 (%)	τ_2 (ns)	B_2 (%)
			**Hollow nanocubes**		
$CsPbI_3$	442	50.0 ± 4.3	4	458.2 ± 14.1	96
Cl/I = 0.2	307	47.7 ± 1.9	12	341.0 ± 8.4	88
Cl/I = 0.4	241	39.2 ± 1.7	17	280.7 ± 7.6	83
$CsPbCl_3$	10	2.1 ± 0.1	49	17.9 ± 1.4	51
			**Regular nanocubes**		
Start $CsPbI_3$	293	50.0 ± 2.9	8	313.1 ± 5.9	92
Cl/I = 0.2	194	48.7± 2.1	16	220.8± 3.6	84
Cl/I = 0.4	176	45.9 ± 1.4	24	216.1 ± 3.9	76
$CsPbCl_3$	9	2.9 ± 0.1	73	26.3 ± 1.2	27

Table S6. Elastic stiffness constants (C_{ij}) and lattice parameters of the cubic $CsPbCl_3$ and orthorhombic $CsPbI_3$ phases utilized in the continuum mechanical models.[4, 5]

Description	Parameter	$CsPbCl_3$ (cubic)[4]	$CsPbI_3$ (orthorhombic)[5]
Longitudinal stiffness along x	C_{11}	48.35 GPa	33.07 GPa
Longitudinal stiffness along y	C_{22}	C_{11}	34.48 GPa
Longitudinal stiffness along z	C_{33}	C_{11}	45.48 GPa
Off-diagonal stiffness (plane xy)	C_{12}	7.88 GPa	18.39 GPa
Off-diagonal stiffness (plane xz)	C_{13}	C_{12}	7.35 GPa
Off-diagonal stiffness (plane yz)	C_{23}	C_{12}	7.19 GPa
Shear stiffness along x	C_{44}	4.92 GPa	6.73 GPa
Shear stiffness along y	C_{55}	C_{44}	6.66 GPa
Shear stiffness along z	C_{66}	C_{44}	17.91 GPa
Lattice parameter along x	a	5.73 Å	8.68 Å
Lattice parameter along y	b	a	8.82 Å
Lattice parameter along z	c	a	12.53 Å
Pseudo-cubic lattice parameter along x	a_{pc}	a	$a/\sqrt{2}$
Pseudo-cubic lattice parameter along y	b_{pc}	b	$b/\sqrt{2}$
Pseudo-cubic lattice parameter along z	c_{pc}	c	$c/2$

Table S7. Fitting parameters for Cl-tuned probe dataset (probe spectral range ca. 390-500 nm) in $CsPbCl_3$ and $CsPbI_3/CsPbCl_3$ (Cl/I = 0.4) hollow nanocubes.

Sample	τ_1 [ps]	A_1	τ_2 [ps]	A_2	c	τ_{eff} [ps]
$CsPbCl_3$	10.7 ± 0.8	0.27 ± 0.01	365 ± 30	0.509 ± 0.008	0.084 ± 0.004	240 ± 30
$CsPbI_3/CsPbCl_3$ (Cl/I = 0.4)*	9 ± 1	0.62 ± 0.03	160 ± 70	0.20 ± 0.03	0.18 ± 0.03	45 ± 20

*($CsPbCl_3$ GSB at 430 nm, Cl-range probe dataset)

Table S8. Fitting parameters for I-tuned probe dataset (probe spectral range ca. 500-700 nm) in $CsPbI_3$ and $CsPbI_3/CsPbCl_3$ (Cl/I = 0.4) hollow nanocubes.

Sample	τ_{1_Rise}, [ps]	A_1	τ_2 [ps]	A_2	τ_3 [ps]	A_3	c
$CsPbI_3$	0.28 ± 0.01	-0.96± 0.01	120 ± 20	0.24 ± 0.2	1700 ± 200	0.49 ± 0.02	0.22 ± 0.01
$CsPbI_3/CsPbCl_3$ (Cl/I = 0.4)*	0.42 ± 0.007	-0.98 ± 0.01	150 ± 10	0.35 ± 0.01	1580 ± 100	0.51 ± 0.01	0.122 ± 0.006

*($CsPbI_3$ GSB at 668 nm)

REFERENCES

(1) Geuzaine, C.; Remacle, J.-F. Gmsh: A 3D Finite Element Mesh Generator with Built In Pre- and Post-Processing Facilities. *Int. J. Numer. Methods Eng.* **2009**, *79*, 1309−1331.

(2) Logg, A.; Mardal, K.-A.; Wells, G. Automated Solution of Differential Equations by The Finite Element Method*: The FEniCS book*; Springer Science & Business Media, **2012**.

(3) Li, J.; Yu, Q.; He, Y.; Stoumpos, C. C.; Niu, G.; Trimarchi, G. G.; Guo, H.; Dong, G.; Wang, D.; Wang, L.; Kanatzidis, M. G. Cs2PbI2Cl2, All-Inorganic Two-Dimensional Ruddlesden–Popper Mixed Halide Perovskite with Optoelectronic Response. *J. Am. Chem. Soc.* **2018**, *140*, 11085−11090.

(4) Aktary, M.; Kamruzzaman, M.; Afrose, R. A comparative study of the mechanical stability, electronic, optical and photocatalytic properties of $CsPbX_3$ (X = Cl, Br, I) by DFT calculations for optoelectronic applications. *RSC Adv.* **2022**, *12*, 23704−23717.

(5) Li, Z.; Qin, Y.; Dong, L.; Li, K.; Qiao, Y.; Li, W. Elastic and electronic origins of strain stabilized photovoltaic γ-$CsPbI_3$. *Phys. Chem. Chem. Phys.* **2020**, *22*, 12706−12712.
